# Establishment of CRISPR/Cas9 Genome Editing in *Ganoderma boninense* and Functional Validation of Hydrophobin-2 as a Virulence Determinant

**DOI:** 10.3390/jof12080594

**Published:** 2026-08-11

**Authors:** Anis Farhan Fatimi Ab Wahab, Mohd Azinuddin Ahmad Mokhtar, Sharmilah Vetaryan, Yang Ping Lee

**Affiliations:** 1 Geoinformatics, Genomics & Bioinformatics Department, FGV Innovation Centre (Biotechnology), FGV R&D Sdn. Bhd., PT. 23417 Lengkuk Teknologi, Bandar Enstek 71760, Negeri Sembilan, Malaysia; sharmilah.v@fgvholdings.com (S.V.); yangp.lee@fgvholdings.com (Y.P.L.); 2 Plant Breeding Unit, Tun Razak Agricultural Research Centre (PPPTR), FGV R&D Sdn. Bhd., Jerantut 27000, Pahang, Malaysia; azinuddin.am@fgvholdings.com

**Keywords:** *Ganoderma boninense*, oil palm, basal stem rot, upper stem rot, CRISPR/Cas9 gene editing; gene knock out

## Abstract

Oil palm is a major commodity crop in Southeast Asia, particularly in Malaysia and Indonesia, but its productivity is severely threatened by basal stem rot (BSR) and upper stem rot (USR) caused by the white-rot fungus *Ganoderma boninense*. Infected palms can lose up to 80% yield and die within 6–24 months (young) or 2–3 years (mature). Despite extensive field management efforts, disease incidence continues to rise, especially after replanting. Understanding infection mechanisms and validating fungal virulence factors are crucial for effective control, yet functional genomics in *G. boninense* has been limited. Previous RNAi-based gene silencing provided initial insights but was constrained by off-target effects and transient activity. Here, we report the first successful application of CRISPR/Cas9 genome editing in *G. boninense* for functional gene studies. Two genes were targeted: *pyrG*, essential in uridine monophosphate (UMP) biosynthesis; and *hyd-2*, encoding a hydrophobin, a potential virulence determinant implicated in host invasion. The disruption of *pyrG* produced a uracil auxotroph, and the knockout was validated by screening on 5-FOA and validated our system as a functional molecular tool. Disruption of *hyd-2* reduced infection capability of the fungus by ~72% to ~91% in vitro. Mutations in both gene disruptions, including insertions, deletions, and substitutions, were confirmed by sequencing. Sequencing analysis also revealed incomplete editing events, as wild-type gene sequences were detected alongside edited alleles in the mutants. Future enhancements should focus on improving editing efficiency of the system. This work establishes a robust platform for functional genetic analysis and dissecting pathogenicity in *G. boninense*, ultimately advancing strategies to mitigate basal stem rot disease in oil palm.

## 1. Introduction

The African oil palm (*Elaeis guineensis* Jacq.) is a monoecious species from the Arecaceae family and represents a key global commodity crop [1]. As of 2021, global palm oil production reached 81.52 million tonnes [2], with Malaysia and Indonesia as the leading exporters [3]. Malaysia alone contributed 20.9 million metric tons, 26% of global supply, across 5.85 million hectares of plantations, with Sabah and Sarawak being the largest producing regions [4,5]. Oil palm continues to experience strong global demand due to its high yield, cost-efficiency, and versatility [6]. Consequently, ensuring the sustainability of the industry is critical [7,8].

One of the major threats to oil palm sustainability is Basal Stem Rot (BSR), primarily caused by *Ganoderma boninense*. BSR causes significant economic losses in Malaysia, estimated at RM 1.5 to 2.0 billion annually [9,10]. These losses arise due to severe yield reduction, additional costs from replanting, land sanitation, and prolonged land unproductivity. Following infection, affected fields typically undergo a fallow period ranging from 6 months to 2 years to reduce residual inoculum and enhance field sanitation [11,12,13].

Current BSR management practices range from cultural and mechanical methods to chemical treatments, biological control, and early detection, with a success rate of 20–85% depending on timing, disease severity, and environmental conditions. This highlights the need for more comprehensive and integrated disease management strategies [10,14,15].

A deeper molecular understanding of *G. boninense* is essential to support this goal. However, functional genomic studies are constrained by several factors, including inefficient genetic transformation systems, limited selectable markers, slow fungal growth, long experimental durations, gene redundancy, high isolate variability, and insufficient omics resources [16,17,18,19,20]. These constraints have hindered efforts to elucidate the mechanisms by which this fungus invades host cells, particularly through enzymes and proteins acting as virulence factors, such as cell wall-degrading enzymes, effectors, small secreted proteins, lipases, and proteases [21,22].

Previously, genomic studies on *G. boninense* using RNA interference (RNAi) to silence the *GbERG11* gene encoding Lanosterol 14α-demethylase have been reported, demonstrating the successful use of RNAi for gene silencing in *G. boninense*. This finding marked the first established functional study in this species [23]. RNAi offers advantages in that it only attenuates expression at the transcriptional level, without altering the DNA sequence at the genomic level. Despite its utility, RNAi is hindered by off-target effects and transient gene silencing, which can complicate long-term infection analysis and interfere with accurate gene function studies [23,24,25,26].

To overcome these limitations, this study aims to establish a CRISPR/Cas9-based genome editing system for *G. boninense*, which has not yet been demonstrated in this species. CRISPR/Cas9 has emerged as a precise and efficient tool for targeted genome modification, offering advantages over older technologies like zinc finger nucleases and TALENs due to its simplicity, specificity, and versatility [27,28,29]. Compared to RNAi, CRISPR/Cas9-based genome editing offers permanent alteration of genomic DNA, allowing for the generation of more stably maintained mutants and mutations for analysis. This system consists of two main components; Cas9 protein and single guide RNA (sgRNA). The principles, advantages and further CRISPR/Cas-based system improvement have been comprehensively discussed and reported [30,31,32].

To date, CRISPR/Cas-mediated genome editing has only been attempted in a limited number of plant-pathogenic basidiomycete fungi. These include *Ustilago maydis* (the causal agent of corn smut) [33], *Ustilago hordei* (covered smut of barley) [34], and *Sporisorium scitamineum* (sugarcane smut) [35], in which CRISPR/Cas9 has been predominantly employed to investigate genes associated with pathogenicity, virulence, and host–pathogen interactions through plasmid- or vector-based expression of Cas9 and single-guide RNA (sgRNA). In contrast, within the genus *Ganoderma*, CRISPR/Cas9 genome editing has been established primarily in *Ganoderma lucidum*. Initial studies employed plasmid-based CRISPR/Cas9 systems for targeted gene disruption [36,37], followed by the development of more advanced approaches, including homologous recombination (HDR)-mediated gene replacement and insertion [37], dual-sgRNA-mediated large-fragment deletion [36], and CRISPR-assisted in situ complementation for functional validation [38]. More recently, genome editing has been achieved using pre-assembled Cas9–guide RNA ribonucleoprotein (Cas9–gRNA RNP) complexes delivered into fungal protoplasts [39]. Compared to plasmid-based approaches, this DNA-free editing strategy eliminates the risk of random vector integration and limits the duration of Cas9 activity within the cell, thereby reducing the potential for off-target effects while facilitating efficient, marker-free genome editing.

Despite the considerable economic importance of *G. boninense*, the causal agent of basal stem rot in oil palm, an efficient and reproducible CRISPR/Cas9 genome-editing platform has yet to be established for this pathogen. This is in stark contrast to *G. lucidum*, where CRISPR/Cas9 genome editing has been extensively developed and applied. The absence of a comparable system for *G. boninense* represents a significant gap in the functional genomics toolbox, limiting investigations into the molecular mechanisms underlying pathogenicity, host adaptation, and disease development, as well as hindering the functional validation of candidate virulence genes and the development of effective disease management strategies.

In this study, we developed a CRISPR/Cas9 system for *G. boninense* by synthesising the *cas9* gene, which was subsequently cloned into the pGreen0179 plasmid with hygromycin B (*hygB*) as the selection antibiotic. To show that this system can be used for functional analysis in *G. boninense*, we targeted the 5′-monophosphate decarboxylase (*pyrG*) gene in a proof-of-concept analysis and a gene encoding a putative hydrophobin (*hyd*) (an effector protein) for virulence-targeted gene analysis. The *pyrG*/5-fluoroorotic acid (5-FOA) system is a classical negative selection strategy widely used in fungi [40], whereas the selected hydrophobin is predicted to be responsible for coating the hyphae, making them hydrophobic and facilitating surface adherence and penetration into host tissues [41,42,43,44].

## 2. Materials and Methods

- Strain and cultivation

*G. boninense* PER71 was utilised in this study. This strain originated from Peninsular Malaysia and was obtained from the Malaysian Palm Oil Board (MPOB) of Bandar Baru Bangi in Selangor, Malaysia. This strain is a heterokaryon (dikaryotic) strain and is governed by a tetrapolar mating system. The culture was maintained on potato dextrose agar (PDA) (Oxoid, USA), sub-cultured biweekly, and preserved in paraffin oil (Sigma-Aldrich, USA) at room temperature for long-term storage.

b. Culture medium for mycelial propagation

Approximately fifty 8 mm agar plugs from 7-day-old *G. boninense* PER71 mycelia were inoculated into 250 mL of complete yeast media broth (CYMB) (containing 2% glucose, 0.2% peptone, 0.1% yeast extract, 0.05% MgSO_4_·7H_2_O, 0.046% KH_2_PO_4_, and 0.1% K_2_HPO_4_). Cultures were incubated at 28 °C with shaking at 270 rpm for 5 days to promote mycelial proliferation.

c. Construction of *cas9* gene cassette

The *G. lucidum* codon-optimised *cas9* gene (*Glcas9*) cassette was synthesised along with P*_gpd_*, the glyceraldehyde-3-phosphate dehydrogenase promoter originating from *G. lucidum* to drive its expression in *G. boninense*, and T*_pd_*, the pyruvate decarboxylase terminator originating from *Trichoderma reesei*, to terminate transcription. *Eco*RV and *Spe*I restriction sites were included flanking the cassette for future cloning purposes. The *Glcas9* cassette was subsequently inserted into the pGreen0179, resulting in the pGreenGlcas9 construct.

d. Selection of genes and sgRNAs for each targeted gene

Two types of genes were selected for this project: orotidine-5′-monophosphate decarboxylase (*pyrG*) and hydrophobin (*hyd*).

- *pyrG*

The *pyrG* gene was selected as a proof-of-concept to assess the feasibility of using the CRISPR/Cas9 gene-editing system for functional studies. It encodes a key enzyme in the de novo pyrimidine biosynthesis pathway, responsible for converting orotidine 5′-monophosphate (OMP) to uridine 5′-monophosphate (UMP), a crucial step in the production of pyrimidine nucleotides, essential building blocks of RNA and DNA [45,46]. This gene was chosen due to the ease of mutant screening, particularly by identifying uracil auxotrophy and growth sensitivity to media containing 5-FOA. 5-FOA is toxic to cells with a functional *pyrG* gene, as it is converted into 5-fluorouridine monophosphate (5-FUMP), which disrupts RNA and DNA synthesis. Mutants lacking functional *pyrG* cannot convert 5-FOA and therefore survive, allowing for effective selection of gene-edited strains. Other than resistance to 5-FOA, deactivation of the *pyrG* gene results in a uridine auxotrophic phenotype that requires uridine to be supplemented in minimal media (MM) to grow. A minimum inhibitory concentration (MIC) assay was performed on *G. boninense* for 5-FOA to ascertain that a final concentration of 1500 µM was suitable for mutant selection (Figure S1). To search for the *pyrG*-encoded transcripts from a PacBio Iso-seq transcriptome database for *G. boninense* (unpublished data), a *pyrG* reference sequence from *Lentinula edodes* (Accession Number: XM_046233948.1) was used for BLAST (BLAST+ version 2.12.0) analysis against *G. boninense* strain G3 genomic databases (https://www.ncbi.nlm.nih.gov/datasets/genome/GCA_002900995.3/, accessed on 9 March 2022).

ii. Effector protein—hydrophobin

Hydrophobins are small, cysteine-rich effector proteins unique to fungi that play diverse and critical roles in fungal biology and pathogenesis. They contribute to immune evasion, adhesion, colonisation, environmental stress tolerance, and fungal development by modulating host defence responses and masking fungal structures from immune recognition [47].

In this study, nine putative hydrophobin genes, predicted to function as effectors, were successfully identified from our in-house PacBio Iso-Seq transcriptome databases generated using infected roots of clonal oil palm materials harvested at various infection time points—12 h post-inoculation (hpi), 24 hpi, 3 days post-inoculation (dpi), 5 dpi, 9 dpi, and 21 dpi (data unpublished).

To obtain comprehensive insights into the gene expression profiles of the nine identified hydrophobins, quantitative PCR (qPCR) analysis was conducted using preliminary in vitro *Ganoderma*-infected oil palm roots harvested at 3, 5, 9, 21, 28, 35, 42, 49, and 56 dpi. For each time point, three biological replicates were collected and pooled prior to analysis. This analysis aimed to monitor their expression patterns throughout the infection, including at late stages. The housekeeping genes used for fold change analysis are *β-tubulin* (*β-tub*), *40S* subunit (*40S*) and *α-tubulin-1* (*α-tub1*). The primer sequences are provided in Table S1.

Based on the qPCR analysis, a potential hydrophobin gene was selected for functional characterisation using the CRISPR/Cas9 gene editing system. The mutants produced were assessed for their ability to infect the host.

iii. Selection of potential sgRNA for the targeted genes

To design the sgRNA sequences for gene editing, the transcript sequences of *pyrG* and the selected hydrophobin gene, obtained from the PacBio Iso-Seq transcriptome databases, were BLASTed against the *G. boninense* strain G3 genome database to retrieve their corresponding gene sequences. These sequences were then submitted to the online design tool CCTop—CRISPR/Cas9 Target Online Predictor (https://cctop.cos.uni-heidelberg.de/, accessed on 20 April 2022), using *G. lucidum* as the reference genome. Only sgRNAs with high prediction scores were selected. These selected sgRNAs were then subjected to a BLAST search against our in-house PacBio Iso-Seq transcriptome database to identify potential off-target sites. Any sgRNA showing more than four base-pair matches to non-target genes was excluded from further analysis. Additionally, sgRNAs located within intronic regions were also discarded.

iv. Construction of sgRNA templates and in vitro Transcription

The sgRNA cassette, containing *Eco*RI restriction sites at both the 5′ and 3′ ends, a T7 promoter sequence, 20 nucleotides of sgRNA (without the protospacer adjacent motif (PAM) sequence), and 80 nucleotides of scaffold tracrRNA, was directly synthesised by a commercial provider. The synthesised cassette was digested with *Eco*RI and cloned into the pGEM-T Easy vector, resulting in the construct designated as pGEM-sgRNA. This plasmid was subsequently used for sgRNA template preparation.

**Figure 1:**
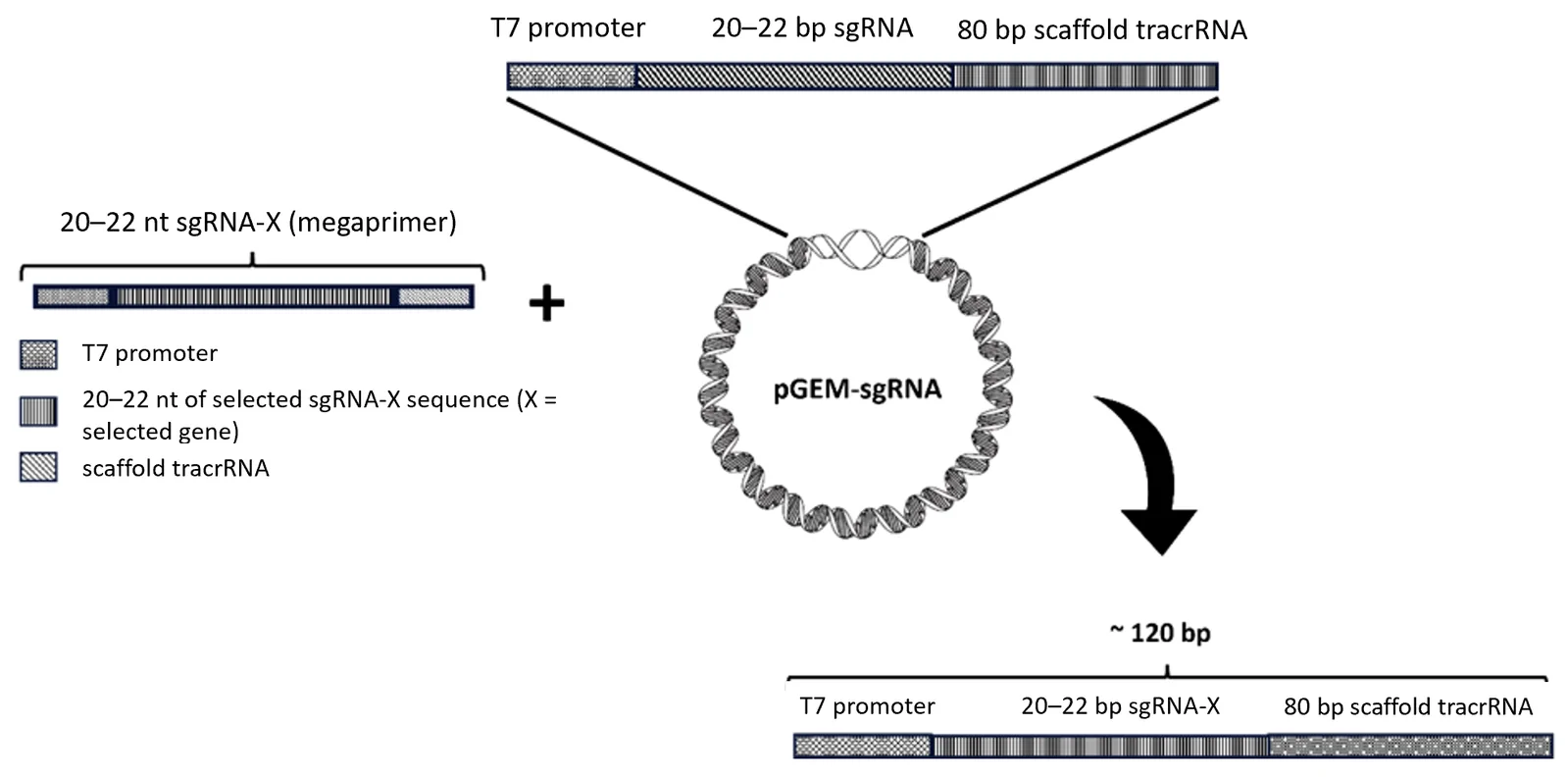
The schematic figure of sgRNA synthesis using the megaprimer approach, incorporating the T7 promoter, sgRNA target sequence, scaffold tracrRNA, and transcription terminator.

In addition, DNA oligos were synthesised as a megaprimer, consisting of a 21 bp T7 promoter sequence, a 20–22 bp sgRNA sequence, and 15 bp of the 5′ scaffold tracrRNA. A reverse primer was also synthesised, comprising the 3′ region of the tracrRNA and four thymidine residues functioning as a transcription terminator. sgRNA synthesis using the megaprimer approach was performed by PCR (MyTaq Polymerase, Bioline, USA), following the program outlined in Table 1. A schematic representation of the sgRNA preparation workflow is shown in Figure 1. The PCR product size of DNA amplicon should be approximately 120 bp.

Prior to the PEG-mediated protoplast transformation, the sgRNA amplicons were transcribed in vitro using the Guide-it™ sgRNA in vitro Transcription and Screening System kit (Takara Bio Inc., Shiga, Japan; Cat. No. 632639), according to the manufacturer’s instructions.

e. Protoplast generation and PEG-mediated transformation.

Protoplast preparation and transformation were conducted following a previously described method [16], with minor modifications. Mycelia were first rinsed with 0.6 M mannitol, then incubated in 30 mL of osmotic medium (OM) (0.6 M mannitol, 0.1 M phosphate buffer pH 5.4, 1% lysing enzymes from *Trichoderma harzianum*, 0.08% Driselase, 0.03% Yatalase, 0.03% lysing enzymes from *Aspergillus* and 0.03% Yatalase) for 3 h at 30 °C with shaking at 110 rpm. The resulting protoplast suspension was filtered through sterile Miracloth covered with a single layer of Kimwipe, followed by centrifugation at 1956× *g* for 30 min at 4 °C. The supernatant was carefully discarded without disturbing the pellet. The protoplast pellet was resuspended in 1 mL of 0.6 M mannitol, transferred to a sterile 1.5 mL microcentrifuge tube, and centrifuged at 1008× *g* for 5 min. This washing step was repeated twice. Finally, the protoplasts were resuspended in 300 μL of mannitol–Tris–CaCl_2_ (MTC) solution (0.6 M mannitol, 100 mM Tris-HCl, pH 8.0, 100 mM CaCl_2_), counted using a hemocytometer, and adjusted to a concentration of 1 × 10^8^ protoplasts/mL for storage or further use.

The isolated protoplasts were subjected to PEG-mediated transformation, based on the protocol by [16] with slight modifications. For each transformation event, approximately 100 μL of protoplasts at a concentration of 1 × 10^7^ protoplasts/mL were utilised. In this study, three sgRNAs were used for each target gene. The purified in vitro-transcribed sgRNA was diluted to a working concentration of 20 ng/µL (~0.5 µM). Subsequently, approximately 1.5 µL of each sgRNA was combined with 4 µg of the pGreenGlCas9 plasmid in a final reaction volume of 10 µL. This mixture was then added to 50 μL of PTC solution (60% *w*/*v* PEG4000, 10 mM Tris-HCl pH 7.5, 50 mM CaCl_2_) and 50 μL of STC solution (0.6 M sorbitol, 10 mM CaCl_2_, 10 mM Tris-HCl pH 7.5), followed by incubation at room temperature for 20 min.

Subsequently, 10 mL of YMSB medium (0.015 M yeast extract, 0.016 M peptone, 0.044 M malt extract, 0.6 M mannitol) was added. Before transferring the mixture to Petri dishes, it was combined with 10 mL of warm 2% YMSB agar (2% agar with the same nutrient composition). Plates were incubated overnight at 28 °C. On the following day, 10 mL of potato dextrose agar (PDA) supplemented with 1500 µM 5-fluoroorotic acid (5-FOA) was overlaid to screen for potential *G. boninense pyrG* mutants. Additionally, 110 μg/mL of hygromycin B was added to screen for *hyd* mutants. Plates were then incubated in the dark at 28 °C for 5 to 7 days until the emergence of putative mutant colonies.

f. Validation of Gene Editing Events: PCR Analysis *pyrG* Auxotrophy, and Hygromycin Resistance (Hyg^R^)

Only putative mutants that successfully grew through at least three generations on the selection medium were selected for genomic DNA extraction and subsequent PCR analysis to validate the gene editing events. The genomic DNA of the selected mutants were extracted using the CTAB method [48], which provides improved recovery of high-molecular-weight genomic DNA from filamentous fungi, yielding material of high quality and purity suitable for downstream analyses [48,49]. The concentration and purity of the extracted genomic DNA were measured using a NanoDrop ND-1000 spectrophotometer (Thermo Fisher Scientific, USA), and DNA integrity was assessed by electrophoresis on a 1% agarose gel in 1× TAE buffer. To detect gene editing events, specific primer pairs were used to amplify the entire targeted gene region, and the PCR products were subsequently sent for Sanger sequencing. The Sanger sequencing results and chromatograms for the mixed population were analysed using online indel prediction tools, such as TIDE (https://tide.nki.nl/) [50] and ICE-SYNTHEGO (https://ice.editco.bio/#/) [51] for initial identification of potential mutants. Potential mutants identified from TIDE and ICE-SYNTHEGO analysis were selected for further cloning and sequencing to identify potential base substitution mutations. All clonal sequencing results were then aligned via multiple sequence alignment to identify regions with mutations.

g. Assessment of the *G. boninense hyd-2* mutants on the ability to infect the host

**Figure 2:**
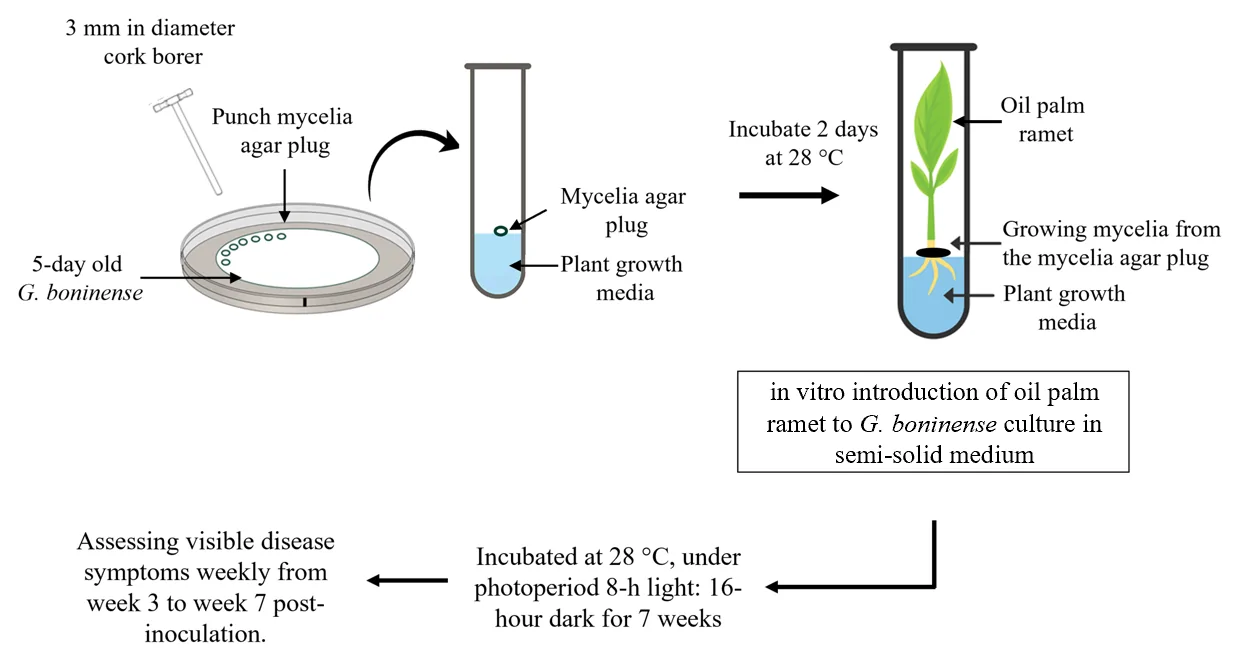
The in vitro inoculation setup showing a 5-day-old mycelial plug (3 mm diameter) of *G. boninense* on solid growth medium in a glass test tube (25 mm × 150 mm), incubated at 28 °C in the dark for 2 days prior to introduction of oil palm ramets. Following inoculation, cultures were maintained at 28 °C under an 8 h light: 16 h dark photoperiod for 7 weeks, with DSI scores recorded from week 3 to week 7.

The validated *G. boninense* ∆*hyd-2* mutants were subjected to a preliminary in vitro *Ganoderma* infection assay using one-month-old rooted ramets, with wild-type (WT) *G. boninense* PER71 serving as the control. The experiment included six biological replicates. A single 5-day-old mycelial plug (3 mm in diameter) was inoculated onto 10 mL of solid growth medium in glass test tubes (25 mm × 150 mm) and incubated in the dark at 28 °C for 2 days before introducing the ramets (Figure 2).

Foliar disease symptoms on each ramet, infected by either the WT or the *G. boninense* ∆*hyd-2* mutants, were recorded weekly from week 3 to week 7 post-infection. The Disease Severity Index (DSI) will be determined based on disease symptom scoring using a 0–5 scale, where 0 = healthy leaves with no visible symptoms; 1 = less than 50% of the leaf area exhibiting a yellowish or brownish colour (i.e., partial yellowing or browning with green tissue still visible); 2 = more than 50% of the leaf area exhibiting a yellow or brown colour (i.e., the affected area is completely yellow or brown); 3 = 30–50% of the leaf area exhibiting a yellow or brown colour; 4 = more than 50% of the leaf area exhibiting a yellow or brown colour; and 5 = dead leaves. The disease severity index (DSI was calculated using the following formula:

$$\mathrm{DSI}(\%)=(\frac{\Sigma ab}{N\cdot K})\times 100$$

where

∑*ab* = sum of the symptomatic leaves with their corresponding scale
*N* = total leaves
*K* = highest score scale

Score data recorded from week 3 to week 7 for each fungal mutant. The mean of the DSI at different weeks after inoculation was evaluated to assess the infection ability of the fungal mutants. DSI data was subjected to ANOVA, and DSI between wild-type and mutants, and between mutant and mutant infections at each week were subjected to Tukey’s multiple comparison test. Statistical analyses were conducted using GraphPad Prism version 11.0.0.

h. In silico characterisation of the selected hydrophobin

To further characterise the selected hydrophobin gene, several in silico analyses were conducted using bioinformatic tools. Signal peptide prediction was carried out using SignalP 6.0 (https://dtu.biolib.com/SignalP-6) [52], DeepSig (https://busca.biocomp.unibo.it/deepsig/) [53] and PHOBIUS (https://phobius.sbc.su.se/) [54], while ProtScale (https://web.expasy.org/protscale/) was used to assess various physicochemical properties based on the amino acid sequence. InterPro (https://www.ebi.ac.uk/interpro/) was used to identify conserved motifs and domains. In addition, a phylogenetic tree was constructed using Bayesian inference via Mr Bayes v.3.2.7 [55], utilising 129 hydrophobin sequences spanning Class I, Class II, and Class III hydrophobins. Amarkov chain Monte Carlo (MCMC) algorithm was used to generate Bayesian inference of phylogenetic trees using the mixed amino acid substitution model with a gamma-distributed rate variation across sites and a proportion of invariable sites with 8 gamma categories. Analyses of two parallel MCMC runs with four chains (containing 3 heated chains at temperature of 0.2, and 1 unheated chain) were run for 5,000,000 generations and sampled every 100 generations. The first 25% tress were discarded as the burn-in phase of the analysis. The phylogenetic tree was visualised with ITOL (http://itol.embl.de).

## 3. Results

### 3.1. Completion of the pGreen0179Glcas9

**Figure 3:**
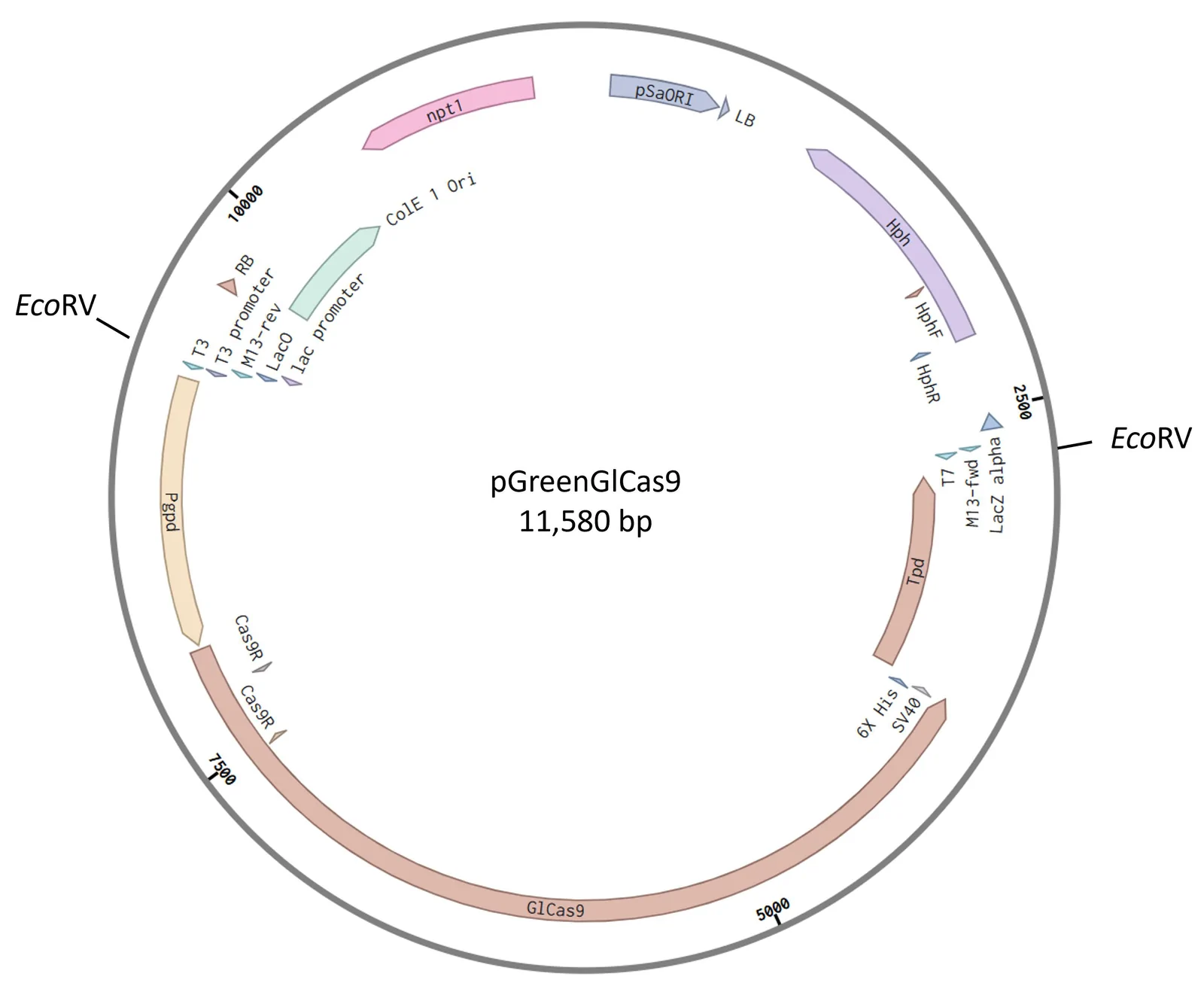
Components of the recombinant vector pGreen0179Glcas9. The Glcas9 cassette (6450 bp), incorporating the GPD promoter and PDC terminator with added restriction sites for cloning, was inserted into the pGreen0179 backbone to yield a construct of ~11.5 kb. The vector carries a *hph* cassette as a selectable marker, conferring hygromycin B resistance to transformed cells. The vector has been illustrated using Benchling (https://benchling.com/).

The Glcas9 cassette, driven by P*_gpd_* and terminated by T*_pd_*, was successfully synthesised. With the addition of restriction enzyme sites to facilitate cloning, the final size of the cassette measured 6450 bp in length. It was then cloned into the pGreen0179 vector, resulting in the recombinant construct pGreen0179Glcas9, with an estimated size of approximately 11.5 kb. The detailed composition and structure of the vector are depicted in Figure 3. The vector backbone includes a hygromycin phosphotransferase (*hph*) gene, which functions as a selectable marker. Upon expression, the *hph* gene encodes an enzyme that phosphorylates and inactivates hygromycin B, thereby conferring resistance. As a result, only cells harbouring the *hph* gene are able to grow in the presence of hygromycin B, while non-transformed cells will be killed.

### 3.2. Designing and Validation of the Potential sgRNAs for Targeted Genes

The single guide RNA (sgRNA) is a key component of the CRISPR/Cas9 genome editing system. It directs the Cas9 endonuclease to a specific DNA sequence by base pairing with the target site, enabling precise DNA cleavage. The accuracy and efficiency of CRISPR editing are highly dependent on the design of the sgRNA. A well-designed sgRNA not only improves target specificity but also reduces the risk of off-target effects [44]. 

#### 3.2.1. Selection of sgRNAs for *hyd* Genes

Using BLASTN analysis with the reference *pyrG* gene sequence from *L. edodes*, corresponding *pyrG* sequences were successfully identified in both the genomic and transcriptomic databases, showing similarity scores of 57.7% and 64.3%, respectively. A comparison of the *pyrG* gene between *G. boninense* strain PER71 and G3 revealed a high sequence similarity of 97.45%. The full-length sequence of the *G. boninense* PER71 *pyrG* gene is included in Figure S2. Given that strain PER71 was used in this study, its *pyrG* transcript sequence served as the input for sgRNA prediction using CCTop—CRISPR/Cas9 Target Online Predictor. Three candidate sgRNAs targeting the first and second exons of *pyrG*, each with CRISPRater scores above 0.70, and showed not more than four base-pair matches to the non-target genes were selected for gene editing-based functional analysis. The megaprimer sequences used for sgRNA synthesis, along with the reverse primer (Rvs.sguniversal), are presented in Table 2. Alignment of the gene, transcript sequences, and sgRNA targets are detailed in Figure S2. One selected sgRNA showed high GC content, which was 75%, however, still selected due to other factors: high CRISPRater score, low seed sequence similarity with another region in the genome and located within the first two exons.

#### 3.2.2. Selection of sgRNAs for *hyd* Gene

**Figure 4:**
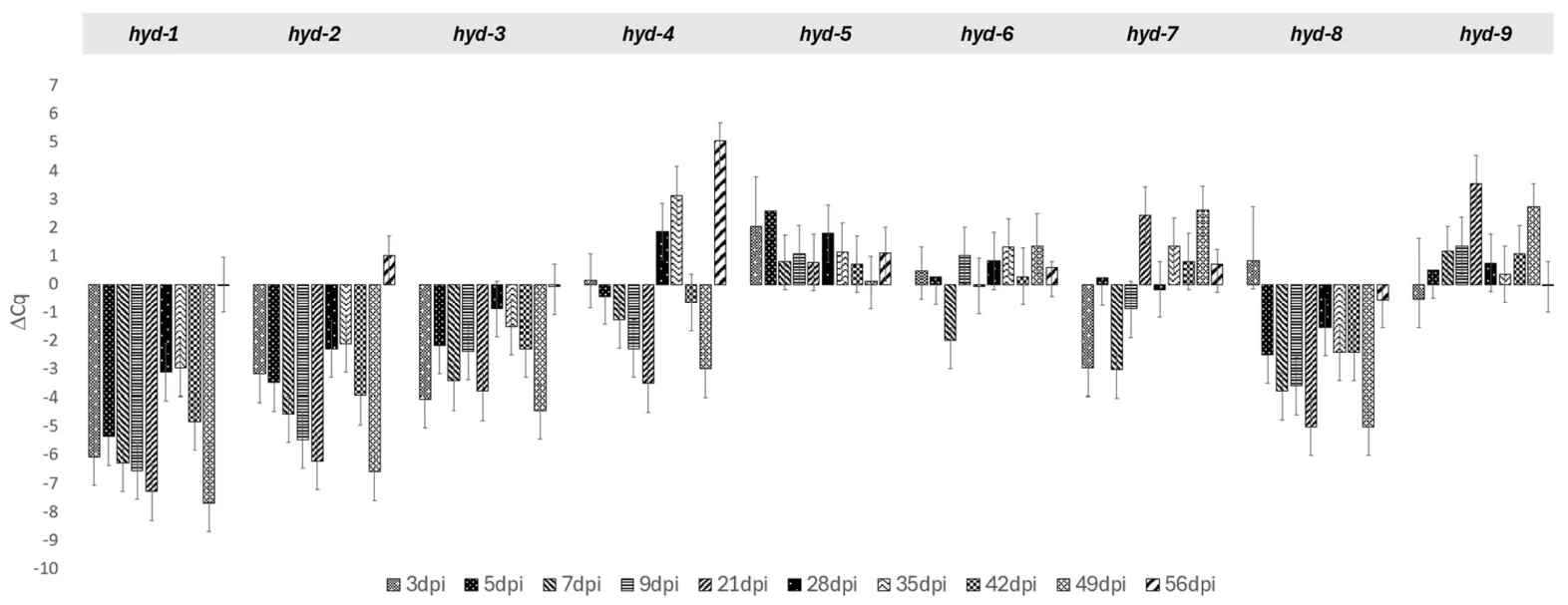
Relative expression profiles of *hyd* genes (*hyd-1* to *hyd-9*) during infection in oil palm tissue culture over a 56-day period. Gene expression was measured using quantitative PCR and normalised against three housekeeping genes. ΔC_q_ values were calculated to represent relative transcript abundance, with more negative values indicating higher gene expression. Multiple time points (3, 5, 7, 9, 21, 28, 35, 42, 49, and 56 days post-inoculation) were assessed for each gene. Error bars represent standard error (SE) from four biological replicates (Table S3).

In this study, a candidate effector protein, hydrophobin, which may contribute to the pathogenicity of *G. boninense* was selected based on preliminary gene expression profiling (Figure 4). Quantitative PCR (qPCR) analysis was conducted using infected roots of one-month-old oil palm ramets collected at various infection time points. The relative expression profiles of hydrophobin genes (*hyd-1* to *hyd-9*) were monitored over an eight-week (56-day) infection period using qPCR (Figure 4). The ΔC_q_ values were calculated by normalising against three reference housekeeping genes. This approach ensured accurate assessment of relative transcript levels across time points. Based on the gene expression profile, *hyd-1*, *hyd-2*, *hyd-3*, and *hyd-8* exhibited more negative ΔC_q_ values, particularly between 3 and 9 dpi, indicating higher expression during the early stages of infection. These genes also showed sustained upregulation throughout the infection period, suggesting a continuous role in the host–pathogen interaction. In contrast, *hyd-4*, *hyd-5*, *hyd-6*, *hyd-7*, and *hyd-9* showed relatively higher ΔC_q_ values, signifying lower expression overall. These findings potentially imply that *hyd-1*, *hyd-2*, *hyd-3* and *hyd-8* may be important for pathogenesis, particularly during both early and sustained phases of infection.

The fluctuation in the different *hyd* genes’ expression may be attributed to several factors, i.e., (i) stage-specific functions of certain hydrophobins are crucial during the early phases of infection, assisting in the adhesion to host or substrate surfaces and facilitating penetration into host tissues [43,56]. (ii) Host environmental changes has shifts in host conditions during infection, such as the production of reactive oxygen species (ROS), pH variations, and altered nutrient availability, can significantly influence gene regulation [57,58]. (iii) Evasion of host immune response in downregulation of *hyd-2* may serve as a strategy to avoid detection by the host immune system, similar to the role of RodA in *Aspergillus fumigatus* [59,60]. (iv) Differential regulation mechanisms during expression of hydrophobin genes may be governed by infection-specific transcription factors or signaling pathways and may be coordinated with other hydrophobins to perform distinct functions during host–pathogen interactions [56,61,62].

Based on this preliminary analysis, we attempted functional characterisation of the two hydrophobin genes with the highest expression during different infection stages, i.e., *hyd-1* and *hyd-2*. However, CRISPR/Cas9 gene editing experiments for *hyd-1* yielded only base substitution mutations (*hyd-1*) in the first trial and further experiments were not repeated with different sgRNAs (unpublished data). Hence, the *hyd-2* gene was selected for further functional characterisation and pathogenicity tests.

Following the selection of *hyd-2* for functional analysis, three candidate sgRNAs targeting the first and second exons were identified using CCTop—CRISPR/Cas9 Target Online Predictor. Each sgRNA had a CRISPRater score above 0.75 and shared no more than four base pairs of similarity with non-target genes, ensuring high target specificity. The selection of the sgRNA for *hyd-2* gene also involved the potential sgRNAs sequences with high GC content, 66.67% and 75% for Fwd.sghyd2-1 and Fwd.sghyd2-3, respectively (Table 3). This is due to the high sequence similarity of the target *hyd-2* gene with other hydrophobin genes present in the genome, especially at the region suitable for designing the sgRNAs, hence limiting the choice of potential sgRNAs. The megaprimers for sgRNA synthesis are listed in Table 3. The reverse primer used was similar as provided in Table 2. The alignment of the gene, corresponding transcript sequences, and sgRNA target sites is provided in Figure S5.

#### 3.2.3. Preparation of the sgRNAs Using PCR Approach

**Figure 5:**
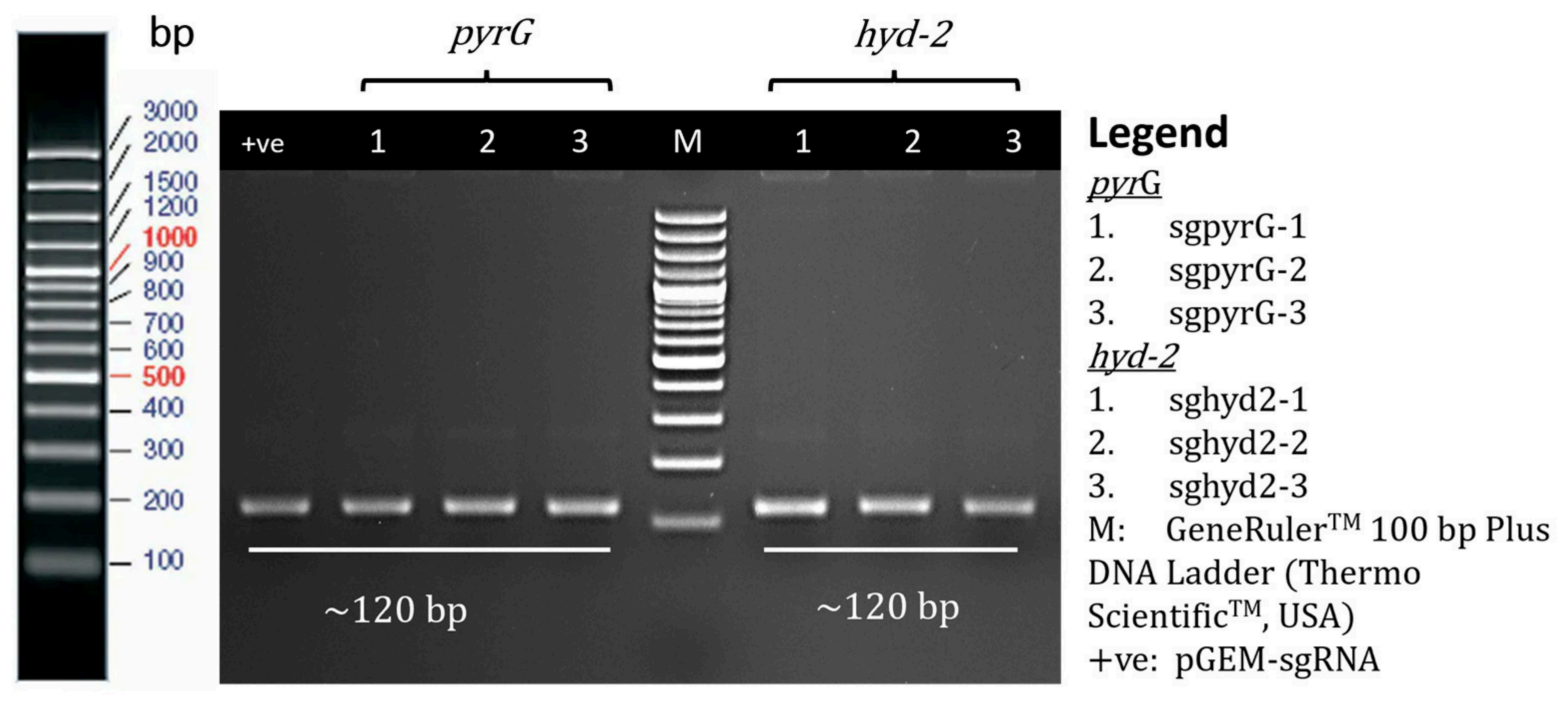
PCR amplification of full-length sgRNAs showing ~120 bp amplicons. The PCR products were electrophoresed on a 1.5% agarose gel in 1× TAE buffer. Lanes 1–3 represent different sgRNAs as indicated in the legend. Clear bands at ~120 bp confirm successful amplification of the expected sgRNA fragments. Faint bands at ~250 bp are possibly nicked template plasmid or unspecific binding.

There are several methods for preparing sgRNA targeting a specific gene. The first approach involves using two overlapping DNA oligonucleotides: the first oligo contains the T7 promoter and a 20-nucleotide Cas9 target sequence, while the second includes the sgRNA backbone, which encompasses the scaffold tracrRNA (trans-activating CRISPR RNA). These oligos can be combined using a PCR-based method [63]. The second method involves annealing oligonucleotides using a PCR thermal cycler. In this approach, the sgRNA sequence is designed with overhangs at both ends to facilitate annealing with a linearised vector, followed by ligation using T4 DNA ligase [64]. The third method, as employed in this project, involves designing a 59–60-nucleotide megaprimer that includes the T7 promoter, the 20-nucleotide sgRNA targeting sequence, and a partial scaffold tracrRNA sequence. The complete sgRNA will be obtained through PCR analysis, employing the megaprimer, and the reverse universal primer as Table 2, and plasmid containing the full cassette of sgRNA, comprising T7 promoter, 20-nucleotide sgRNA of the targeted gene, and ~80 nt of scaffold tracrRNA (trans-activating CRISPR RNA) sequence as the template DNA. The scaffold tracrRNA is a crucial component of the CRISPR/Cas9 system, forming part of the sgRNA that binds and stabilises the Cas9 protein, enabling its correct conformation for DNA cleavage. It directly interacts with Cas9’s recognition domains to form the active editing complex [65,66,67]. The profile of the amplified sgRNAs for both *pyrG* and *hyd-2* is depicted in Figure 5. The amplified sgRNAs, were purified and successfully transcribed using the Guide-it™ sgRNA in vitro Transcription and Screening System kit, following the manufacturer’s protocol prior to the transformation into protoplast PEG-mediated transformation analysis.

### 3.3. Validation of the Mutants

Prior to the functional analysis of the targeted genes, plasmid uptake by the protoplasts was confirmed by PCR analysis of total extracted DNA, while expression of the *Glcas9* gene in *G. boninense* was verified by RT-PCR (Figure S3).

In this project, a minimum of three sgRNAs were designed for each target gene to enhance the likelihood of successful gene editing [68,69]. Utilising multiple sgRNAs has been shown to improve editing efficiency by increasing the frequency of double-strand breaks (DSBs), enhancing disruption of key coding regions, and providing functional redundancy in cases where individual guides may be limited by chromatin accessibility or suboptimal activity. This multiplexing strategy has been demonstrated to significantly improve editing outcomes and increase the probability of achieving complete gene knockouts [68].

#### 3.3.1. *pyrG* Mutants

**Figure 6:**
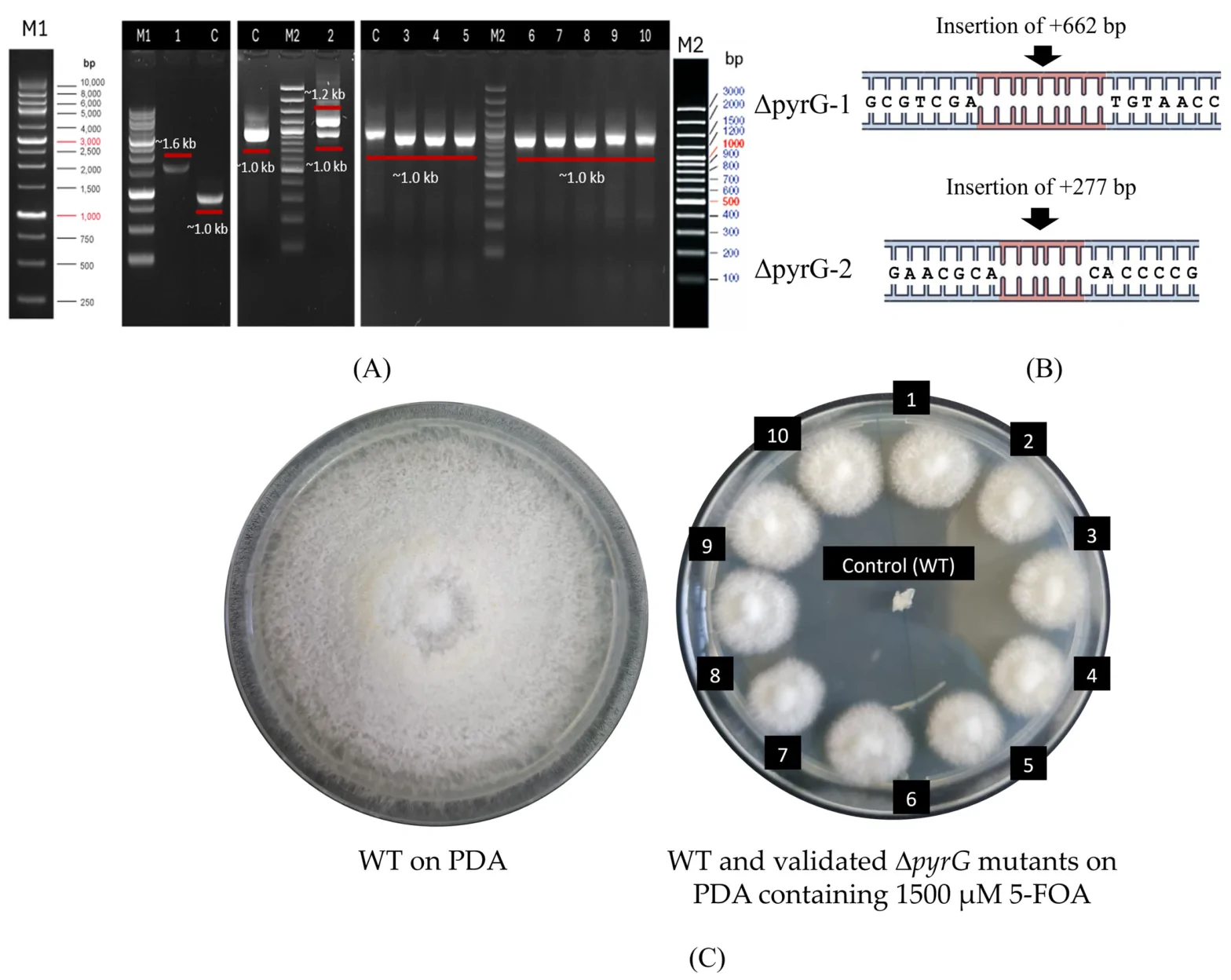
Validation of the *pyr*G mutants. (**A**) PCR amplification products analysed by agarose gel electrophoresis. Lane C: control (wild-type *pyrG*); Lanes 1–10: putative *pyrG* mutants; Lane M1: DNA size marker (1st Base 1 kb DNA Ladder, Singapore); Lane M2: DNA size marker (GeneRuler 100 bp Plus DNA Ladder, Thermo Fisher Scientific, USA). (**B**) Sequence analysis revealed insertions of +662 bp for ΔpyrG-1 and +277 bp for ΔpyrG-2 (the ~1.2 kb band). (**C**) The WT *G. boninense* PER71 exhibited normal fluffy colony morphology when grown on PDA. In contrast, the validated ΔpyrG mutants retained fluffy morphology and were able to grow on PDA supplemented with 1500 µM 5-FOA, whereas the WT failed to grow due to 5-FOA toxicity.

From the transformation analysis, 10 out of 51 putative Δ*pyrG* mutants demonstrated stable growth on PDA supplemented with 1500 µM 5-FOA, sustained through at least three generations. Subsequent PCR amplification of the *pyrG* gene from genomic DNA using a specific primer pair (Table S1) revealed that two mutants (mutants ΔpyrG-1 and ΔpyrG-1) exhibited a marked increase in product size, indicative of insertional mutations (Figure 6A). The size of the wild type (WT) *pyrG* amplicon size is ~1000 bp.

PCR amplification of the ΔpyrG-1 mutant using *pyrG*-specific primers produced a single ~1.6 kb band, whereas the ΔpyrG-2 mutant yielded two distinct bands (~1.0 kb and ~1.2 kb) (Figure 6A). Other putative mutants generated amplicons comparable in size to the WT. Cloning and sequencing results confirmed that ΔpyrG-1 carried a single ~662 bp insertion, while ΔpyrG-2 contained a heterogeneous population of *pyrG* sequences, with the largest insertion measuring 277 bp (Figure 6B and Figure S4), while others show single base deletion and single and multiple base substitution. BLASTN analysis revealed that the ΔpyrG-1 insertion shared ~94% and ~91% identity with *G. boninense* G3 linkage group 2 (LG2) and 99.61% with LG8, while the ΔpyrG-2 insertion showed >98% identity with regions in LG3 and LG4. The remaining *pyrG* mutants exhibited only minor sequence changes, which reflect typical results of CRISPR/Cas9-based editing, such as 1–2 base pair deletions in the first exon that induced frameshift mutations leading to loss of protein function, or base substitutions whose effects varied depending on their specific nature and position within the coding sequence. All the validated mutants exhibited normal growth, comparable to the WT on PDA (Figure 6C).

Base substitution rates with CRISPR/Cas9 are generally low because the system primarily introduces DSBs, which are typically repaired through non-homologous end joining (NHEJ) that favours insertions and deletions (indels) over single-base substitutions [70,71,72]. However, sequencing of randomly selected clones carrying cloned *pyrG* PCR products derived from the mutant strains revealed a higher frequency of base substitutions than indels. Among the 46 sequenced clones, 28% retained the WT sequence, 50% contained base substitutions, and 22% harboured insertion/deletion mutations.

Nevertheless, sequencing and cloning analyses reveal that, with the exception of the ΔpyrG-1 mutant, in which 100% of sequencing results showed the ~662 bp insertion, most other mutants still retain a substantial proportion of WT sequences. The heterogeneous sequencing profiles likely reflect incomplete editing of genetically distinct nuclei within the dikaryotic mycelium and/or continued DNA repair following Cas9-induced cleavage, rather than uniform editing of all nuclear genomes. Similar asymmetric editing of the two nuclei has been reported in dikaryotic *G. lucidum* following CRISPR/Cas9-mediated genome editing [39].

#### 3.3.2. Screening and Validation of *G. boninense* *hyd-2* Mutants

**Figure 7:**
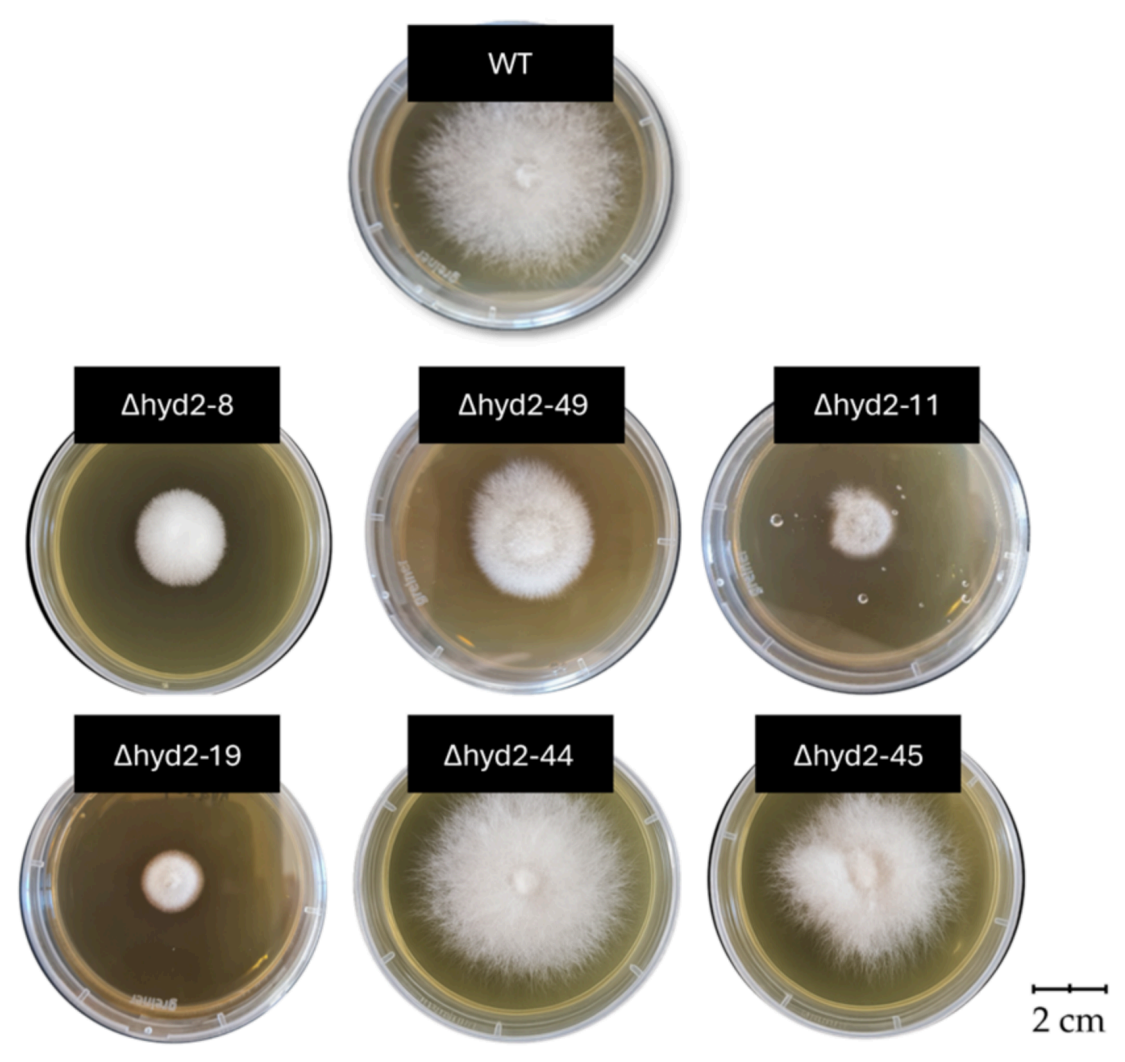
Colony or mycelia morphology of *G. boninense* (WT) and selected Δ*hyd-2* mutants grown on half-strength PDA. WT shows full radial growth with fluffy hyphae, while indel mutants such as Δhyd2-8 and Δhyd2-10, Δhyd2-11 and Δhyd2-19 exhibit reduced growth and compact colonies, whereas base substitution mutants (Δhyd2-44, Δhyd2-45) display growth patterns similar to WT.

A total of 12 putative *G. boninense* ∆*hyd-2* mutants exhibiting the Hyg^R^ phenotype were consistently observed across at least three generations. Cloning and sequencing analyses revealed the mixed presence of the WT and edited *hyd-2* sequences in each of the putative ∆*hyd-2* mutants, similar to what was observed in the ∆*pyrG* mutants. To further validate these ∆*hyd-2* mutants, sequencing was also performed using cDNA generated from RNA extracted from in vitro *Ganoderma*-infected roots at 7 dpi. Some of the edited genes underwent extensive sequence alterations, resulting in markedly reduced similarity to the WT sequence. Such extensive mutations may have also altered intron recognition, as observed in Δhyd2-11 (Figure S6). The predominant types of mutations were base substitutions, followed by small insertions and deletions (indels). The morphology of the validated ∆*hyd-2* mutants is shown in Figure 7. The ∆*hyd-2* mutants carrying indel-induced frameshift mutations displayed impaired mycelial growth compared to the WT, as seen in Δhyd2-8, Δhyd2-11, Δhyd2-19, and Δhyd2-49. In contrast, mutants with base substitutions, such as Δhyd2-44 and Δhyd2-45, exhibited growth patterns that closely resembled the WT. Multiple sequence alignment of cloned *hyd-2* sequences of Δhyd2-44, Δhyd2-45, Δhyd2-15 and Δhyd2-16 showed only single base substitutions throughout the *hyd-2* gene (Figure S6). A second multiple sequence alignment of cloned *hyd-2* sequences of the indel mutants Δhyd2-8, Δhyd2-11, Δhyd2-19, Δhyd2-49 showed varying types of insertions and deletions. Δhyd2-11 possessed multiple deletions (of 11 bp, 5 bp and two other single base deletions) and insertion (12 bp insertion), while Δhyd2-8 possessed a single base deletion, whereas Δhyd2-19 and Δhyd2-49 possessed multiple base deletions (a 4 bp deletion and 2 bp deletion, respectively). All four indel mutants also showed multiple base substitutions throughout the *hyd-2* gene (Figure S6). These rampant mutations have resulted in a loss-of-function of the *hyd-2* gene, as observed in their phenotypes.

### 3.4. Physical Properties and Pathogenicity Assessment via Ganoderma In Vitro Infection Assay

As shown in Figure 7, two *G. boninense* ∆*hyd-2* mutants, Δhyd2-11 and Δhyd2-19, which exhibited markedly reduced radial growth compared to the WT strain and other ∆*hyd-2* mutants, were selected for preliminary pathogenicity assessment using an in vitro *Ganoderma*-infection assay. Whereas the WT strain fully colonised the plate within 6–7 days, both mutants displayed restricted growth, covering less than half of the plate. Moreover, the mutant colonies developed shorter, denser, and less aerial hyphae, lacking the fluffy appearance characteristic of the WT.

**Figure 8:**
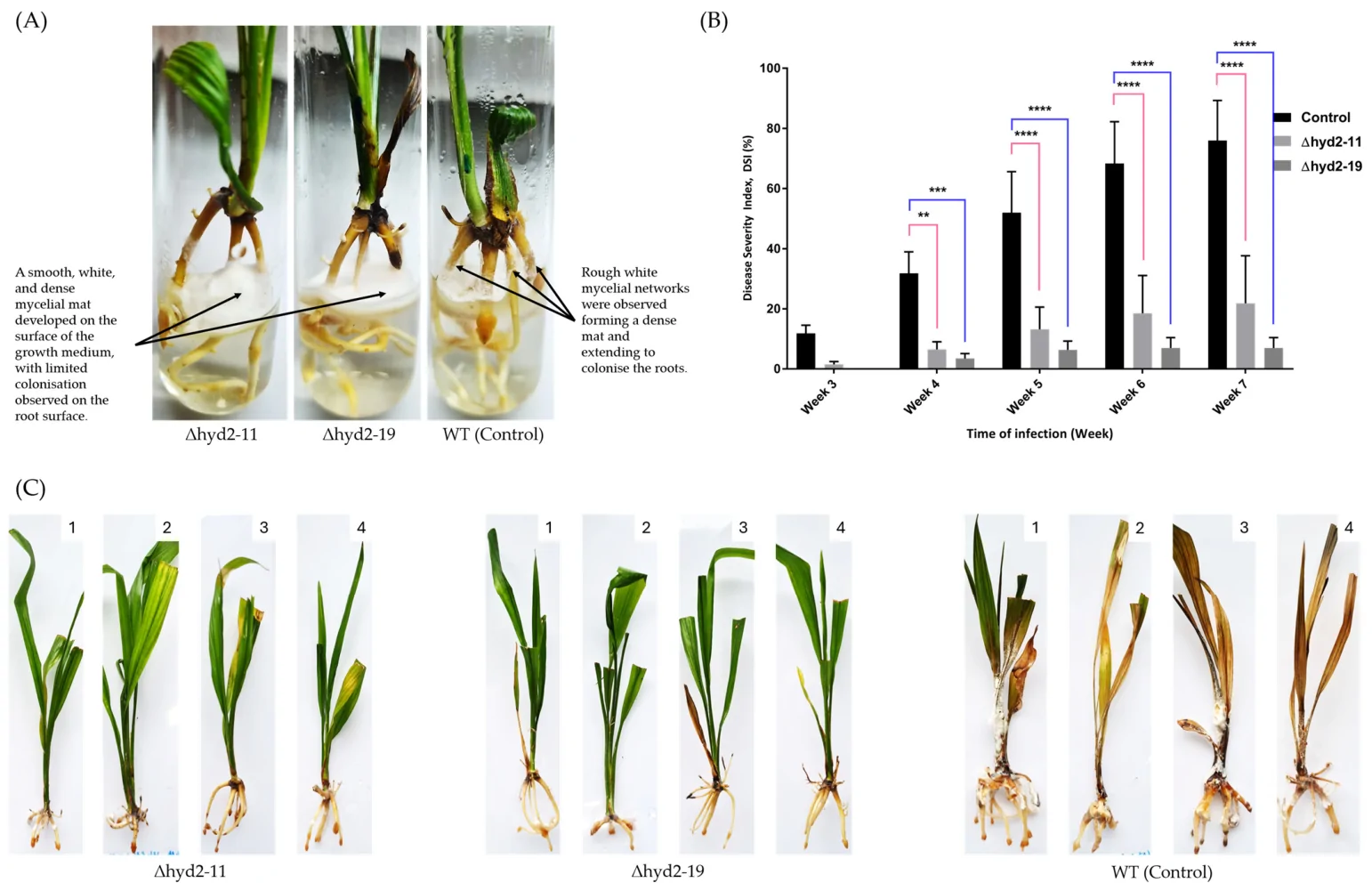
Morphology of Δ*hyd-2* mutants in the in vitro *Ganoderma* infection assay and their DSI scores compared with the WT *G. boninense* PER71 from week 3 to week 7 post-inoculation. (**A**) Mycelial mat formation was observed at 7 dpi. The Δ*hyd-2* mutants developed a smoother mycelial mat with restricted colonisation at the root surface, whereas the WT produced a rougher mat with extensive mycelial colonisation. (**B**) DSI values were recorded weekly (W3–W7) following infection with WT, demonstrating attenuated virulence in host tissues (** *p* < 0.01; *** *p* < 0.001; **** *p* < 0.0001). Error bars represent standard deviation from six biological replicates. (**C**) At 7 wpi, WT *G. boninense* infection caused severe foliar necrosis and root decay, whereas both of the Δ*hyd-2* mutants produced only mild yellow leaf spotting, with infected roots remaining intact and retaining a sturdy texture. Numbers 1–4 indicate representative infected ramets for each treatment (WT and Δ*hyd-2 *mutants).

In the preliminary in vitro *Ganoderma*-infection assay, mycelial mat formation was observed at 7 dpi. The ∆*hyd-2* mutants developed smoother mats with restricted colonisation at the root surface, in contrast to the WT, which produced rough mats with extensive colonisation (Figure 8A). At 7 week-post inoculation (wpi), root structure observations revealed that mutant-infected ramets remained sturdy and intact, whereas WT-infection resulted in severe root decay, leaving the roots fragile and easily disintegrated upon physical contact. Consistently, foliar symptoms in mutant-infected plants were mild, showing only localised yellow spots, while WT infection caused extensive necrosis, with leaves turning brown and ultimately leading to plant death (Figure 8B).

The infection capability of Δhyd2-11 and Δhyd2-19 was further evaluated over a 5-week observation period, with DSI scores recorded weekly from week 3 to week 7 post-inoculation. Compared to the WT (control), both Δhyd2-11 and Δhyd2-19 exhibited a marked reduction in disease severity, with overall DSI values lowered by ~72% and ~91%, respectively (Figure 8C). Two-way repeated measures ANOVA revealed significant effects of genotype (*p* = 0.0019) and time (*p* < 0.0001) on DSI. A significant genotype × time interaction was also detected (*p* < 0.0001), indicating that disease progression over time differed significantly among the WT, Δhyd2-11, and Δhyd2-19 strains (Table S2). These findings demonstrate that disruption of *hyd-2* not only reduced the overall severity of infection but also altered the temporal progression of disease development during the experimental period.

The substantial reduction in DSI score observed in ∆*hyd-2* mutants in this preliminary analysis, ~72% in Δhyd2-11 and ~91% in Δhyd2-19, which supported the role of Hyd-2 as one of the potential key virulence factors in *G. boninense*. The consistent attenuation of infection across multiple time points indicates that disruption of *hyd-2* significantly impairs the pathogen’s ability to colonise host tissues, making it a compelling target and candidate for antifungal development. Frameshift mutations caused by indels within conserved domains of Hyd-2, such as the hydrophobic patch and cysteine-rich motifs, likely compromise its structural assembly and functional activity. Since hydrophobins are directly involved in surface interactions, host penetration, and masking of fungal cells from host recognition, loss of function would render the pathogen unable to properly execute its role in pathogenesis. Moreover, the strong upregulation of *hyd-2* in the WT during disease progression (Figure 4), suggests a potential direct link between *hyd-2* expression and virulence. This pattern indicates that *hyd-2* is functionally required not only for the early establishment of infection but also for sustaining colonisation and driving disease development at later stages. The impaired virulence of the mutants can thus be interpreted as a direct consequence of their inability to execute these *hyd-2*–dependent pathogenic functions, underscoring the broader role of hydrophobins in mediating host invasion and colonisation [73].

### 3.5. Bioinformatic Characterisation of the Fungal Effector Hyd-2

The gene is 343 bp in length and encodes a protein comprising 110 amino acids. BLAST analysis of the deduced amino acid sequence against the UniProt database revealed sequence similarities of 89.1%, 82.7%, 79.1%, 73.0%, and 72.1% with hydrophobin proteins from *Ganoderma sinense* ZZ0214-1, *Trametes pubescens*, *Pycnoporus cinnabarinus*, *Trametes coccinea*, and *Lentinus brumalis*, respectively. Signal peptide analysis using SignalP 6.0 [52] and DeepSig [53] predicted a cleavage site between residues 19 and 20, whereas Phobius [54] suggested cleavage between residues 17 and 18. Such minor discrepancies reflect the differing modelling frameworks—deep neural networks vs. hidden Markov models—and their weighting of sequence motifs such as the (−3, −1) rule [74]. In practice, signal peptidase itself can tolerate slight positional flexibility in cleavage [75,76]. Nevertheless, all predictors consistently indicate the presence of a N-terminal signal peptide and its secretion via the classical ER–Golgi pathway. The predicted signal peptide comprises three distinct regions—the N-, H-, and C-regions—responsible for membrane interaction, anchoring, and cleavage, respectively. Together, these regions ensure proper protein targeting and translocation into the designated cellular compartments [77]. The Kyte–Doolittle hydropathy profile of the Hyd-2 protein revealed a characteristic alternating pattern of hydrophobic and hydrophilic regions, with a prominent hydrophobic peak around residues 35–40. This peak is consistent with the class I hydrophobin signature associated with surface-assembling domains, while hydrophilic troughs near residues 20, 60, and 75 likely correspond to loop or solvent-exposed regions—reflecting its amphipathic nature [78].

**Figure 9:**
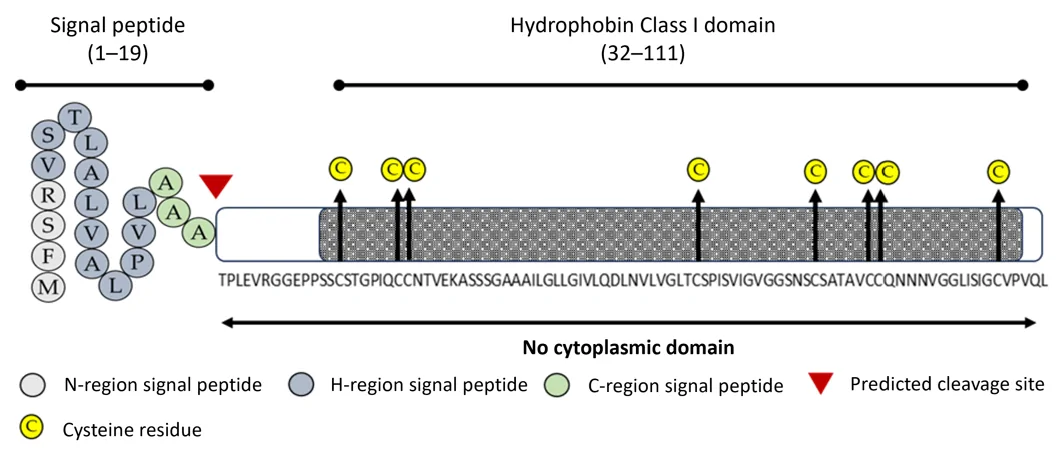
Predicted structure of the Hyd-2 protein. A signal peptide was identified at the N-terminus (residues 1–19), comprising the characteristic N-, H-, and C-regions. The N-region facilitates targeting of the nascent chain to the membrane, the hydrophobic H-region enables insertion into the lipid bilayer, and the C-region contains the recognition site for signal peptidase cleavage, releasing the mature protein. The presence of eight conserved cysteine residues further supports the classification of Hyd-2 as a Class I hydrophobin.

InterPro domain analysis (IPR001338/PF01185) classifies the protein as a class I hydrophobin, a family of small, cysteine-rich proteins that self-assemble at hydrophilic–hydrophobic interfaces to form amphipathic films [47,79,80]. The annotated hydrophobin class I domain, spanning approximately residues 20 to 110, contains eight conserved cysteine residues that form four disulfide bonds—structural features essential for rodlet formation and protein stability [57,81]. All detailed analyses of the hydropathy profile and InterPro domain predictions are provided in Figure S7, while the protein structure illustration of Hyd-2 is shown in Figure 9.

**Figure 10:**
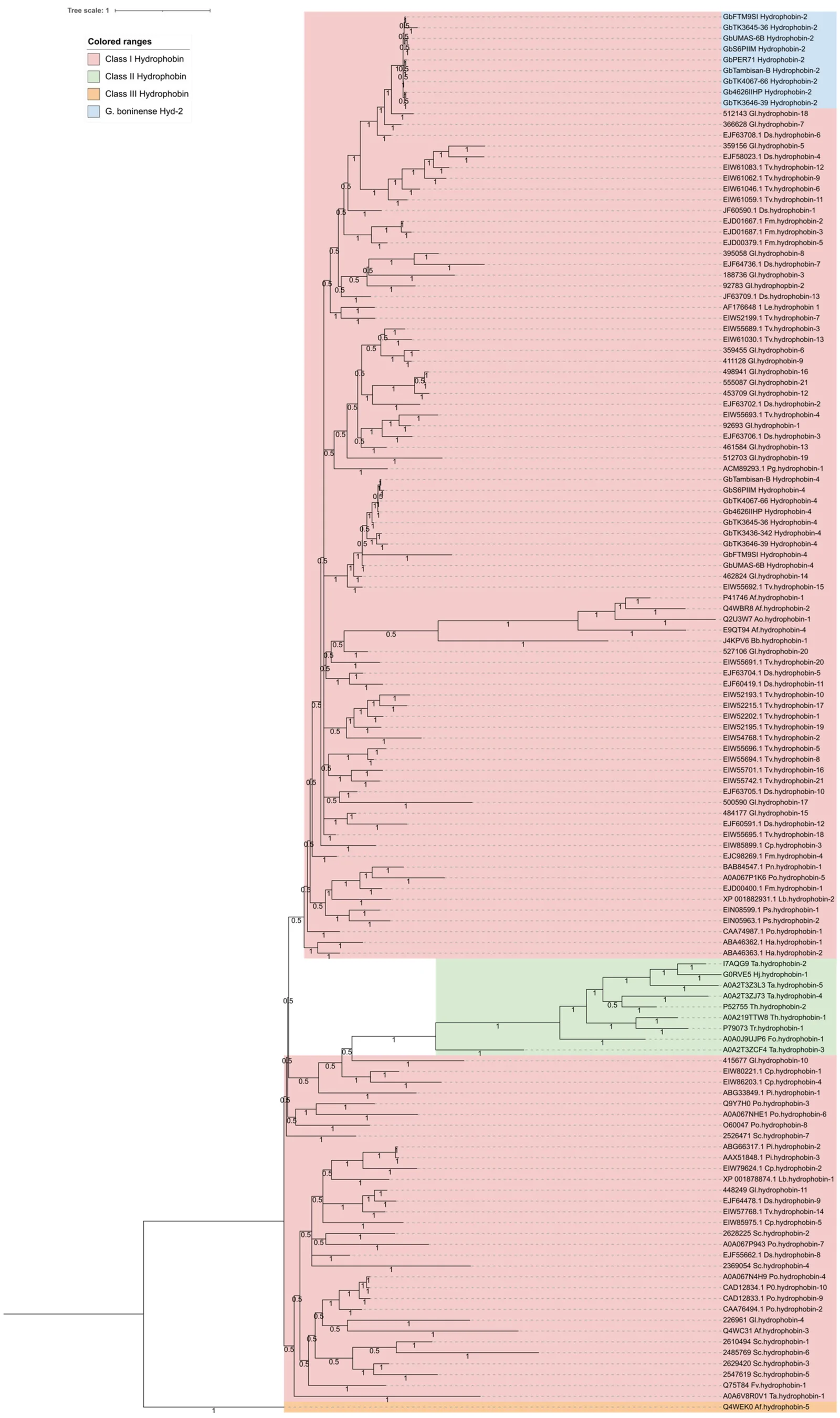
Bayesian phylogenetic tree of the hydrophobin proteins showed that the Hyd-2 amino acid sequences from different *G. boninense* strains (highlighted in blue) clustered together with *G. lucidum* and *D. squalens*, reflecting their close evolutionary relationship within the order *Polyporales* (phylum Basidiomycota). Node values indicate posterior probabilities.

Phylogenetic analysis shows that hydrophobin-2 from *G. boninense* clusters with orthologous hydrophobin sequences from *G. lucidum*, *Dichomitus squalens*, and *Trametes versicolor*, forming a conserved Polyporales clade (Figure 10). This clustering reflects the evolutionary conservation of a core hydrophobin gene. Despite this conservation, functional deletion of *hyd-2* in *G. boninense* in this study resulted in an approximately ~91% reduction in pathogenicity, indicating that this conserved hydrophobin is essential for effective host colonisation rather than being functionally redundant. The details of the hydrophobin sequences used in the construction of phylogenetic tree analysis are provided in Table S4.

## 4. Discussion

### 4.1. Establishing the Machinery for the CRISPR/Cas9 System in G. boninense

The CRISPR/Cas9 system has emerged as a powerful and versatile tool for functional genomics in a wide range of organisms, including fungi, plants, and animals [82,83,84]. By generating targeted double-strand breaks, it enables precise gene disruption, replacement, or editing, thereby accelerating functional studies of specific genes [85]. Compared with traditional mutagenesis approaches, CRISPR/Cas9 provides higher efficiency, greater specificity, and the capacity for multiplex editing [85]. For complex organisms such as *G. boninense*, where molecular tools remain limited [16], the system offers significant potential for dissecting gene function, validating candidate host’s resistance genes (R), and investigating host–pathogen interactions at the molecular level.

Despite these advantages, functional analysis in *G. boninense* remains in infancy. Only a few studies have reported progress, largely due to the technical challenges imposed by its complex genome and difficulties in stable transformation [16]. Recent advances in RNA interference (RNAi) have demonstrated the feasibility of gene silencing in *G. boninense* [23], yet most applications have been confined to transient expression systems, limiting long-term stability and reproducibility. These constraints highlight the need for a robust and reliable genome-editing platform—such as CRISPR/Cas9—to enable deeper functional genomics studies in this organism.

To address this, the *cas9* sequence was codon-harmonised according to the codon usage bias of *G. lucidum*. Codon harmonisation was employed to enhance translation efficiency and ensure proper folding of the Cas9 protein in the fungal host. Because organisms vary in their codon usage bias, reflecting differences in tRNA abundance, direct expression of *cas9* from its native source (*Streptococcus pyogenes*) could result in inefficient translation, ribosomal stalling, or reduced protein yield [86]. Using *G. lucidum* as the reference species was a deliberate choice, as its genome has been sequenced and annotated across multiple independent studies, providing one of the most comprehensive genomic resources available within the genus. This ensures greater accuracy in predicting codon preferences and enables better alignment of *cas9* with the host’s translational machinery. Although *G. boninense* is the species of greatest relevance due to its pathogenicity, the relatively incomplete genomic resources available for this fungus made *G. lucidum* the more reliable reference. This approach therefore serves as a practical compromise until high-quality codon usage datasets for *G. boninense* become available.

Building on this, the *Glcas9* cassette was successfully synthesised, driven by the constitutive P*_gpd_* promoter and terminated by T*_pd_*, representing an important milestone toward developing a functional genome-editing system for *G. boninense*. The construct includes the *hph* selectable marker, which confers resistance to hygromycin B and provides a reliable means of distinguishing transformed from non-transformed cells. Given the widespread use and proven reliability of hygromycin resistance in fungal transformation systems, incorporation of the *hph* cassette is expected to improve selection efficiency and minimise background growth [87,88]. The codon-optimised *cas9* was confirmed to be expressed in *G. boninense*, as validated by RT-PCR and sequencing analysis. The use of a codon-optimised *cas9* is consistent with previous CRISPR/Cas9 studies in fungi such as *Aspergillus nidulans*, *Neurospora crassa*, *Pyricularia oryzae* and other eukaryotes, where optimisation of the bacterial *cas9* coding sequence to match host codon usage has improved transgene expression and genome-editing efficiency [89,90,91].

The design of the sgRNA is a critical determinant of the efficiency and specificity of the CRISPR/Cas9 system. In the present study, the editing efficiency of each individual sgRNA was not evaluated because all three sgRNAs were co-transformed in a single reaction to maximise the likelihood of generating gene-edited mutants. Nevertheless, evaluating each sgRNA independently is recommended to determine its contribution to the frequency and type of genome-editing events. The efficiency of CRISPR/Cas9-mediated targeting is strongly influenced by the nucleotide composition and structural characteristics of the sgRNA, which guide Cas9 to the target locus with high specificity. Consistent with previous reports, an optimal sgRNA typically consists of a 20-nucleotide guide sequence with a GC content of 40–60%, particularly within the seed region, to promote stable target binding while minimising mismatches [92]. Furthermore, specific sequence features, such as guanine enrichment proximal to the protospacer adjacent motif (PAM), have been associated with enhanced Cas9 binding and cleavage efficiency [93,94,95].

Taken together, these considerations highlight that sgRNA design extends beyond simple sequence selection and requires a nuanced understanding of both compositional and structural parameters. For genome-editing applications in complex organisms such as *G. boninense*, where genetic resources and transformation systems are still developing, careful optimisation of sgRNA design is likely to be a decisive factor for achieving reliable editing outcomes. Incorporating computational prediction tools and experimental validation will therefore be essential for refining sgRNA selection [96,97] and ensuring the success of CRISPR/Cas9–mediated functional studies in this genus.

### 4.2. Development of a pyrG Mutant as a Proof-of-Concept

The *pyrG* gene has frequently been employed as a selectable marker in proof-of-concept genome-editing studies, owing to the ease with which mutants can be identified through simple nutritional selection and 5-FOA counter-selection. This dual selection strategy makes *pyrG* particularly convenient for rapidly distinguishing edited strains, and it is therefore widely used in fungi to validate transformation efficiency and genome-editing systems before applying more complex marker strategies [45,98].

The successful isolation of 10 stable *pyrG* mutants from 51 transformants demonstrates the efficiency of 5-FOA selection in identifying *pyrG*-disrupted strains in *G. boninense*. Nevertheless, the overall transformation frequency was relatively low. This may be partly attributed to the large size of the *cas9* expression plasmid (approximately 11.5 kb), which is generally associated with reduced transformation efficiency due to less efficient DNA uptake during PEG-mediated protoplast transformation [99]. In addition, the limited number of positive mutants may also be attributed to the high GC content of the *pyrG* sgRNAs, which can reduce CRISPR/Cas9 editing efficiency by promoting stable secondary structure formation that interferes with Cas9 binding and target recognition. Furthermore, highly stable sgRNA–DNA duplexes may impede efficient Cas9 cleavage, thereby reducing the frequency of successful genome-editing events [68,100]. PCR and sequencing revealed two major categories of mutations: large insertion events in ΔpyrG-1 and ΔpyrG-2, and smaller indels or base substitutions in the remaining mutants.

The results observed in mutants ΔpyrG-1 (exhibiting a single 662 bp insertion) and ΔpyrG-2 (two distinct amplicons of ~1.0 kb and ~1.2 kb, with the ~1.2 kb amplicon showing a 277 bp insertion) are atypical CRISPR/Cas9-based editing results. Sequencing and BLASTN analysis showed that these insertions originated from regions within the *G. boninense* genome, pointing to internal genomic rearrangements. These unusual findings have also been reported, where CRISPR/Cas9-editing resulted in unintended on-target mutations beyond small insertions and deletions [101]. They found that CRISPR/Cas9 frequently generated large deletions (>250 bp to over 6 kb), large insertions and complex genomic rearrangement at the target locus. They postulated that many of these results were not detected by the conventional short-range PCR detection methods because the mutations extend beyond the amplified region. Further analysis of the mechanisms underlying large deletions generated by CRISPR/Cas9-mediated genome editing was provided in an additional study [102]. By analysing deletion junctions across multiple target sites, they found that large deletions frequently contained microhomologous sequences at their breakpoints, indicating that microhomology-mediated end joining (MMEJ) also plays a role in repairing Cas9-induced DNA double-strand breaks [102]. Additional study also have reported that large genomic rearrangements, including large insertions, deletions, inversions, and duplications, can arise following CRISPR/Cas9-mediated genome editing [103]. These unintended on-target rearrangements, many of which occur independently of homologous recombination, are postulated to be rampant but undetected by conventional short-range PCR-based genotyping, which fail to detect complex editing outcomes [103]. Hence, in this study, the entire target gene regions were amplified—either from the start to stop codon of *pyrG* or spanning the upstream to downstream regions of *hyd-2*, to comprehensively assess the full impact of CRISPR/Cas9 editing events.

The possibility of heterogeneous allelism, potentially due to incomplete editing event, with possible retention of the WT sequence, is another possible cause of the unusual results, exemplified most critically in ΔpyrG-2. A similar pattern was also observed in other Δ*pyrG* and Δ*hyd-2* mutants, where clonal sequencing indicated the presence of both mutant and wild-type alleles within the same edited line, suggesting heterogeneous editing outcomes. This likely results from asynchronous CRISPR/Cas9 activity and differential repair between the two nuclei in the dikaryotic *Ganoderma* system, where one nucleus may be edited while the other remains unmodified or undergoes distinct repair outcomes, generating mixed genotypes. This is consistent with the previous report [39], which described non-uniform editing between nuclei in CRISPR/Cas9-edited *G. lucidum*.

In contrast, the remaining mutants carried smaller indels (1–3 bp deletions) or base substitutions, primarily in the first exon of *pyrG*. These mutations likely led to frameshifts or codon disruptions sufficient to abolish gene function. Importantly, all mutants displayed normal growth on PDA, the nutrient-rich media, indicating that *pyrG* is non-essential under non-selective, uracil-supplemented conditions. With the exception of ΔpyrG-1 and ΔpyrG-2, the mutation types observed in other Δ*pyrG* mutants are consistent with general outcomes of CRISPR/Cas9-mediated editing. Studies consistently show that indels are the predominant result, typically occurring in ~60% of cases with standard Cas9 systems, while base substitutions are much less frequent, often occurring below 1–3%, and sometimes as low as 0.2–0.3% [104,105]. CRISPR/Cas9 primarily generates insertions and deletions (indels) through the error-prone non-homologous end joining (NHEJ) pathway. However, CRISPR/Cas9-induced DNA double-strand breaks can occasionally result in single-base substitutions during repair. As Cas9 only introduces the DNA break, these substitutions are generated by the cell’s DNA repair machinery. Consistent with this, a previous study demonstrated that disruption of DNA ligase IV, a key NHEJ component, markedly reduced the frequency of base substitutions, indicating that NHEJ is the predominant source of these mutations [105].

Together, these features of dikaryotic fungi provide a plausible basis for the observed variation in mutation types, including the relatively rare base substitutions. Nevertheless, base substitutions remain less understood and are often underreported due to their low frequency and the possibility of being confounded by sequencing errors [106,107,108,109,110].

Although CRISPR/Cas9 has been touted as a precise method of gene disruption, our findings herein demonstrate that CRISPR/Cas9-based gene editing in *G. boninense* is not as straightforward or efficient as typically expected, as observed by the large and unexplained insertions and deletions in our proof-of-concept experiments with *pyrG*, as well as the observed of incomplete gene editing. Overall, this study demonstrates that CRISPR/Cas9-based mutagenesis in *G. boninense* yields a spectrum of mutation outcomes influenced by fungal nuclear organisation and DNA repair pathways. However, the successful generation of the Δ*pyrG* mutants exhibiting either complete loss of WT sequences with a clear auxotrophic phenotype serves as a proof-of-concept that CRISPR/Cas9-mediated gene editing, although complicated, is both functional and effective for targeted gene disruption in *G. boninense*. These findings also highlight the importance of thorough molecular validation and genome context consideration when designing gene-editing experiments in dikaryotic fungi.

### 4.3. Functional Analysis of hyd-2 Mutations and Its Effect on Fungal Virulence and Pathogenecity

The functional characterisation of *hyd-2* in *G. boninense* provides strong evidence for its role in fungal development and pathogenicity. Two *hyd-2* knockout mutants, Δhyd2-11 and Δhyd2-19, exhibited markedly reduced radial growth and altered hyphal morphology, including denser, less aerial, and non-fluffy colonies. These phenotypic changes were accompanied by severely impaired infection capacity in preliminary in vitro assays, including reduced root colonisation, mild foliar symptoms, and a dramatic reduction in DSI scores—by ~72% and ~91%, respectively—compared to the WT strain. This consistent attenuation of infection indicates that *hyd-2* is a potential virulence factor in *G. boninense*. The diminished colonisation ability and milder disease symptoms observed in ∆*hyd-2* mutants suggest a direct impairment in host surface attachment and invasion. Hydrophobins are known to mediate critical fungal functions such as aerial hyphae formation, surface hydrophobicity modulation, and host penetration. Frameshift mutations in *hyd-2*, particularly within conserved domains like the hydrophobic patch and cysteine-rich motifs, likely disrupt its structural integrity and biological activity, thereby weakening these essential pathogenic functions. Domain organisation and hydropathy analyses further support the classification of Hyd-2 as a class I hydrophobin, whose amphipathic profile is consistent with roles in aerial hypha development, surface adhesion, and spore hydrophobicity [57,111].

Previous studies across diverse fungal species similarly demonstrate the central role of hydrophobins in virulence. In *Magnaporthe oryzae*, deletion of *MPG1* disrupts appressorium formation and reduces infection efficiency [112], while *MHP1* deletion in *M. grisea* hinders conidiation and infectious growth, with phenotypes restored upon gene complementation [113]. In *Fusarium graminearum*, mutants lacking *FgHyd2* and *FgHyd3* showed reduced surface adhesion and virulence on wheat [56], and similar virulence defects have been observed in hydrophobin mutants of *Metarhizium brunneum* [114], *Cordyceps militaris* [57], and *Cryphonectria parasitica* [115].

Importantly, the impaired virulence of *G. boninense* ∆*hyd-2* mutants may not solely reflect a loss of infection machinery but could also stem from the inability to suppress or evade host defence responses. It is plausible that the reduced infection rate provides the host with additional time to activate defence mechanisms, thereby restricting or delaying disease progression. This hypothesis is supported by findings in *M. oryzae*, where hydrophobin-deficient mutants exhibit delayed appressorium formation, giving host plants a critical window for immune activation [116,117]. Similarly, *C. militaris* hydrophobin mutants show delayed virulence and infection structures, and in *Penicillium expansum*, deletion of the *creA* gene severely limits pathogenicity by failing to suppress host antioxidant responses [118]. These cases reinforce the idea that weakened pathogens—through loss of key virulence genes—can unintentionally allow the host to mount more effective resistance.

Furthermore, the strong upregulation of *hyd-2* during WT infection, as shown in earlier expression analysis (Figure 4), further supports its active role throughout the infection process. Its elevated expression during disease progression, contrasted with the reduced infective ability of the mutants, suggests that *hyd-2* is functionally required not only for early colonisation but also for maintaining host invasion at later stages. The failure of the mutants to sustain colonisation aligns with this model, reinforcing the importance of *hyd-2* in the full pathogenic lifecycle of *G. boninense*.

Thus, this study confirms that *hyd-2* contributes to virulence in *G. boninense*. Its disruption leads to both developmental defects and severe attenuation of pathogenicity, likely due to compromised surface adhesion and host penetration. These findings are consistent with the established roles of hydrophobins in other fungal pathogens and highlight *hyd-2* as a promising drug target for antifungal strategies aimed at controlling basal stem rot disease in oil palm.

### 4.4. Antifungal Development: Prospects, Strategic Implementation, and Future Perspectives

From an antifungal perspective, hydrophobin represents multiple potential intervention points, as it encodes a surface-localised, secreted proteins that is critical for fungal adhesion, modulation of surface hydrophobicity, and host tissue penetration—functions that render it potentially accessible to extracellular inhibitors [117]. Current strategies targeting such extracellular proteins include reducing agents or folding inhibitors that disrupt disulfide-bond stability [47,119]; peptide mimetics or antibodies that may interfere with the surface-assembly interface [120]; RNA-based silencing approaches such as spray-induced gene silencing or host-induced gene silencing (HIGS) that target transcript accumulation [121,122,123]; and small-molecule chemical compounds that may interfere with protein function or expression [124]. Collectively, these multiple strategies could potentially be further developed and formulated to specifically target *hyd-2* in future antifungal applications in both crop protection and fungal disease management. Moreover, due to its functional specificity and fungal exclusivity, *hyd-2* represents a low off-target risk for host plants and beneficial microbes [125]. Its expression pattern may also serve as a biomarker for aggressive isolates, offering diagnostic and predictive utility in field surveillance [56].

This study highlights the power of combining precise CRISPR/Cas9-mediated gene disruption with infection assays to validate virulence determinants and accelerate rational antifungal target discovery and deployment in *G. boninense*. From an applied perspective, this platform paves the way for systematic functional analysis of additional virulence factors, particularly those with structural or functional similarities to *hyd-2*. Such work could inform the rational development of antifungal strategies, either through the identification of molecular targets for chemical control or by guiding breeding programs aimed at enhancing host resistance.

### 4.5. Limitations of This Study and Future Considerations

Although in vitro infection assays offer rapid mechanistic insights, they do not fully capture the complexity of whole-plant defence responses or the soil–root–microbe interactions that shape disease outcomes. Current evidence indicates that *G. boninense* encodes at least 18 putative hydrophobin genes, inclusive of non-effector category based on the *G. boninense* G3 genomic databases, and 9 identified from classified as effectors as found in the transcriptomic database, suggesting that functional redundancy may allow other hydrophobins to compensate for the loss of *hyd-2* observed in this study. Such compensatory mechanisms may remain undetected in simplified in vitro systems but could be revealed under more complex host–pathogen interactions. For this reason, extended pathogenicity evaluations in nursery trials with diverse oil palm planting materials are essential to determine whether the absence of a single hydrophobin significantly affects virulence, or whether only simultaneous disruption of multiple hydrophobins would increase pathogen susceptibility to host detection and defence. These nursery-based assessments will be crucial for validating the role of *hyd-2* in infection biology and for evaluating its potential as a biomarker or antifungal target under field-relevant conditions.

Other limitations of this study should also be considered. To maximise the likelihood of successful CRISPR/Cas9-mediated genome editing, all sgRNAs were co-transformed in a single transformation. However, this approach precluded the evaluation of the individual editing efficiency of each sgRNA. Therefore, future studies should evaluate each sgRNA in a singular fashion to identify the most effective guide and establish optimal sgRNA design parameters for improving genome-editing efficiency in *G. boninense*.

Additionally, only two independent mutant lines were evaluated in the virulence assays; thus, a larger number of independent mutants is required to improve statistical robustness. In addition, complementation experiments are necessary to confirm that the observed ∆*hyd-2* phenotype is directly attributable to the targeted mutation rather than off-target effects. Furthermore, CRISPR/Cas9 application in dikaryotic fungi is constrained by nuclear heterogeneity, where two genetically distinct nuclei may undergo asynchronous editing, resulting in mixed genotypes containing both wild-type and edited alleles within a single transformant. This complicates the isolation of pure edited mutants and hampers clear genotype–phenotype interpretation. Future improvements should focus on optimizing editing strategies to achieve higher uniformity and efficiency across both nuclei, thereby enabling more reliable mutant selection and functional validation.

## 5. Conclusions

In this study, we established a CRISPR/Cas9 gene-editing framework for *G. boninense*, providing the first demonstration of stable mutant generation in this important oil palm pathogen. The successful disruption of *pyrG* validates the system as a functional molecular tool, while targeted mutagenesis of *hyd-2* has shown that it contributes to virulence. These findings offer new opportunities not just to dissect the contribution of hydrophobins to pathogenicity, but also has allowed for the establishment of a system for functional studies in *G. boninense*. Ultimately, the establishment of CRISPR/Cas9 editing in *G. boninense* represents a significant step forward in dissecting its pathogenic mechanisms and supports long-term efforts to manage basal stem rot through both molecular and agronomic interventions. Future enhancements should focus on improving editing efficiency and minimising incomplete editing, for example through optimised delivery systems, promoter selection, and repair template design, which would further strengthen the robustness of this platform. In aggregate, this framework paves the way for more systematic functional studies of virulence factors in *G. boninense*, ultimately advancing our understanding of pathogenicity mechanisms and enabling the development of targeted disease management strategies in oil palm.

## Figures and Tables

**Table 1:** Thermal cycling conditions for two-phase PCR amplification used in sgRNA template generation.

Steps	Temperature (°C)	Time	Cycle
Initial denaturation	95	1 min	
Denaturation	95	15 s	10 cycles
Annealing	50	15 s	
Extension	72	5 s	
Denaturation	95	15 s	20 cycles
Annealing	55	15 s	
Extension	72	5 s	
Final extension	72	1 min	
Pause	15	-	

**Table 2:** Sequences and characteristics of sgRNA forward primers designed to target the *pyrG* gene.

No	Name	Sequence (5′ → 3′)	Orientation	CRISPRater	% GC
1	Fwd.sgpyrG1	**CCTCTAATACGACTCACTATA** GG CGCGATCTTGTGTACCCCTG **GTTTTAGAGCTAGAA**	antisense	0.74	58.3
2	Fwd.sgpyrG2	**CCTCTAATACGACTCACTATA** G GTTTGTGCGCTTGCGCTCGA **GTTTTAGAGCTAGAA**	antisense	0.71	75.0
3	Fwd.sgpyrG3	**CCTCTAATACGACTCACTATA** G GTTTTCGGCTGAGTGCTTCG **GTTTTAGAGCTAGAA**	antisense	0.83	58.3
4	Rvs.sguniversal	AAAAGCACCGACTCGGTGCCACTTTTTCAAGTTG			

Note. CCTC: An additional 5′-CCTC sequence was incorporated into each primer as recommended by the manufacturer (Takara Bio Inc., Shiga, Japan) to improve PCR amplification efficiency. Black Bold: T7 promoter sequence (17 bp). Underline sequence: Underlined sequence: the selected potential sgRNA sequence from the analysis. Grey shaded: extra G (one or two G) added at the 5′- of the target sequence as T7 promoter requires at least 2 Gs for efficient transcription. Blue Bold: Scaffold Template annealing sequence (15 bp); % GC: % GC Content at 20 bp seed sequence.

**Table 3:** Sequences and characteristics of sgRNA forward primers designed to target the *hyd-2* gene.

No	Name	Sequence (5′ → 3′)	Orientation	CRISPRater	% GC
1	Fwd.sghyd2-1	**CCTCTAATACGACTCACTATA** GG CCTCGCTCTGCCCCTCCTTG **GTTTTAGAGCTAGAA**	Sense	0.87	70
2	Fwd.sghyd2-2	**CCTCTAATACGACTCACTATA** GG GTGCTGCAACAGCACCGTCG **GTTTTAGAGCTAGAA**	Sense	0.86	65
3	Fwd.sghyd2-3	**CCTCTAATACGACTCACTATA** GG ATTGTCCTCGGGGACATCAC **GTTTTAGAGCTAGAA**	Sense	0.83	58.33
4	Rvs.sguniversal	AAAAGCACCGACTCGGTGCCACTTTTTCAAGTTG			

Note. CCTC: An additional 5′-CCTC sequence was incorporated into each primer as recommended by the manufacturer (Takara Bio Inc., Shiga, Japan) to improve PCR amplification efficiency. Black Bold: T7 promoter sequence (17 bp). Underline sequence: Underlined sequence: the selected potential sgRNA sequence from the analysis. Grey shaded: extra G (one or two G) added at the 5′- of the target sequence as T7 promoter requires at least 2 Gs for efficient transcription. Blue Bold: Scaffold Template annealing sequence (15 bp); % GC: % GC Content at 20 bp seed sequence.

## Data Availability

Some of the data used in this project, particularly the PacBio Iso-seq transcriptomic databases employed in identifying potential gene targets, are confidential and therefore not publicly available.

## Supplementary Materials

The following supporting information can be downloaded at: https://www.mdpi.com/article/10.3390/jof12080594/s1. Figure S1. Minimum Inhibitory Concentration (MIC) analysis for *G. boninense* PER71 on 5-fluoroacetic acid (5-FOA) and hygromycin. Figure S2. DNA sequences of the *pyrG* gene in *G. boninense* PER71 and alignment of the genomic sequence, transcript, and sgRNA target sites. (A) Full length genomic sequence of the *pyrG* gene; (B) Corresponding transcript (cDNA) sequence; (C) Multiple sequence alignment between the *pyrG* gene and transcript and location of the designed sgRNA (sgRNA1, sgRNA2 and sgRNA3). Figure S3. Verification of plasmid uptake and *cas9* expression in protoplast transformants via PCR and RT-PCR analysis. (A) Amplicons of the expected partial *cas9* (~680 bp) sequence were detected using total DNA extraction from transformants. Legend: M: GeneRuler 1 kb DNA Ladder (ThermoFisher Scientific, USA); C: positive control (pGreen0179Glcas9); 1: transformants exhibiting hygromycin resistance on transformation plates containing 110 µg/mL of hygromycin B. (B) RT-PCR analysis confirming *cas9* gene expression using cDNA templates synthesised from the first generation of transformants. Amplification of the partial *cas9* gene from cDNA templates confirms active gene expression in the transformants. A thin band was observed, indicating low expression levels. This low abundance is likely driven by the low copy number of the episomal plasmid and a dilution effect across generations. Legend: 1: stock cDNA; 2: 1:5 diluted cDNA, 3: 1:10 diluted cDNA; M: GeneRuler 1 kb DNA Ladder (ThermoFisher Scientific, USA); C: positive control (pGreen0179Glcas9). Figure S4. Sequencing analysis of the *pyrG* gene in wild-type (WT) and putative mutant strains. (A) *pyrG* sequencing result for the wild type, ΔpyrG-1 and ΔpyrG-2; (B) Multiple sequence alignment (MSA) between wild-type *pyrG* (Gb|pyrGC) and mutant, ΔpyrG-1; (C) BLAST analysis on the inserted sequence revealed up to 98.94% sequence similarity with the sequence located at LG3, LG4 and 91–94% sequence similarity with LG2 and LG10; (D) Multiple sequence alignment (MSA) between wild-type *pyrG* (Gb|pyrGC) and mutant, ΔpyrG-2; (E) Further BLASTN analysis of the 277 bp extra sequence inserted in *pyrG* gene in ΔpyrG-2 revealed a strong match to scaffold LG8 of the *G. boninense* G3 genome, with 98% coverage and 99.64% sequence identity, indicating that the insertion likely originated from an internal genomic fragment rather than exogenous contamination. Figure S5. DNA sequences of the *hyd-2* gene in *G. boninense* PER71 and alignment of the genomic sequence, transcript, and sgRNA target sites. (A) Full length genomic sequence of the *hyd-*2 gene; (B) Corresponding transcript (cDNA) sequence; (C) Multiple sequence alignment between the *hyd-2* gene and transcript and location of the designed sgRNA (sgRNA1, sgRNA2 and sgRNA3). Figure S6. Sequencing analysis of the *hyd-2* cDNA in wild-type (WT) and putative mutant strains. (A) Validation of the putative *hyd–2* mutants were performed through sequencing analysis. Multiple sequence alignment of *hyd–2* amplicons from ∆hyd2–44, ∆hyd2–45, ∆hyd2–15, and ∆hyd2–16 against the wild-type sequence revealed base substitution mutations distributed across the gene, highlighted in red; (B) Multiple sequence alignment of *hyd-2* amplicons from ∆hyd2-19, ∆hyd2–49, ∆hyd2–11, and ∆hyd2–8 with the wild-type sequence revealed diverse mutation types—including base substitutions, deletions, and insertions—highlighted in red. Figure S7. Detailed analysis of the hydrophobin-2 protein. (A) Kyte–Doolittle hydropathy plot of the hydrophobin-2 protein showing alternating hydrophobic and hydrophilic regions. A major hydrophobic peak at positions 35–40 suggests a potential self-assembly domain typical of hydrophobins; (B) InterPro annotation of the hydrophobin protein. Domain architecture and hydropathy profile of the hydrophobin protein. Table S1. The primer sequences used in this project. Table S2. Two-Way Repeated Measures ANOVA of Disease Severity Index (DSI) Scores. Summary of the effects of genotype, time (weeks 3–7), and their interaction on DSI scores. Table S3. RT-qPCR expression profiles of individual hydrophobin genes. Table S4. The hydrophobin-2 protein sequences used in the construction of phylogenetic tree analysis.

## Abbreviations

The following abbreviations are used in this manuscript:

CRISPR	Clustered Regularly Interspaced Short Palindromic Repeats
BSR	Basal stem rot
USR	Upper stem rot
UMP	Uridine 5′-monophosphate
*pyrG*	*orotidine-5′-monophosphate decarboxylase*
TALENs	Transcription Activator-Like Effector Nuclease
PDA	Potato Dextrose Agar
WT	Wild-type
5-FOA	5-fluoroorotic acid
MM	Minimal media
Hyd/*hyd*	Hydrophobin
dpi	Day-post inoculation
hpi	Hour-post inoculation
wpi	Week-post inoculation
sgRNA	Single guide RNA
tracrRNA	Trans-activating CRISPR RNA
DSB	Double-strand breaks
HIGS	Host-induced gene silencing
MMEJ	Microhomology-mediated end joining
PAM	Protospacer adjacent motif
HDR	Homology-directed repair

## References

[1] Midot F., Lau S.Y.L., Wong W.C., Tung H.J., Yap M.L., Lo M.L., Jee M.S., Dom S.P., Melling L. (2019). Genetic diversity and demographic history of *Ganoderma boninense* in oil palm plantations of Sarawak, Malaysia inferred from ITS regions. Microorganisms.

[2] Hussain H., Aspar H.M., Mazlan R., Nadras S., Ab Rahman M.A.K., Hassan N.A.M., Minal J. (2025). Palm Oil Business: Global Market Trend and Local Outlook. The Palm Oil Export Market.

[3] Jazuli N.A., Kamu A., Chong K.P., Gabda D., Hassan A., Abu Seman I., Ho C.M. (2022). A review of factors affecting Ganoderma basal stem rot disease progress in oil palm. Plants.

[4] Hassan M.A., Farid M.A.A., Zakaria M.R., Ariffin H., Andou Y., Shirai Y. (2024). Palm oil expansion in Malaysia and its countermeasures through policy window and biorefinery approach. Environ. Sci. Policy.

[5] Parveez G.K.A., Hishamuddin E., Loh S.K., Ong-Abdullah M., Salleh K.M., Bidin M., Sundram S., Hasan Z.A.A., Idris Z. (2020). Oil palm economic performance in Malaysia and R&D progress in 2019. J. Oil Palm. Res..

[6] Naidu L., Moorthy R. (2021). A review of key sustainability issues in Malaysian palm oil industry. Sustainability.

[7] Gesteiro E., Guijarro L., Sánchez-Muniz F.J., Vidal-Carou M.d.C., Troncoso A., Venanci L., Jimeno V., Quilez J., Anadón A., González-Gross M. (2019). Palm oil on the edge. Nutrients.

[8] Hariyanti F., Syahza A., Zulkarnain N. (2022). Sustainability of the Palm Oil Industry: An Empirical Study of the Development of Sustainable Oil Palm in Bengkalis Regency, Indonesia. Int. J. Sustain. Dev. Plan.

[9] Abubakar A., Ishak M.Y., Bakar A.A., Uddin M.K. (2022). *Ganoderma boninense* basal stem rot induced by climate change and its effect on oil palm. Environ. Sustain..

[10] Zakaria L. (2023). Basal stem rot of oil palm: The pathogen, disease incidence, and control methods. Plant Dis..

[11] Darlis D., Jalloh M.B., Chin C.F.S., Basri N.K.M., Besar N.A., Ahmad K., Rakib M.R.M. (2023). Exploring the potential of Bornean polypore fungi as biological control agents against pathogenic *Ganoderma boninense* causing basal stem rot in oil palm. Sci. Rep..

[12] Khoo Y.W., Chong K.P. (2023). *Ganoderma boninense*: General characteristics of pathogenicity and methods of control. Front. Plant Sci..

[13] Virdiana I., Hasan Y., Aditya R., Flood J. (2010). Testing the effects of oil palm replanting practices (windrowing, fallowing and poisoning) on incidence of Ganoderma. Proc. IOPC.

[14] Chong K.-P., Alexander A. (2016). Early detection and management of Ganoderma basal stem rot disease: A special report from sabah. Trans. Sci. Technol..

[15] Susanto A., Sudharto P., Purba R. (2005). Enhancing biological control of basal stem rot disease (*Ganoderma boninense*) in oil palm plantations. Mycopathologia.

[16] Ab Wahab A.F.F., Zairun M.A., Daud K.H.M., Bakar F.D.A., Bharudin I., Murad A.M.A. (2022). Evaluation and improvement of protocols for *Ganoderma boninense* protoplast isolation and regeneration. Malays. Appl. Biol..

[17] Idris A., Kushairi A., Ismail S., Ariffin D. (2004). Selection for partial resistance in oil palm progenies to Ganoderma basal stem rot. J. Oil Palm. Res..

[18] Rees R., Flood J., Hasan Y., Potter U., Cooper R.M. (2009). Basal stem rot of oil palm (*Elaeis guineensis*); mode of root infection and lower stem invasion by *Ganoderma boninense*. Plant Pathol..

[19] Rees R., Flood J., Hasan Y., Wills M.A., Cooper R.M. (2012). *Ganoderma boninense* basidiospores in oil palm plantations: Evaluation of their possible role in stem rots of *Elaeis guineensis*. Plant Pathol..

[20] Utomo B., Riatmaja D.S., Aninam J., Wahyudi F. (2024). Environmental sustainability and economic growth: Policy implications for developing countries. Nomico J..

[21] Dhillon B., Hamelin R.C., Rollins J.A. (2021). Transcriptional profile of oil palm pathogen, *Ganoderma boninense*, reveals activation of lignin degradation machinery and possible evasion of host immune response. BMC Genom..

[22] Khairi M.H.F., Nor Muhammad N.A., Bunawan H., Abdul Murad A.M., Ramzi A.B. (2022). Unveiling the core effector proteins of oil palm pathogen *Ganoderma boninense* via pan-secretome analysis. J. Fungi.

[23] Lim F.-H., Abd Rasid O., Idris A.S., As’wad A.W.M., Vadamalai G., Parveez G.K.A., Wong M.-Y. (2024). Gene silencing of *Ganoderma boninense* Lanosterol 14α-demethylase (GBERG11) affects ergosterol biosynthesis and pathogenicity towards oil palm. Sci. Hortic..

[24] Armas-Tizapantzi A., Montiel-González A.M. (2016). RNAi silencing: A tool for functional genomics research on fungi. Fungal Biol. Rev..

[25] Senthil-Kumar M., Mysore K.S. (2011). New dimensions for VIGS in plant functional genomics. Trends Plant Sci..

[26] Svoboda P. (2020). Key mechanistic principles and considerations concerning RNA interference. Front. Plant Sci..

[27] Ferreira P., Choupina A.B. (2022). CRISPR/Cas9 a simple, inexpensive and effective technique for gene editing. Mol. Biol. Rep..

[28] Janik E., Niemcewicz M., Ceremuga M., Krzowski L., Saluk-Bijak J., Bijak M. (2020). Various aspects of a gene editing system—Crispr–cas9. Int. J. Mol. Sci..

[29] Khalil A.M. (2020). The genome editing revolution. J. Genet. Eng. Biotechnol..

[30] Aljabali A.A., El-Tanani M., Tambuwala M.M. (2024). Principles of CRISPR-Cas9 technology: Advancements in genome editing and emerging trends in drug delivery. J. Drug Deliv. Sci. Technol..

[31] Khan M.S., Qureshi N., Khan R., Son Y.-O., Maqbool T. (2025). CRISPR/Cas9-Based therapeutics as a promising strategy for management of Alzheimer’s disease: Progress and prospects. Front. Cell. Neurosci..

[32] Afridi M.S.K., Farhan U., Mushtaq A., Ahmad A., Rana H., Naseer S., Mushtaq S., Ahmad M. (2025). CRISPR Applications in Enhancing Medicinal Plant Yields. Sch. Acad. J. Biosci..

[33] Schuster M., Schweizer G., Reissmann S., Kahmann R. (2016). Genome editing in *Ustilago maydis* using the CRISPR–Cas system. Fungal Genet. Biol..

[34] Ökmen B., Schwammbach D., Bakkeren G., Neumann U., Doehlemann G. (2021). The *Ustilago hordei*–barley interaction is a versatile system for characterization of fungal effectors. J. Fungi.

[35] Lu S., Wang Y., Shen X., Guo F., Zhou C., Li R., Chen B. (2021). SsPEP1, an effector with essential cellular functions in sugarcane smut fungus. J. Fungi.

[36] Liu K., Sun B., You H., Tu J.L., Yu X., Zhao P., Xu J.W. (2020). Dual sgRNA-directed gene deletion in basidiomycete *Ganoderma lucidum* using the CRISPR/Cas9 system. Microb. Biotechnol..

[37] Tu J.-L., Bai X.-Y., Xu Y.-L., Li N., Xu J.-W. (2021). Targeted gene insertion and replacement in the basidiomycete *Ganoderma lucidum* by inactivation of nonhomologous end joining using CRISPR/Cas9. Appl. Environ. Microbiol..

[38] Wang P.-A., Zhang J.-M., Zhong J.-J. (2022). CRISPR-Cas9 assisted in-situ complementation of functional genes in the basidiomycete *Ganoderma lucidum*. Process Biochem..

[39] Choi Y.-J., Eom H., Nandre R., Kim M., Oh Y.-L., Kim S., Ro H.-S. (2025). Simultaneous gene editing of both nuclei in a dikaryotic strain of *Ganoderma lucidum* using Cas9-gRNA ribonucleoprotein. J. Microbiol..

[40] Boeke J.D., Trueheart J., Natsoulis G., Fink G.R. (1987). [10] 5-Fluoroorotic acid as a selective agent in yeast molecular genetics. Methods in Enzymology.

[41] Zhang H., Ji S., Guo R., Zhou C., Wang Y., Fan H., Liu Z. (2019). Hydrophobin HFBII-4 from *Trichoderma asperellum* induces antifungal resistance in poplar. Braz. J. Microbiol..

[42] Cai F., Gao R., Zhao Z., Ding M., Jiang S., Yagtu C., Zhu H., Zhang J., Ebner T., Mayrhofer-Reinhartshuber M. (2020). Evolutionary compromises in fungal fitness: Hydrophobins can hinder the adverse dispersal of conidiospores and challenge their survival. ISME J..

[43] Wösten H.A., de Vocht M.L. (2000). Hydrophobins, the fungal coat unravelled. Biochim. Biophys. Acta BBA-Rev. Biomembr..

[44] Blango M.G., Kniemeyer O., Brakhage A.A. (2019). Conidial surface proteins at the interface of fungal infections. PLoS Pathog..

[45] Kamiya A., Ueshima H., Nishida S., Honda Y., Kamitsuji H., Sato T., Miyamoto H., Sumita T., Izumitsu K., Irie T. (2023). Development of a gene-targeting system using CRISPR/Cas9 and utilization of pyrG as a novel selectable marker in *Lentinula edodes*. FEMS Microbiol. Lett..

[46] Fujihashi M., Mnpotra J.S., Mishra R.K., Pai E.F., Kotra L.P. (2015). Orotidine Monophosphate Decarboxylase–A Fascinating Workhorse Enzyme with Therapeutic Potential. J. Genet. Genom..

[47] Rojas-Osnaya J., Quintana-Quirino M., Espinosa-Valencia A., Bravo A.L., Nájera H. (2024). Hydrophobins: Multitask proteins. Front. Phys..

[48] Carter-House D., Stajich J., Unruh S., Kurbessoian T. (2020). Fungal CTAB DNA Extraction.

[49] Riffiani R., Wada T., Shimomura N., Yamaguchi T., Aimi T. (2021). An optimized method for high-quality DNA extraction medicinal fungi Mycoleptodonoides aitchisonii for whole genome sequencing. Proc. IOP Conf. Ser. Earth Environ. Sci..

[50] Brinkman E.K., Kousholt A.N., Harmsen T., Leemans C., Chen T., Jonkers J., Van Steensel B. (2018). Easy quantification of template-directed CRISPR/Cas9 editing. Nucleic Acids Res..

[51] Conant D., Hsiau T., Rossi N., Oki J., Maures T., Waite K., Yang J., Joshi S., Kelso R., Holden K. (2022). Inference of CRISPR edits from Sanger trace data. Cris. J..

[52] Teufel F., Almagro Armenteros J.J., Johansen A.R., Gíslason M.H., Pihl S.I., Tsirigos K.D., Winther O., Brunak S., von Heijne G., Nielsen H. (2022). SignalP 6.0 predicts all five types of signal peptides using protein language models. Nat. Biotechnol..

[53] Savojardo C., Martelli P.L., Fariselli P., Casadio R. (2018). DeepSig: Deep learning improves signal peptide detection in proteins. Bioinformatics.

[54] Käll L., Krogh A., Sonnhammer E.L. (2007). Advantages of combined transmembrane topology and signal peptide prediction—The Phobius web server. Nucleic Acids Res..

[55] Ronquist F., Teslenko M., Van Der Mark P., Ayres D.L., Darling A., Höhna S., Larget B., Liu L., Suchard M.A., Huelsenbeck J.P. (2012). MrBayes 3.2: Efficient Bayesian phylogenetic inference and model choice across a large model space. Syst. Biol..

[56] Quarantin A., Hadeler B., Kröger C., Schäfer W., Favaron F., Sella L., Martínez-Rocha A.L. (2019). Different hydrophobins of *Fusarium graminearum* are involved in hyphal growth, attachment, water-air interface penetration and plant infection. Front. Microbiol..

[57] Bayry J., Aimanianda V., Guijarro J.I., Sunde M., Latge J.-P. (2012). Hydrophobins—Unique fungal proteins. PLoS Pathog..

[58] Qiao J., Liu H., Xue P., Hong M., Guo X., Xing Z., Zhao M., Zhu J. (2023). Function of a hydrophobin in growth and development, nitrogen regulation, and abiotic stress resistance of *Ganoderma lucidum*. FEMS Microbiol. Lett..

[59] Carrion S.d.J., Leal S.M., Ghannoum M.A., Aimanianda V., Latgé J.-P., Pearlman E. (2013). The rodA hydrophobin on *Aspergillus fumigatus* spores masks dectin-1–and dectin-2–dependent responses and enhances fungal survival in vivo. J. Immunol..

[60] Valsecchi I., Dupres V., Stephen-Victor E., Guijarro J., Gibbons J., Beau R., Bayry J., Coppee J., Lafont F., Latgé J. (2017). Role of hydrophobins in *Aspergillus fumigatus*. J. Fungi.

[61] Miguel-Rojas C., Cavinder B., Townsend J.P., Trail F. (2023). Comparative transcriptomics of *Fusarium graminearum* and *Magnaporthe oryzae* spore germination leading up to infection. mBio.

[62] Tanaka T., Terauchi Y., Yoshimi A., Abe K. (2022). Aspergillus hydrophobins: Physicochemical properties, biochemical properties, and functions in solid polymer degradation. Microorganisms.

[63] Kouranova E., Forbes K., Zhao G., Warren J., Bartels A., Wu Y., Cui X. (2016). CRISPRs for optimal targeting: Delivery of CRISPR components as DNA, RNA, and protein into cultured cells and single-cell embryos. Hum. Gene Ther..

[64] Saikia B., Remya S., Debbarma J., Maharana J., Sastry G.N., Chikkaputtaiah C. (2024). CRISPR/Cas9-based genome editing and functional analysis of SlHyPRP1 and SlDEA1 genes of *Solanum lycopersicum* L. in imparting genetic tolerance to multiple stress factors. Front. Plant Sci..

[65] Karvelis T., Gasiunas G., Miksys A., Barrangou R., Horvath P., Siksnys V. (2013). crRNA and tracrRNA guide Cas9-mediated DNA interference in *Streptococcus thermophilus*. RNA Biol..

[66] Liao C., Beisel C.L. (2021). The tracrRNA in CRISPR biology and technologies. Annu. Rev. Genet..

[67] Riesenberg S., Helmbrecht N., Kanis P., Maricic T., Pääbo S. (2022). Improved gRNA secondary structures allow editing of target sites resistant to CRISPR-Cas9 cleavage. Nat. Commun..

[68] Li J., Kong D., Ke Y., Zeng W., Miki D. (2024). Application of multiple sgRNAs boosts efficiency of CRISPR/Cas9-mediated gene targeting in *Arabidopsis*. BMC Biol..

[69] Yang H., Wu J.-J., Tang T., Liu K.-D., Dai C. (2017). CRISPR/Cas9-mediated genome editing efficiently creates specific mutations at multiple loci using one sgRNA in *Brassica napus*. Sci. Rep..

[70] Kosicki M., Allen F., Steward F., Tomberg K., Pan Y., Bradley A. (2022). Cas9-induced large deletions and small indels are controlled in a convergent fashion. Nat. Commun..

[71] Liu G., Lin Q., Jin S., Gao C. (2022). The CRISPR-Cas toolbox and gene editing technologies. Mol. Cell.

[72] Yuan B., Bi C., Tian Y., Wang J., Jin Y., Alsayegh K., Tehseen M., Yi G., Zhou X., Shao Y. (2024). Modulation of the microhomology-mediated end joining pathway suppresses large deletions and enhances homology-directed repair following CRISPR-Cas9-induced DNA breaks. BMC Biol..

[73] Danchik C., Casadevall A. (2021). Role of cell surface hydrophobicity in the pathogenesis of medically-significant fungi. Front. Cell. Infect. Microbiol..

[74] Almagro Armenteros J.J., Tsirigos K.D., Sønderby C.K., Petersen T.N., Winther O., Brunak S., von Heijne G., Nielsen H. (2019). SignalP 5.0 improves signal peptide predictions using deep neural networks. Nat. Biotechnol..

[75] Yim C., Chung Y., Kim J., Nilsson I., Kim J.-S., Kim H. (2021). Spc1 regulates the signal peptidase-mediated processing of membrane proteins. J. Cell Sci..

[76] Chung Y., Yim C., Pereira G.P., Son S., Kjølbye L.R., Mazurkiewicz L.E., Weeks A.M., Förster F., von Heijne G., Souza P.C. (2024). Spc2 modulates substrate-and cleavage site-selection in the yeast signal peptidase complex. J. Cell Biol..

[77] Owji H., Nezafat N., Negahdaripour M., Hajiebrahimi A., Ghasemi Y. (2018). A comprehensive review of signal peptides: Structure, roles, and applications. Eur. J. Cell Biol..

[78] Mgbeahuruike A.C., Kovalchuk A., Chen H., Ubhayasekera W., Asiegbu F.O. (2013). Evolutionary analysis of hydrophobin gene family in two wood-degrading basidiomycetes, *Phlebia brevispora* and Heterobasidion annosum sl. BMC Evol. Biol..

[79] Morris V.K., Ren Q., Macindoe I., Kwan A.H., Byrne N., Sunde M. (2011). Recruitment of class I hydrophobins to the air: Water interface initiates a multi-step process of functional amyloid formation. J. Biol. Chem..

[80] Siddiquee R., Lo V., Johnston C.L., Buffier A.W., Ball S.R., Ciofani J.L., Zeng Y.C., Mahjoub M., Chrzanowski W., Rezvani-Baboli S. (2023). Surface-induced hydrophobin assemblies with versatile properties and distinct underlying structures. Biomacromolecules.

[81] Vergunst K.L., Langelaan D.N. (2022). The N-terminal tail of the hydrophobin SC16 is not required for rodlet formation. Sci. Rep..

[82] Dort E.N., Tanguay P., Hamelin R.C. (2020). CRISPR/Cas9 gene editing: An unexplored frontier for forest pathology. Front. Plant Sci..

[83] Liu R., Chen L., Jiang Y., Zhou Z., Zou G. (2015). Efficient genome editing in filamentous fungus *Trichoderma reesei* using the CRISPR/Cas9 system. Cell Discov..

[84] Paul N.C., Park S.-W., Liu H., Choi S., Ma J., MacCready J.S., Chilvers M.I., Sang H. (2021). Plant and fungal genome editing to enhance plant disease resistance using the CRISPR/Cas9 system. Front. Plant Sci..

[85] Liao B., Chen X., Zhou X., Zhou Y., Shi Y., Ye X., Liao M., Zhou Z., Cheng L., Ren B. (2022). Applications of CRISPR/Cas gene-editing technology in yeast and fungi. Arch. Microbiol..

[86] Nanasato Y., Kawabe H., Ueno S., Konagaya K.-i., Endo M., Taniguchi T. (2024). Improvement of genome editing efficiency by Cas9 codon optimization in Japanese cedar (*Cryptomeria japonica* D. Don). Plant Biotechnol..

[87] Koshi D., Ueshima H., Kawauchi M., Nakazawa T., Sakamoto M., Hirata M., Izumitsu K., Sumita T., Irie T., Honda Y. (2022). Marker-free genome editing in the edible mushroom, *Pleurotus ostreatus*, using transient expression of genes required for CRISPR/Cas9 and for selection. J. Wood Sci..

[88] Woods J.P., Heinecke E.L., Goldman W.E. (1998). Electrotransformation and expression of bacterial genes encoding hygromycin phosphotransferase and β-galactosidase in the pathogenic fungus *Histoplasma capsulatum*. Infect. Immun..

[89] Nødvig C.S., Nielsen J.B., Kogle M.E., Mortensen U.H. (2015). A CRISPR-Cas9 system for genetic engineering of filamentous fungi. PLoS ONE.

[90] Arazoe T., Miyoshi K., Yamato T., Ogawa T., Ohsato S., Arie T., Kuwata S. (2015). Tailor-made CRISPR/Cas system for highly efficient targeted gene replacement in the rice blast fungus. Biotechnol. Bioeng..

[91] Grüttner S., Kempken F. (2024). A user-friendly CRISPR/Cas9 system for mutagenesis of *Neurospora crassa*. Sci. Rep..

[92] Schmidt H., Zhang M., Chakarov D., Bansal V., Mourelatos H., Sánchez-Rivera F.J., Lowe S.W., Ventura A., Leslie C.S., Pritykin Y. (2025). Genome-wide CRISPR guide RNA design and specificity analysis with GuideScan2. Genome Biol..

[93] Dong C., Gou Y., Lian J. (2022). SgRNA engineering for improved genome editing and expanded functional assays. Curr. Opin. Biotechnol..

[94] Mengstie M.A., Azezew M.T., Dejenie T.A., Teshome A.A., Admasu F.T., Teklemariam A.B., Mulu A.T., Agidew M.M., Adugna D.G., Geremew H. (2024). Recent advancements in reducing the off-target effect of CRISPR-Cas9 genome editing. Biol. Targets Ther..

[95] Jung W.J., Park S.-J., Cha S., Kim K. (2024). Factors affecting the cleavage efficiency of the CRISPR-Cas9 system. Anim. Cells Syst..

[96] Li C., Chu W., Gill R.A., Sang S., Shi Y., Hu X., Yang Y., Zaman Q.U., Zhang B. (2023). Computational tools and resources for CRISPR/Cas genome editing. Genom. Proteom. Bioinform..

[97] Chen Y., Wang X. (2022). Evaluation of efficiency prediction algorithms and development of ensemble model for CRISPR/Cas9 gRNA selection. Bioinformatics.

[98] Zhang L., Zheng X., Cairns T.C., Zhang Z., Wang D., Zheng P., Sun J. (2020). Disruption or reduced expression of the orotidine-5′-decarboxylase gene pyrG increases citric acid production: A new discovery during recyclable genome editing in *Aspergillus niger*. Microb. Cell Fact..

[99] Rehman L., Su X., Guo H., Qi X., Cheng H. (2016). Protoplast transformation as a potential platform for exploring gene function in *Verticillium dahliae*. BMC Biotechnol..

[100] Schindele P., Wolter F., Puchta H. (2020). CRISPR guide RNA design guidelines for efficient genome editing. RNA Tagging: Methods and Protocols.

[101] Kosicki M., Tomberg K., Bradley A. (2018). Repair of double-strand breaks induced by CRISPR–Cas9 leads to large deletions and complex rearrangements. Nat. Biotechnol..

[102] Owens D.D., Caulder A., Frontera V., Harman J.R., Allan A.J., Bucakci A., Greder L., Codner G.F., Hublitz P., McHugh P.J. (2019). Microhomologies are prevalent at Cas9-induced larger deletions. Nucleic Acids Res..

[103] Boroviak K., Fu B., Yang F., Doe B., Bradley A. (2017). Revealing hidden complexities of genomic rearrangements generated with Cas9. Sci. Rep..

[104] Chakrabarti A.M., Henser-Brownhill T., Monserrat J., Poetsch A.R., Luscombe N.M., Scaffidi P. (2019). Target-specific precision of CRISPR-mediated genome editing. Mol. Cell.

[105] Hwang G.-H., Yu J., Yang S., Son W.J., Lim K., Kim H.S., Kim J.-S., Bae S. (2020). CRISPR-sub: Analysis of DNA substitution mutations caused by CRISPR-Cas9 in human cells. Comput. Struct. Biotechnol. J..

[106] Clarke L., Rebelo C., Goncalves J., Boavida M., Jordan P. (2001). PCR amplification introduces errors into mononucleotide and dinucleotide repeat sequences. Mol. Pathol..

[107] Fox E.J., Reid-Bayliss K.S., Emond M.J., Loeb L.A. (2014). Accuracy of next generation sequencing platforms. Next Gener. Seq. Appl..

[108] Glenn T.C. (2011). Field guide to next-generation DNA sequencers. Mol. Ecol. Resour..

[109] Shumega A.R., Pavlov Y.I., Chirinskaite A.V., Rubel A.A., Inge-Vechtomov S.G., Stepchenkova E.I. (2024). CRISPR/Cas9 as a mutagenic factor. Int. J. Mol. Sci..

[110] Xiao Z., Ying W., Xing Z., Zhihui L., Qiuyu Z., Caijiao H., Changlong L., Shi H., Deng L., Zhenwen C. (2024). Unexpected mutations occurred in CRISPR/Cas9 edited *Drosophila* analyzed by deeply whole genomic sequencing. Heliyon.

[111] Jensen B.G., Andersen M.R., Pedersen M.H., Frisvad J.C., Søndergaard I. (2010). Hydrophobins from Aspergillus species cannot be clearly divided into two classes. BMC Res. Notes.

[112] Talbot N.J., Ebbole D.J., Hamer J.E. (1993). Identification and characterization of MPG1, a gene involved in pathogenicity from the rice blast fungus *Magnaporthe grisea*. Plant Cell.

[113] Kim S., Ahn I.P., Rho H.S., Lee Y.H. (2005). MHP1, a *Magnaporthe grisea* hydrophobin gene, is required for fungal development and plant colonization. Mol. Microbiol..

[114] Sevim A., Donzelli B.G., Wu D., Demirbag Z., Gibson D.M., Turgeon B.G. (2012). Hydrophobin genes of the entomopathogenic fungus, *Metarhizium brunneum*, are differentially expressed and corresponding mutants are decreased in virulence. Curr. Genet..

[115] Kazmierczak P., Kim D.H., Turina M., Van Alfen N.K. (2005). A hydrophobin of the chestnut blight fungus, *Cryphonectria parasitica*, is required for stromal pustule eruption. Eukaryot. Cell.

[116] Beckerman J.L., Ebbole D. (1996). MPG1, a gene encoding a fungal hydrophobin of *Magnaporthe grisea*, is involved in surface recognition. Mol. Plant-Microbe Interact. MPMI.

[117] Kershaw M.J., Talbot N.J. (2009). Genome-wide functional analysis reveals that infection-associated fungal autophagy is necessary for rice blast disease. Proc. Natl. Acad. Sci. USA.

[118] Tannous J., Kumar D., Sela N., Sionov E., Prusky D., Keller N.P. (2018). Fungal attack and host defence pathways unveiled in near-avirulent interactions of *Penicillium expansum* creA mutants on apples. Mol. Plant Pathol..

[119] Nada H., Choi Y., Kim S., Jeong K.S., Meanwell N.A., Lee K. (2024). New insights into protein–protein interaction modulators in drug discovery and therapeutic advance. Signal Transduct. Target. Ther..

[120] Sarkar A., Roy-Barman S. (2021). Spray-induced silencing of pathogenicity gene MoDES1 via exogenous double-stranded RNA can confer partial resistance against fungal blast in rice. Front. Plant Sci..

[121] Koch A., Kumar N., Weber L., Keller H., Imani J., Kogel K.-H. (2013). Host-induced gene silencing of cytochrome P450 lanosterol C14α-demethylase–encoding genes confers strong resistance to Fusarium species. Proc. Natl. Acad. Sci. USA.

[122] Wang M., Dean R.A. (2022). Host induced gene silencing of *Magnaporthe oryzae* by targeting pathogenicity and development genes to control rice blast disease. Front. Plant Sci..

[123] Wang M., Jin H. (2017). Spray-induced gene silencing: A powerful innovative strategy for crop protection. Trends Microbiol..

[124] Bahn Y.-S. (2015). Exploiting fungal virulence-regulating transcription factors as novel antifungal drug targets. PLoS Pathog..

[125] Wösten H.A., Scholtmeijer K. (2015). Applications of hydrophobins: Current state and perspectives. Appl. Microbiol. Biotechnol..

