# Supplementary material for: Establishment of CRISPR/Cas9 Genome Editing in Ganoderma boninense and Functional Validation of Hydrophobin-2 as a Virulence Determinant
Source: J Fungi (Basel). 2026 Aug 11;12(8):594. doi: 10.3390/jof12080594 (PMC13514564; doi:10.3390/jof12080594)
Supplement: Supplementary file 1 [file jof-12-00594-s001.zip › jof-4408169-supplementary.pdf]

Supplementary Material

Establishment of CRISPR/Cas9 Genome Editing in *Ganoderma boninense* and Functional Validation of Hydrophobin-2 as a Virulence Determinant

Anis Farhan Fatimi Ab Wahab [1,*], Mohd Azinuddin Ahmad Mokhtar [2], Sharmilah Vetaryan [1] and Yang Ping Lee [1]

[1] Geoinformatics, Genomics & Bioinformatics Department, FGV Innovation Centre (Biotechnology), FGV R&D Sdn. Bhd., PT. 23417 Lengkuk Teknologi, Bandar Enstek 71760, Negeri Sembilan, Malaysia; sharmilah.v@fgvholdings.com (S.V.); yangp.lee@fgvholdings.com (Y.P.L.)
[2] Plant Breeding Unit, Tun Razak Agricultural Research Centre (PPPTR), FGV R&D Sdn. Bhd., Jerantut 27000, Pahang, Malaysia; azinuddin.am@fgvholdings.com
* Correspondence: anis.aw@fgvholdings.com; Tel.: +60-12-529-5545

Figure S1 Minimum Inhibitory Concentration (MIC) analysis for *G. boninense* PER71 on 5-fluoroacetic acid (5-FOA) and hygromycin.

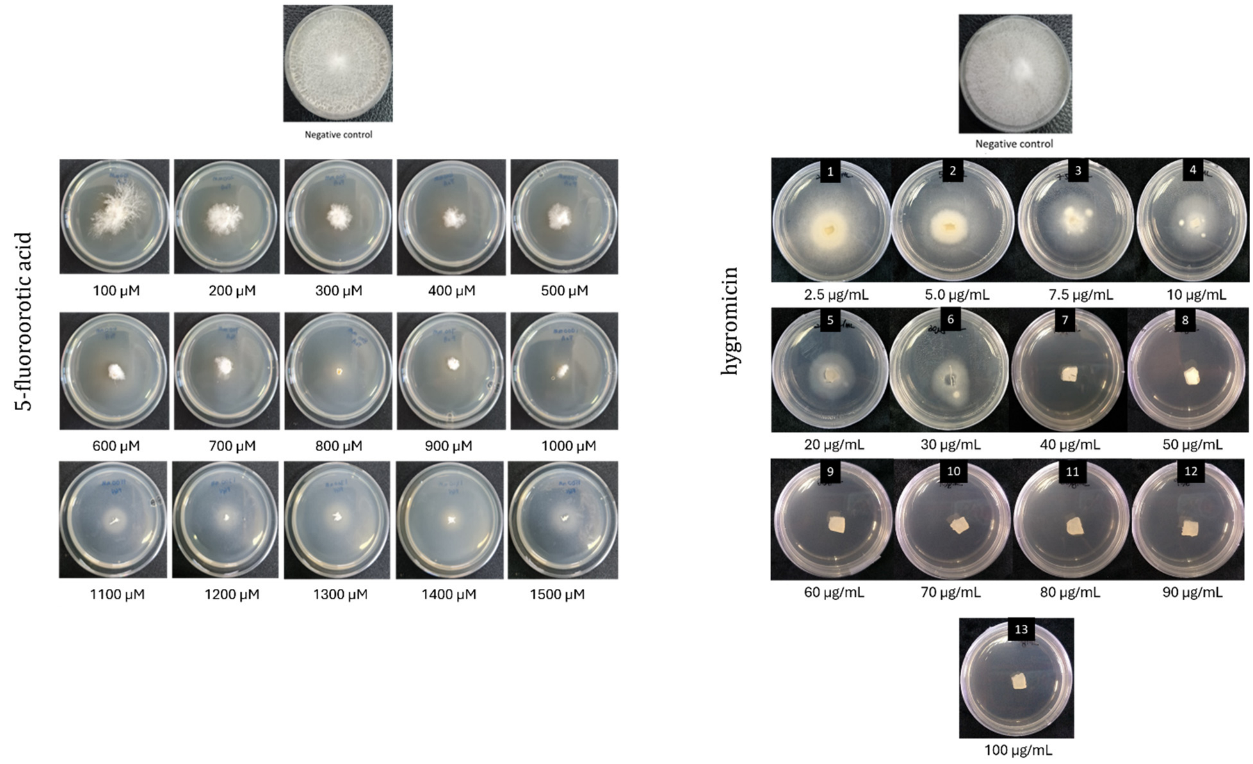

Figure S2 DNA sequences of the *pyrG* gene in *G. boninense* PER71 and alignment of the genomic sequence, transcript, and sgRNA target sites. (A) Full length genomic sequence of the *pyrG* gene; (B) Corresponding transcript (cDNA) sequence; (C) Multiple sequence alignment between the *pyrG* gene and transcript and location of the designed sgRNA (sgRNA1, sgRNA2 and sgRNA3)

Full-length *pyrG* gene and transcript (cDNA) sequences of *Ganoderma boninense* PER71, and alignment of the genomic sequence, transcript, and sgRNA target sites.

A. *pyrG* gene (GenBank submission: SUB16305661)

```
ATGGCATCGGTGGCGAAGGAGACATATGCCCAAAGGGCGGCCAAGCACCCCAATCCAGCAGCAAAAGCCCTCCTCGAGACCATCGAGCGCAAGCGCACAAACTTGTCTGTT
AGCGTCGATGTAACCAAGCAGGAGGACTTCTTCCGAATCATCGACACCGTCGGTCCATATATTTGCCTTGTAAAGGTGGGTGTAGGTCGTCTAAATACCCGTGATCCCGTC
GAATGAATAATTTGAAGACCCACGTAGACATTATCGAGGACTTCGAGCCATCCGTGATCGAGCGCCTCGAAGCACTCAGCCGAAAACATGACTTCCTCATCTTTGAAGACA
GGAAATTTGCGGACATCGGTAGGACATATCTCTGTTCTGTGCTTACCAAATGAGGTCCTGATGAGGGTGTAGGGAACACTGTTGCACTTCAATACTCCGCAGGGGTACACA
AGATCGCGAGTTGGTCGCATATCACGAACGCACACCCCGTCCCCGGCCCGTCCATCATCTCCGGCCTCAGATCCGTTGGTCTCCCTCTTGGCAGAGGCCTTCTGCTCCTGG
CGGAGATGAGTACCAAGGGAAGCCTAGCCACGGGCTCCTACACGGAGGAAGCAGTCCGTATGGCTCGTGCCAACCGCGAGTTCGTGATTGGCTTCATCGCTCAACAACGCA
TGGACGGCGTCGGTCTACGTGAAGGAGAGTCATTGCCTGATGAAGACTTCCTTATCCTCACTCCAGGAGTTGGAATGGACACGAAAGGAGACAGTATGGGACAGCAATACC
GGACACCCAGGGAGGTGATTATGGAGTCTGGTTGCGACGTCATCATCGTTGGGCGGGGTGTTTACGGAAAAGATAACGGTGCAAATGCGGAGGCTGTTCGCGCACAGGCGG
AGAGGTACCGCTCGGAGGGTTGGAAGGCATACCAGGAGAGAGTTGGTATCCATGGACTAA (949 bp)
```

B. Transcript of *pyrG* identified from in house PacBio IsoSeq transcriptomic database

```
ATGGCATCGGTGGCGAAGGAGACATATGCCCAAAGGGCGGCCAAGCACCCGAATCCAGCAGCAAAAGCCCTCCTCGAGACCATCGAGCGCAAGCGCACAAACTTGTCTGTT
AGCGTCGATGTAACCAAGCAGGAGGACTTCTTCCGAATCATCGACACCGTCGGTCCATATATTTGCCTTGGTAAAGACCCACACATTATCGAGGACTTCGAGCCATCCGTG
ATCGAGCGCCTCGAAGCACTCAGCCGAAAACATGACTTCCTCATCTTTGAAGACAGGAAATTTGCGGACATCGGGAACACTGTTGCACTTCAATACTCCGCAGGGGTACAC
AAGATCGCGAGTTGGTCGCATATCACGAACGCACACCCCGTCCCCGGCCCGTCCATCATCTTCGGCCTCAAATCCGTTGGTCTCCCTCTTGGCAGAGGCCTTCTGCTCCTG
GCGGAGATGATGAGTACCAAGGGAAGCCTAGCCACGGGCTCCTACACGGAGGAAGCAGTCCGTATGGCTCGTGCCAACCGCGAGTTCGTGATTGGCTTCATTGCTCAACAA
CGCATGGACGGCGTCGGTCTACGAGAAGGAGAGTCATTGCCTGATGAAGACTTCCTTATCCTCACTCCAGGAGTTGGAATGGACACGAAAGGAGACAGTATGGGACAGCAA
TACCGGACACCCAGGGAGGTGATTATGGAATCTGGTTGCGACGTCATCATCGTTGGGCGGGGTGTTTACGGAAAAGATAACGGCGTAAAATGCGGAGGCTGTTCGCGCACA
GGCGGAGAGGTACCGCTCGGAGGGTTGGAAGGCATTACCAGGAGAGAGTTGGTATCCATGGACTAA (843 bp)
```

C. Multiple sequence alignment between the *pyrG* gene and transcript and location of the designed sgRNA

```
                                                                                            sgpyrG2
             1 ........10........20........30........40........50........60........70........80........90......100
gene|pyrG    1 ATGGCATCGGTGGCGAAGGAGACATATGCCCAAAGGGCGGCCAAGCACCCCAATCCAGCAGCAAAAGCCCTCCTCGAGACCATCGAGCGCAAGCGCACAA
cDNA|pyrG    1 ATGGCATCGGTGGCGAAGGAGACATATGCCCAAAGGGCGGCCAAGCACCCGAATCCAGCAGCAAAAGCCCTCCTCGAGACCATCGAGCGCAAGCGCACAA

           101 ......110.......120.......130.......140.......150.......160.......170.......180.......190.......200
gene|pyrG  101 ACTTGTCTGTTAGCGTCGATGTAACCAAGCAGGAGGACTTCTTCCGAATCATCGACACCGTCGGTCCATATATTTGCCTTGTAAAGGTGGGTGTAGGTCG
cDNA|pyrG  101 ACTTGTCTGTTAGCGTCGATGTAACCAAGCAGGAGGACTTCTTCCGAATCATCGACACCGTCGGTCCATATATTTGCCTTGGTAAAG-------------

                                                                                              sgpyrG3
           201 ......210.......220.......230.......240.......250.......260.......270.......280.......290.......300
gene|pyrG  201 TCTAAATACCCGTGATCCCGTCGAATGAATAATTTGAAGACCCACGTAGACATTATCGAGGACTTCGAGCCATCCGTGATCGAGCGCCTCGAAGCACTCA
cDNA|pyrG  188 -------------------------------------------ACCCACACATTATCGAGGACTTCGAGCCATCCGTGATCGAGCGCCTCGAAGCACTCA

           301 ......310.......320.......330.......340.......350.......360.......370.......380.......390.......400
gene|pyrG  301 GCCGAAAACATGACTTCCTCATCTTTGAAGACAGGAAATTTGCGGACATCGGTAGGACATATCTCTGTTCTGTGCTTACCAAATGAGGTCCTGATGAGGG
cDNA|pyrG  245 GCCGAAAACATGACTTCCTCATCTTTGAAGACAGGAAATTTGCGGACATC--------------------------------------------------

                                             sgpyrG1
           401 ......410.......420.......430.......440.......450.......460.......470.......480.......490.......500
gene|pyrG  401 TGTAGGGAACACTGTTGCACTTCAATACTCCGCAGGGGTACACAAGATCGCGAGTTGGTCGCATATCACGAACGCACACCCCGTCCCCGGCCCGTCCATC
cDNA|pyrG  295 ----GGGAACACTGTTGCACTTCAATACTCCGCAGGGGTACACAAGATCGCGAGTTGGTCGCATATCACGAACGCACACCCCGTCCCCGGCCCGTCCATC

           501 ......510.......520.......530.......540.......550.......560.......570.......580.......590.......600
gene|pyrG  501 ATCTCCGGCCTCAGATCCGTTGGTCTCCCTCTTGGCAGAGGCCTTCTGCTCCTGGCGGA---GATGAGTACCAAGGGAAGCCTAGCCACGGGCTCCTACA
cDNA|pyrG  391 ATCTTCGGCCTCAAATCCGTTGGTCTCCCTCTTGGCAGAGGCCTTCTGCTCCTGGCGGAGATGATGAGTACCAAGGGAAGCCTAGCCACGGGCTCCTACA

           601 ......610.......620.......630.......640.......650.......660.......670.......680.......690.......700
gene|pyrG  598 CGGAGGAAGCAGTCCGTATGGCTCGTGCCAACCGCGAGTTCGTGATTGGCTTCATCGCTCAACAACGCATGGACGGCGTCGGTCTACGTGAAGGAGAGTC
cDNA|pyrG  491 CGGAGGAAGCAGTCCGTATGGCTCGTGCCAACCGCGAGTTCGTGATTGGCTTCATTGCTCAACAACGCATGGACGGCGTCGGTCTACGAGAAGGAGAGTC

           701 ......710.......720.......730.......740.......750.......760.......770.......780.......790.......800
gene|pyrG  698 ATTGCCTGATGAAGACTTCCTTATCCTCACTCCAGGAGTTGGAATGGACACGAAAGGAGACAGTATGGGACAGCAATACCGGACACCCAGGGAGGTGATT
cDNA|pyrG  591 ATTGCCTGATGAAGACTTCCTTATCCTCACTCCAGGAGTTGGAATGGACACGAAAGGAGACAGTATGGGACAGCAATACCGGACACCCAGGGAGGTGATT

           801 ......810.......820.......830.......840.......850.......860.......870.......880.......890.......900
gene|pyrG  798 ATGGAGTCTGGTTGCGACGTCATCATCGTTGGGCGGGGTGTTTACGGAAAAGATAACGGTGC-AAATGCGGAGGCTGTTCGCGCACAGGCGGAGAGGTAC
cDNA|pyrG  691 ATGGAATCTGGTTGCGACGTCATCATCGTTGGGCGGGGTGTTTACGGAAAAGATAACGGCGTAAAATGCGGAGGCTGTTCGCGCACAGGCGGAGAGGTAC

           901 ......910.......920.......930.......940.......950...
gene|pyrG  897 CGCTCGGAGGGTTGGAAGGCA-TACCAGGAGAGAGTTGGTATCCATGGACTAA
cDNA|pyrG  791 CGCTCGGAGGGTTGGAAGGCATTACCAGGAGAGAGTTGGTATCCATGGACTAA
```

Figure S3 Verification of plasmid uptake and *Cas9* expression in protoplast transformants via PCR and RT-PCR analysis. (A) Amplicons of the expected partial *cas9* (~680 bp) sequence were detected using total DNA extraction from transformants. Legend: M: GeneRuler 1 kb DNA Ladder (ThermoFisher Scientific, USA); C: positive control (pGreen0179Glcas9); 1: transformants exhibiting hygromycin resistance on transformation plates containing 110 µg/mL of hygromycin B. **(B)** RT-PCR analysis confirming *cas9* gene expression using cDNA templates synthesized from the first generation of transformants. Amplification of the partial *cas9* gene from cDNA templates confirms active gene expression in the transformants. A thin band was observed, indicating low expression levels. This low abundance is likely driven by the low copy number of the episomal plasmid and a dilution effect across generations. Legend: 1: stock cDNA; 2: 1:5 diluted cDNA, 3: 1:10 diluted cDNA; M: GeneRuler 1 kb DNA Ladder (ThermoFisher Scientific, USA); C: positive control (pGreen0179Glcas9)

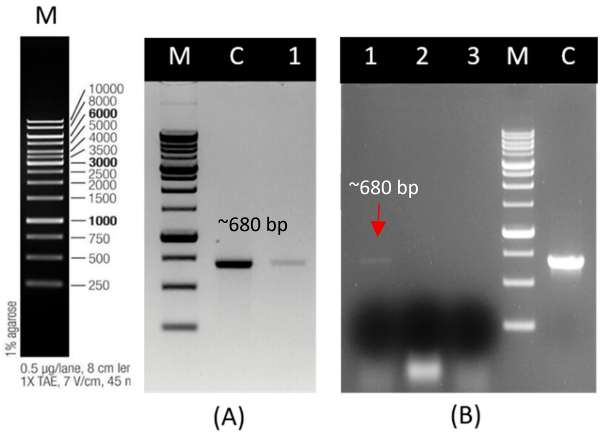

Figure S4 Sequencing analysis of the *pyrG* gene in wild-type (WT) and putative mutant strains. (A) *pyrG* sequencing results from WT and CRISPR/Cas9-edited mutants; (B) Multiple sequence alignment of the *pyrG* gene between WT and mutant ΔpyrG-1; (C) Multiple sequence alignment of the *pyrG* gene between WT and mutant ΔpyrG-2. The alignments highlight the CRISPR/Cas9 target region, including the guide RNA (gRNA) binding site and protospacer-adjacent motif (PAM). Mutant ΔpyrG-1 contains insertions of 662 bp and 277 bp relative to the WT sequence, resulting in a frameshift mutation that disrupts conserved coding motifs.

A. *pyrG* sequencing result for the wild type and putative mutants

i. Gb|pyrGC (Control: wild type)

```
ATGGCATCGGTGGCGAAGGAGACATATGCCCAAAGGGCGGCCAAGCACCCCAATCCAGCAGCAAAAGCCCTCCTCGAGACCATCGAGCGCAAGCGCACAAACTTGTCTGTT
AGCGTCGATGTAACCAAGCAGGAGGACTTCTTCCGAATCATCGACACCGTCGGTCCATATATTTGCCTTGTAAAGGTGGGTGTAGGTCGTCTAAATACCCGTGATCCCGTC
GAATGAATAATTTGAAGACCCACGTAGACATTATCGAGGACTTCGAGCCATCCGTGATCGAGCGCCTCGAAGCACTCAGCCGAAAACATGACTTCCTCATCTTTGAAGACA
GGAAATTTGCGGACATCGGTAGGACATATCTCTGTTCTGTGCTTACCAAATGAGGTCCTGATGAGGGTGTAGGGAACACTGTTGCACTTCAATACTCCGCAGGGGTACACA
AGATCGCGAGTTGGTCGCATATCACGAACGCACACCCCGTCCCCGGCCCGTCCATCATCTCCGGCCTCAGATCCGTTGGTCTCCCTCTTGGCAGAGGCCTTCTGCTCCTGG
CGGAGATGAGTACCAAGGGAAGCCTAGCCACGGGCTCCTACACGGAGGAAGCAGTCCGTATGGCTCGTGCCAACCGCGAGTTCGTGATTGGCTTCATCGCTCAACAACGCA
TGGACGGCGTCGGTCTACGTGAAGGAGAGTCATTGCCTGATGAAGACTTCCTTATCCTCACTCCAGGAGTTGGAATGGACACGAAAGGAGACAGTATGGGACAGCAATACC
GGACACCCAGGGAGGTGATTATGGAGTCTGGTTGCGACGTCATCATCGTTGGGCGGGGTGTTTACGGAAAAGATAACGGTGCAAATGCGGAGGCTGTTCGCGCACAGGCGG
AGAGGTACCGCTCGGAGGGTTGGAAGGCATACCAGGAGAGAGTTGGTATCCATGGACTAA (948 bp)
```

ii. *G. boninense* ΔpyrG-1

```
ATGGCATCGGTGGCGAAGGAGACATATGCCCAAAGGGCGGCCAAGCACCCCAATCCAGCAGCAAAAGCCCTCCTCGAGACCATCGAGCGCAAGCGCACAAACTTGTCTGTT
AGCGTCGAtgtacagtcttcaaccggtcctcggacctatgaatttttggccgccttactatgctcccccatcctcattttccgcgaacgtaagtttacagtccattaggat
gcataccagagtatatgcctgtctaattatgtggtgagcagagggcagcttaggagagatcgaggtccatcttgacaaaaatcccttatcgcgacttcctcagtcctcggg
ccacccgtatttggtacgaaattgatctaaccgacgctagccgagcactcgattaagtatttcgggcttattagcgccgtctggtggcccaaggaccaccgtccgaggcat
attcaacgcattgtgttcgtccccgatggcaattcccagtatttaggacacaaaatggttggcatgaacatgttcatgcttcatcataaatttgggactacgtggtcgaga
acatagtgctagatctgccctccaaaattcattttctcgctgttttactgtaacaatcgccttagaacgtgctatttcgcccctcccgccctcagtttttttgagtggttt
ggtgaaacaggttctgatgcatgagagacaattttgtggcaagagaaacagggggagctcgcaaagggtgaagtggcccgaggacccatggtggcccgaggaccggttaa
agacTGTAACCAAGCAGGAGGACTTCTTCCGAATCATCGACACCGTCGGTCCATATATTTGCCTTGTAAAGGTGGGTGTAGGTCGTCTAAATACCCGTGATCCCGTCGAAT
GAATAATTTGAAGACCCACGTAGACATTATCGAGGACTTCGAGCCATCCGTGATCGAGCGCCTCGAAGCACTCAGCCGAAAACATGACTTCCTCATCTTTGAAGACAGGAA
ATTTGCGGACATCGGTAGGACATATCTCTGTTCTGTGCTTACCAAATGAGGTCCTGATGAGGGTGTAGGGAACACTGTTGCACTTCAATACTCCGCAGGGGTACACAAGAT
CGCGAGTTGGTCGCATATCACGAACGCACACCCCGTCCCCGGCCCGTCCATCATCTCCGGCCTCAGATCCGTTGGTCTCCCTCTTGGCAGAGGCCTTCTGCTCCTGGCGGA
GATGAGTACCAAGGGAAGCCTAGCCACGGGCTCCTACACGGAGGAAGCAGTCCGTATGGCTCGTGCCAACCGCGAGTTCGTGATTGGCTTCATCGCTCAACAACGCATGGA
CGGCGTCGGTCTACGTGAAGGAGAGTCATTGCCTGATGAAGACTTCCTTATCCTCACTCCAGGAGTTGGAATGGACACGAAAGGAGACAGTATGGGACAGCAATACCGGAC
ACCCAGGGAGGTGATTATGGAGTCTGGTTGCGACGTCATCATCGTTGGGCGGGGTGTTTACGGAAAAGATAACGGTGCAAATGCGGAGGCTGTTCGCGCACAGGCGGAGAG
GTACCGCTCGGAGGGTTGGAAGGCATACCAGGAGAGAGTTGGTATCCATGGACTAA
(1611 bp)
```

iii. *G. boninense* ΔpyrG-2

```
ATGGCATCGGTGGCGAAGGAGACATATGCCCAAAGGGCGGCCAAGCACCCCAATCCAGCAGCAAAAGCCCTCCTCGAGACCATCGAGCGCAAGCGCACAAACTTGTCTGTT
AGCGTCGATGTAACCAAGCAGGAGGACTTCTTCCGAATCATCGACACCGTCGGTCCATATATTTGCCTTGTAAAGGTGGGTGTAGGTCGTCTAAATACCCGTGATCCCGTC
GAATGAATAATTTGAAGACCCACGTAGACATTATCGAGGACTTCGAGCCATCCGTGATCGAGCGCCTCGAAGCACTCAGCCGAAAACATGACTTCCTCATCTTTGAAGACA
GGAAATTTGCGGACATCGGTAGGACATATCTCTGTTCTGTGCTTACCAAATGAGGTCCTGATGAGGGTGTAGGGAACACTGTTGCACTTCAATACTCCGCAGGGGTACACA
AGATCGCGAGTTGGTCGCATATCACGAACGCCcccgaggccagatgacccatacacgccaccacacaatggtgtcaccgaggaatacctgtgattttgttagctatactgg
ccctagcgcatcgtataatgtagtacacgcacattgagggtgaggatgatggtcggtgcacagacggcgagaccgtgcgctgcaccaggaaggccgaagccagacaacgag
caggatggggtacccggggagatggctacgatcggaactgcctcatctgttgtgcattggcactgcaactgggccgcgtacgctttaCACCCCGTCCCCGGCCCGTCCATC
ATCTCCGGCCTCAGATCCGTTGGTCTCCCTCTTGGCAGAGGCCTTCTGCTCCTGGCGGAGATGAGTACCAAGGGAAGCCTAGCCACGGGCTCCTACACGGAGGAAGCAGTC
CGTATGGCTCGTGCCAACCGCGAGTTCGTGATTGGCTTCATCGCTCAACAACGCATGGACGGCGTCGGTCTACGTGAAGGAGAGTCATTGCCTGATGAAGACTTCCTTATC
CTCACTCCAGGAGTTGGAATGGACACGAAAGGAGACAGTATGGGACAGCAATACCGGACACCCAGGGAGGTGATTATGGAGTCTGGTTGCGACGTCATCATCGTTGGGCGG
GGTGTTTACGGAAAAGATAACGGTGCAAATGCGGAGGCTGTTCGCGCACAGGCGGAGAGGTACCGCTCGGAGGGTTGGAAGGCATACCAGGAGAGAGTTGGTATCCATGGA
CTAA (1226 bp)
```

B. Multiple sequence alignment (MSA) between wild-type *pyrG* and mutant, ΔpyrG-1.

```
                1 ........10........20........30........40........50........60........70........80
ΔpyrG-1         1 ATGGCATCGGTGGCGAAGGAGACATATGCCCAAAGGGCGGCCAAGCACCCCAATCCAGCAGCAAAAGCCCTCCTCGAGAC
Gb|pyrGC        1 ATGGCATCGGTGGCGAAGGAGACATATGCCCAAAGGGCGGCCAAGCACCCCAATCCAGCAGCAAAAGCCCTCCTCGAGAC
consensus       1 ATGGCATCGGTGGCGAAGGAGACATATGCCCAAAGGGCGGCCAAGCACCCCAATCCAGCAGCAAAAGCCCTCCTCGAGAC

                       sgpyrG2
               81 ........90.......100.......110.......120.......130.......140.......150.......160
ΔpyrG-1        81 CATCGAGCGCAAGCGCACAAACTTGTCTGTTAGCGTCGATGTACAGTCTTCAACCGGTCCTCGGACCTATGAATTTTTGG
Gb|pyrGC       81 CATCGAGCGCAAGCGCACAAACTTGTCTGTTAGCGTCGA-----------------------------------------
consensus      81 CATCGAGCGCAAGCGCACAAACTTGTCTGTTAGCGTCGAtgtacagtcttcaaccggtcctcggacctatgaattttttgg

              161 .......170.......180.......190.......200.......210.......220.......230.......240
M3-pyrG-SH    161 CCGCCTTACTATGCTCCCCCATCCTCATTTTCCGCGAACGTAAGTTTACAGTCCATTAGGATGCATACCAGAGTATATGC
Gb|pyrGC      120 --------------------------------------------------------------------------------
consensus     161 ccgccttactatgctcccccatcctcattttccgcgaacgtaagtttacagtccattaggatgcataccagagtatatgc
              241 .......250.......260.......270.......280.......290.......300.......310.......320
ΔpyrG-1       241 CTGTCTAATTATGTGGTGAGCAGAGGGCAGCTTAGGAGAGATCGAGGTCCATCTTGACAAAAATCCCTTATCGCGACTTC
Gb|pyrGC      120 --------------------------------------------------------------------------------
consensus     241 ctgtctaattatgtggtgagcagagggcagcttaggagagatcgaggtccatcttgacaaaaatcccttatcgcgacttc

              321 .......330.......340.......350.......360.......370.......380.......390.......400
ΔpyrG-1       321 CTCAGTCCTCGGGCCACCCGTATTTGGTACGAAATTGATCTAACCGACGCTAGCCGAGCACTCGATTAAGTATTTCGGGC
Gb|pyrGC      120 --------------------------------------------------------------------------------
consensus     321 ctcagtcctcgggccacccgtatttggtacgaaattgatctaaccgacgctagccgagcactcgattaagtatttcgggc

              401 .......410.......420.......430.......440.......450.......460.......470.......480
ΔpyrG-1       401 TTATTAGCGCCGTCTGGTGGCCCAAGGACCACCGTCCGAGGCATATTCAACGCATTGTGTTCGTCCCCGATGGCAATTCC
Gb|pyrGC      120 --------------------------------------------------------------------------------
consensus     401 ttattagcgccgtctggtggcccaaggaccaccgtccgaggcatattcaacgcattgtgttcgtccccgatggcaattcc

              481 .......490.......500.......510.......520.......530.......540.......550.......560
ΔpyrG-1       481 CAGTATTTAGGACACAAAATGGTTGGCATGAACATGTTCATGCTTCATCATAAATTTGGGACTACGTGGTCGAGAACATA
Gb|pyrGC      120 --------------------------------------------------------------------------------
consensus     481 cagtatttaggacacaaaatggttggcatgaacatgttcatgcttcatcataaatttgggactacgtggtcgagaacata

              561 .......570.......580.......590.......600.......610.......620.......630.......640
ΔpyrG-1       561 GTGCTAGATCTGCCCTCCAAAATTCATTTTCTCGCTGTTTTACTGTAACAATCGCCTTAGAACGTGCTATTTCGCCCCTC
pyrGC_SP6|    120 --------------------------------------------------------------------------------
consensus     561 gtgctagatctgccctccaaaattcattttctcgctgttttactgtaacaatcgccttagaacgtgctatttcgcccctc

              641 .......650.......660.......670.......680.......690.......700.......710.......720
ΔpyrG-1       641 CCGCCCTCAGTTTTTTTGAGTGGTTTGGTGAAACAGGTTCTGATGCATGAGAGACAATTTTGTGGCAAGAGAAACAGGGG
Gb|pyrGC      120 --------------------------------------------------------------------------------
consensus     641 ccgccctcagtttttttgagtggtttggtgaaacaggttctgatgcatgagagacaattttgtggcaagagaaacagggg

              721 .......730.......740.......750.......760.......770.......780.......790.......800
M3-pyrG-SH    721 GGAGCTCGCAAAGGGTGAAGTGGCCCGAGGACCCATGGTGGCCCGAGGACCGGTTAAAGACTGTAACCAAGCAGGAGGAC
Gb|pyrGC      120 -------------------------------------------------------------TGTAACCAAGCAGGAGGAC
consensus     721 ggagctcgcaaagggtgaagtggcccgaggacccatggtggcccgaggaccggttaaagacTGTAACCAAGCAGGAGGAC

              801 .......810.......820.......830.......840.......850.......860.......870.......880
ΔpyrG-1       801 TTCTTCCGAATCATCGACACCGTCGGTCCATATATTTGCCTTGTAAAGGTGGGTGTAGGTCGTCTAAATACCCGTGATCC
Gb|pyrGC      139 TTCTTCCGAATCATCGACACCGTCGGTCCATATATTTGCCTTGTAAAGGTGGGTGTAGGTCGTCTAAATACCCGTGATCC
consensus     801 TTCTTCCGAATCATCGACACCGTCGGTCCATATATTTGCCTTGTAAAGGTGGGTGTAGGTCGTCTAAATACCCGTGATCC

                                                                                       sgpyrG3
              881 .......890.......900.......910.......920.......930.......940.......950.......960
ΔpyrG-1       881 CGTCGAATGAATAATTTGAAGACCCACGTAGACATTATCGAGGACTTCGAGCCATCCGTGATCGAGCGCCTCGAAGCACT
Gb|pyrGC      219 CGTCGAATTAATAATTTGAAGACCCACGTAGACATTATCGAGGACTTCGAGCCATCCGTGATCGAGCGCCTCGAAGCACT
consensus     881 CGTCGAATgAATAATTTGAAGACCCACGTAGACATTATCGAGGACTTCGAGCCATCCGTGATCGAGCGCCTCGAAGCACT

              961 .......970.......980.......990......1000......1010......1020......1030......1040
ΔpyrG-1       961 CAGCCGAAAACATGACTTCCTCATCTTTGAAGACAGGAAATTTGCGGACATCGGTAGGACATATCTCTGTTCTGTGCTTA
Gb|pyrGC      299 CAGCCGAAAACATGACTTCCTCATCTTTGAAGACAGGAAATTTGCGGACATCGGTAGGACATATCTCTGTTCTGTGCTTA
consensus     961 CAGCCGAAAACATGACTTCCTCATCTTTGAAGACaGGAAATTTGCGGACATCGGTAGGACATATCTCTGTTCTGTGCTTA

                                                                         sgpyrG1
             1041 ......1050......1060......1070......1080......1090......1100......1110......1120
ΔpyrG-1      1041 CCAAATGAGGTCCTGATGAGGGTGTAGGGAACACTGTTGCACTTCAATACTCCGCAGGGGTACACAAGATCGCGAGTTGG
Gb|pyrGC      379 CCAAATGAGGTCCTGATGAGGGTGTAGGGAACACTGTTGCACTTCAATACTCCGCAGGGGTACACAAGATCGCGAGTTGG
consensus    1041 CCAAATGAGGTCCTGATGAGGGTGTAGGGAACACTGTTGCACTTCAATACTCCGCAGGGGTACACAAGATCGCGAGTTGG

             1121 ......1130......1140......1150......1160......1170......1180......1190......1200
ΔpyrG-1      1121 TCGCATATCACGAACGCACACCCCGTCCCCGGCCCGTCCATCATCTCCGGCCTCAGATCCGTTGGTCTCCCTCTTGGCAG
Gb|pyrGC      459 TCGCATATCACGAACGCACACCCCGTCCCCGGCCCGTCCATCATCTCCGGCCTCAGATCCGTTGGTCTCCCTCTTGGCAG
consensus    1121 TCGCATAtCACGAACGCACACCCCGTCCCCGGCCCGTCCATCATCTCCGGCCTCAGATCCGTTGGTCTcCCTCTTGGCaG

             1201 ......1210......1220......1230......1240......1250......1260......1270......1280
ΔpyrG-1      1201 AGGCCTTCTGCTCCTGGCGGAGATGAGTACCAAGGGAAGCCTAGCCACGGGCTCCTACACGGAGGAAGCAGTCCGTATGG
Gb|pyrGC      539 AGGCCTTCTGCTCCTGGCGGAGATGAGTACCAAGGGAAGCCTAGCCACGGGCTCCTACACGGAGGAAGCAGTCCGTATGG
consensus    1201 AGGCCTTCTGCTCCTGGCGGAGATGAGTACCAAGGGAAGCCTAGCCACGGGCTCCTACACGGAGGAAGCAGTCCGTATGG
```

```
          1281 ......1290......1300......1310......1320......1330......1340......1350......1360
ΔpyrG-1   1281 CTCGTGCCAACCGCGAGTTCGTGATTGGCTTCATCGCTCAACAACGCATGGACGGCGTCGGTCTACGTGAAGGAGAGTCA
Gb|pyrGC   619 CTCGTGCCAACCGCGAGTTCGTGATTGGCTTCATCGCTCAACAACGCATGGACGGCGTCGGTCTACGTGAAGGAGAGTCA
consensus 1281 CTCGTGCCAACCGCGAGTTCGTGATTGGCTTCATCGCTCAACAACGCATGGACGGCGTCGGTCTACGTGAAGGAGAGTCA

          1361 ......1370......1380......1390......1400......1410......1420......1430......1440
ΔpyrG-1   1361 TTGCCTGATGAAGACTTCCTTATCCTCACTCCAGGAGTTGGAATGGACACGAAAGGAGACAGTATGGGACAGCAATACCG
Gb|pyrGC   699 TTGCCTGATGAAGACTTCCTTATCCTCACTCCAGGAGTTGGAATGGACACGAAAGGAGACAGTATGGGACAGCAATACCG
consensus 1361 TTGCCTGATGAAGACTTCCTTATCCTCACTCCAGGAGTTGGAATGGACACGAAAGGAGACAGTATGGGACAGCAATACCG

          1441 ......1450......1460......1470......1480......1490......1500......1510......1520
ΔpyrG-1   1441 GACACCCAGGGAGGTGATTATGGAGTCTGGTTGCGACGTCATCATCGTTGGGCGGGGTGTTTACGGAAAAGATAACGGTG
Gb|pyrGC   779 GACACCCAGGGAGGTGATTATGGAGTCTGGTTGCGACGTCATCATCGTTGGGCGGGGTGTTTACGGAAAAGATAACGGTG
consensus 1441 GACACCCAGGGAGGTGATTATGGAGTCTGGTTGCGACGTCATCATCGTTGGGCGGGGTGTTTACGGAAAAGATAACGGTG

          1521 ......1530......1540......1550......1560......1570......1580......1590......1600
ΔpyrG-1   1521 CAAATGCGGAGGCTGTTCGCGCACAGGCGGAGAGGTACCGCTCGGAGGGTTGGAAGGCATACCAGGAGAGAGTTGGTATC
Gb|pyrGC  859  CAAATGCGGAGGCTGTTCGCGCACAGGCGGAGAGGTACCGCTCGGAGGGTTGGAAGGCATACCAGGAGAGAGTTGGTATC
consensus 1521 CAAATGCGGAGGCTGTTCGCGCACAGGCGGAGAGGTACCGCTCGGAGGGTTGGAAGGCATACCAGGAGAGAGTTGGTATC

          1601 ......1610
ΔpyrG-1   1601 CATGGACTAA
Gb|pyrGC   939 CATGGACTAA
consensus 1601 CATGGACTAA
```

C. BLAST analysis on the inserted sequence revealed up to 98.94% sequence similarity with the sequence located at LG3, LG4 and 91-94% sequence similarity with LG2 and LG10.

	Description	Max Score	Total Score	Query Cover	E value	Per. Ident	Acc. Len	Accessio
☑	Ganoderma boninense strain G3 linkage group LG3	1182	2353	100%	0.0	98.94%	4640194	CM035306.1
☑	Ganoderma boninense strain G3 linkage group LG3	1182	2353	100%	0.0	98.94%	4640194	PJEW03000003.1
☑	Ganoderma boninense strain G3 linkage group LG4	1175	1175	100%	0.0	98.79%	4597913	CM035307.1
☑	Ganoderma boninense strain G3 linkage group LG4	1175	1175	100%	0.0	98.79%	4597913	PJEW03000004.1
☑	Ganoderma boninense strain G3 linkage group LG2	977	977	100%	0.0	94.10%	5458015	CM035305.1
☑	Ganoderma boninense strain G3 linkage group LG2	977	977	100%	0.0	94.10%	5458015	PJEW03000002.1
☑	Ganoderma boninense strain G3 linkage group LG10	896	1793	100%	0.0	91.21%	3696312	CM035313.1
☑	Ganoderma boninense strain G3 linkage group LG10	896	1793	100%	0.0	91.21%	3696312	PJEW03000010.1

D. Multiple sequence alignment (MSA) between wild-type *pyrG* and mutant, ΔpyrG-2.

```
Gb|pyrGC     ATGGCATCGGTGGCGAAGGAGACATATGCCCAAAGGGCGGCCAAGCACCCCAATCCAGCA    60
ΔpyrG-2      ATGGCATCGGTGGCGAAGGAGACATATGCCCAAAGGGCGGCCAAGCACCCCAATCCAGCA    60
             ************************************************************

                                         sgpyrG1
Gb|pyrGC     GCAAAAGCCCTCCTCGAGACCATCGAGCGCAAGCGCACAAACTTGTCTGTTAGCGTCGAT    120
ΔpyrG-2      GCAAAAGCCCTCCTCGAGACCATCGAGCGCAAGCGCACAAACTTGTCTGTTAGCGTCGAT    120
             ************************************************************

Gb|pyrGC     GTAACCAAGCAGGAGGACTTCTTCCGAATCATCGACACCGTCGGTCCATATATTTGCCTT    180
ΔpyrG-2      GTAACCAAGCAGGAGGACTTCTTCCGAATCATCGACACCGTCGGTCCATATATTTGCCTT    180
             ************************************************************

Gb|pyrGC     GTAAAGGTGGGTGTAGGTCGTCTAAATACCCGTGATCCCGTCGAATGAATAATTTGAAGA    240
ΔpyrG-2      GTAAAGGTGGGTGTAGGTCGTCTAAATACCCGTGATCCCGTCGAATGAATAATTTGAAGA    240
             ************************************************************

                                                             sgpyrG3
Gb|pyrGC     CCCACGTAGACATTATCGAGGACTTCGAGCCATCCGTGATCGAGCGCCTCGAAGCACTCA    300
ΔpyrG-2      CCCACGTAGACATTATCGAGGACTTCGAGCCATCCGTGATCGAGCGCCTCGAAGCACTCA    300
             ************************************************************

Gb|pyrGC     GCCGAAAACATGACTTCCTCATCTTTGAAGACAGGAAATTTGCGGACATCGGTAGGACAT    360
ΔpyrG-2      GCCGAAAACATGACTTCCTCATCTTTGAAGACAGGAAATTTGCGGACATCGGTAGGACAT    360
             ************************************************************
```

```
Gb|pyrGC      ATCTCTGTTCTGTGCTTACCAAATGAGGTCCTGATGAGGGTGTAGGGAACACTGTTGCAC   420
ΔpyrG-2       ATCTCTGTTCTGTGCTTACCAAATGAGGTCCTGATGAGGGTGTAGGGAACACTGTTGCAC   420
              ************************************************************

                                  sgpyrG1
Gb|pyrGC      TTCAATACTCCGCAGGGGTACACAAGATCGCGAGTTGGTCGCATATCACGAACGCA----   476
ΔpyrG-2       TTCAATACTCCGCAGGGGTACACAAGATCGCGAGTTGGTCGCATATCACGAACGCCccga   480
              *******************************************************

Gb|pyrGC      ------------------------------------------------------------   476
ΔpyrG-2       ggccagatgacccatacacgccaccacacaatggtgtcaccgaggaatacctgtgatttt   540

Gb|pyrGC      ------------------------------------------------------------   476
ΔpyrG-2       tgttagctatactggccctagcgcatcgtataatgtagtacacgcacattgagggtgagg   600

Gb|pyrGC      ------------------------------------------------------------   476
ΔpyrG-2       atgatggtcggtgcacagacggcgagaccgtgcgctgcaccaggaaggccgaagccagac   660

Gb|pyrGC      ------------------------------------------------------------   476
ΔpyrG-2       aacgagcaggatggggtacccggggagatggctacgatcggaactgcctcatctgttgtg   720

Gb|pyrGC      ---------------------------------CACCCCGTCCCCGGCCCGTCCATCATC   503
ΔpyrG-2       cattggcactgcaactgggccgcgtacgctttaCACCCCGTCCCCGGCCCGTCCATCATC   780
                                               ***************************

Gb|pyrGC      TCCGGCCTCAGATCCGTTGGTCTCCCTCTTGGCAGAGGCCTTCTGCTCCTGGCGGAGATG   563
ΔpyrG-2       TCCGGCCTCAGATCCGTTGGTCTCCCTCTTGGCAGAGGCCTTCTGCTCCTGGCGGAGATG   840
              ************************************************************

Gb|pyrGC      AGTACCAAGGGAAGCCTAGCCACGGGCTCCTACACGGAGGAAGCAGTCCGTATGGCTCGT   623
ΔpyrG-2       AGTACCAAGGGAAGCCTAGCCACGGGCTCCTACACGGAGGAAGCAGTCCGTATGGCTCGT   900
              ************************************************************

Gb|pyrGC      GCCAACCGCGAGTTCGTGATTGGCTTCATCGCTCAACAACGCATGGACGGCGTCGGTCTA   683
ΔpyrG-2       GCCAACCGCGAGTTCGTGATTGGCTTCATCGCTCAACAACGCATGGACGGCGTCGGTCTA   960
              ************************************************************

Gb|pyrGC      CGTGAAGGAGAGTCATTGCCTGATGAAGACTTCCTTATCCTCACTCCAGGAGTTGGAATG   743
ΔpyrG-2       CGTGAAGGAGAGTCATTGCCTGATGAAGACTTCCTTATCCTCACTCCAGGAGTTGGAATG   1020
              ************************************************************

Gb|pyrGC      GACACGAAAGGAGACAGTATGGGACAGCAATACCGGACACCCAGGGAGGTGATTATGGAG   803
ΔpyrG-2       GACACGAAAGGAGACAGTATGGGACAGCAATACCGGACACCCAGGGAGGTGATTATGGAG   1080
              ************************************************************

Gb|pyrGC      TCTGGTTGCGACGTCATCATCGTTGGGCGGGGTGTTTACGGAAAAGATAACGGTGCAAAT   863
ΔpyrG-2       TCTGGTTGCGACGTCATCATCGTTGGGCGGGGTGTTTACGGAAAAGATAACGGTGCAAAT   1140
              ************************************************************

Gb|pyrGC      GCGGAGGCTGTTCGCGCACAGGCGGAGAGGTACCGCTCGGAGGGTTGGAAGGCATTACCA   923
ΔpyrG-2       GCGGAGGCTGTTCGCGCACAGGCGGAGAGGTACCGCTCGGAGGGTTGGAAGGCATTACCA   1200
              ************************************************************

Gb|pyrGC      GGAGAGAGTTGGTATCCATGGACTAA   949
ΔpyrG-2       GGAGAGAGTTGGTATCCATGGACTAA   1226
              **************************
```

E. Further BLASTN analysis of the 277 bp extra sequence inserted in *pyrG* gene in ΔpyrG-2 revealed a strong match to scaffold LG8 of the *G. boninense* G3 genome, with 98% coverage and 99.64% sequence identity, indicating that the insertion likely originated from an internal genomic fragment rather than exogenous contamination.

	Description	Max Score	Total Score	Query Cover	E value	Per. Ident	Acc. Len	Accession
☑	Ganoderma boninense strain G3 linkage group LG8, whole genome shotgun sequence	501	501	98%	1e-141	99.64%	3918320	CM035311.1
☑	Ganoderma boninense strain G3 linkage group LG8, whole genome shotgun sequence	501	501	98%	1e-141	99.64%	3918320	PJEW03000008.1

Download GenBank Graphics

Ganoderma boninense strain G3 linkage group LG8, whole genome shotgun sequence

Sequence ID: CM035311.1 **Length:** 3918320 **Number of Matches:** 1

Range 1: 1229232 to 1229505 GenBank Graphics ▼ Next Match ▲ Previous Match

Score	Expect	Identities	Gaps	Strand
501 bits(271)	1e-141	273/274(99%)	0/274(0%)	Plus/Plus

```
Query  2        CGAGGCCAGATGACCCATACACGCCACCACACAATGGTGTCACCGAGGAATACCTGTGAT  61
                ||||||||||||||||||||||||||||||||||||||||||||||||||||||||||||
Sbjct  1229232  CGAGGCCAGATGACCCATACACGCCACCACACAATGGTGTCACCGAGGAATACCTGTGAT  1229291

Query  62       TTTTGTTAGCTATACTGGCCCTAGCGCATCGTATAATGTAGTACACGCACattgagggtg  121
                ||||||||||||||||||||||||||||||||||||||||||||||||||||||||||||
Sbjct  1229292  TTTTGTTAGCTATACTGGCCCTAGCGCATCGTATAATGTAGTACACGCACATTGAGGGTG  1229351

Query  122      aggatgatGGTCGGTGCACAGACGGCGAGACCGTGCGCTGCACCAGGAAGGCCGAAGCCA  181
                ||||||||||||||||||||||||||||||||||||||||||||||||||||||||||||
Sbjct  1229352  AGGATGATGGTCGGTGCACAGACGGCGAGACCGTGCGCTGCACCAGGAAGGCCGAAGCCA  1229411

Query  182      GACAACGAGCAGGATGGGGTACCCGGGGAGGTGGCTACGATCGGAACTGCCTCATCTGTT  241
                |||||||||||||||||||||||||||||| |||||||||||||||||||||||||||||
Sbjct  1229412  GACAACGAGCAGGATGGGGTACCCGGGGAGATGGCTACGATCGGAACTGCCTCATCTGTT  1229471

Query  242      GTGCATTGGCACTGCAACTGGGCCGCGTACGCTT  275
                ||||||||||||||||||||||||||||||||||
Sbjct  1229472  GTGCATTGGCACTGCAACTGGGCCGCGTACGCTT  1229505
```

Download GenBank Graphics

Ganoderma boninense strain G3 linkage group LG8, whole genome shotgun sequence

Sequence ID: PJEW03000008.1 **Length:** 3918320 **Number of Matches:** 1

Range 1: 1229232 to 1229505 GenBank Graphics ▼ Next Match ▲ Previous Match

Score	Expect	Identities	Gaps	Strand
501 bits(271)	1e-141	273/274(99%)	0/274(0%)	Plus/Plus

```
Query  2        CGAGGCCAGATGACCCATACACGCCACCACACAATGGTGTCACCGAGGAATACCTGTGAT  61
                ||||||||||||||||||||||||||||||||||||||||||||||||||||||||||||
Sbjct  1229232  CGAGGCCAGATGACCCATACACGCCACCACACAATGGTGTCACCGAGGAATACCTGTGAT  1229291

Query  62       TTTTGTTAGCTATACTGGCCCTAGCGCATCGTATAATGTAGTACACGCACattgagggtg  121
                ||||||||||||||||||||||||||||||||||||||||||||||||||||||||||||
Sbjct  1229292  TTTTGTTAGCTATACTGGCCCTAGCGCATCGTATAATGTAGTACACGCACATTGAGGGTG  1229351

Query  122      aggatgatGGTCGGTGCACAGACGGCGAGACCGTGCGCTGCACCAGGAAGGCCGAAGCCA  181
                ||||||||||||||||||||||||||||||||||||||||||||||||||||||||||||
Sbjct  1229352  AGGATGATGGTCGGTGCACAGACGGCGAGACCGTGCGCTGCACCAGGAAGGCCGAAGCCA  1229411

Query  182      GACAACGAGCAGGATGGGGTACCCGGGGAGGTGGCTACGATCGGAACTGCCTCATCTGTT  241
                |||||||||||||||||||||||||||||| |||||||||||||||||||||||||||||
Sbjct  1229412  GACAACGAGCAGGATGGGGTACCCGGGGAGATGGCTACGATCGGAACTGCCTCATCTGTT  1229471

Query  242      GTGCATTGGCACTGCAACTGGGCCGCGTACGCTT  275
                ||||||||||||||||||||||||||||||||||
Sbjct  1229472  GTGCATTGGCACTGCAACTGGGCCGCGTACGCTT  1229505
```

Figure S5 DNA sequences of the *hyd-2* gene in *G. boninense* PER71 and alignment of the genomic sequence, transcript, and sgRNA target sites. (A) Full length genomic sequence of the *hyd-2* gene; (B) Corresponding transcript (cDNA) sequence; (C) Multiple sequence alignment between the *hyd-2* gene and transcript and location of the designed sgRNA (sgRNA1, sgRNA2 and sgRNA3)

A. Full *hydrophobin-2* sequence for *G. boninense* PER71 (GenBank submission: PZ661906)

```
>Gene|Hyd-2
ATGTTCTCTCGCGCCGTCGCCTTCACTACCCTCGCTCTGCCCCTCCTTGCGGTCGCCACTCCCGTCGAGCTGGTGGCTCGCCAGAGCTGCAGCACCGGAGCGGTGCAGTGC
TGCAACAGCACCGTCGAGGTAAGGCATCTCGTCGACAACGTTAACGGAACCATCTGCTCACGCGCCTCTTACCCTCCCACTCGCAGGCCAACTCGGCGTCGGCCAACTTGC
TCCTTGGTCTGCTTGGCATTGTCCTCGGGGACATCACTGGCCTCATTGGCCTGAACTGCTCGCCCCTCACCGTCATCGGTGTTGGCTCCGGCAACGCCTGCTCCGCCCAGG
CTGTCTGCTGCACCAACAACAACGTCGTAAGTCTCTCAGAAGGCGCGCCATTCGGAAGACCCTGAACGTACTAACTGTGCCTATCACTCCAGGGTGGTTTGATCTCCATTG
GCTGCGTCCCCGTCACGCTCTAA
```

B. Transcript of *hyd-2* identified from in house PacBio IsoSeq transcriptomic database

```
>cDNA|Hyd-2
ATGTTCTCTCGCGCCGTCGCCTTCACTACCCTCGCTCTGCCCCTCCTTGCGGTCGCCACTCCCGTCGAGCTGGTGGCTCGCCAGAGCTGCAGCACCGGAGCGGTGCAGTGC
TGCAACAGCACCGTCGAGGCCAACTCGGCGTCGGCCAACTTGCTCCTTGGTCTGCTTGGCATTGTCCTCGGGGACATCACTGGCCTCATTGGCCTGAACTGCTCGCCCCTC
ACCGTCATCGGTGTTGGCTCCGGCAACGCCTGCTCCGCCCAGGCTGTCTGCTGCACCAACAACAACGTCGGTGGTTTGATCTCCATTGGCTGCGTCCCCGTCACGCTCTAA
```

C. Multiple sequence alignment between the *hydrophobin-2* gene, transcript and location of the designed sgRNA (sgRNA1, sgRNA2 and sgRNA3). Green highlighted sequence = Intron

```
                                                 sgpyrG1
Gene|Hyd-2 ATGTTCTCTCGCGCCGTCGCCTTCACTACCCTCGCTCTGCCCCTCCTTGCGGTCGCCACT
cDNA|Hyd-2 atgttctctcgcgccgtcgccttcactaccctcgctctgcccctccttgcggtcgccact
           ************************************************************
                                                              sgpyrG2
Gene|Hyd-2 CCCGTCGAGCTGGTGGCTCGCCAGAGCTGCAGCACCGGAGCGGTGCAGTGCTGCAACAGC
cDNA|Hyd-2 cccgtcgagctggtggctcgccagagctgcagcaccggagcggtgcagtgctgcaacagc
           ************************************************************

Gene|Hyd-2 ACCGTCGAGGTAAGGCATCTCGTCGACAACGTTAACGGAACCATCTGCTCACGCGCCTCT
cDNA|Hyd-2 accgtcg-----------------------------------------------------
           *******

Gene|Hyd-2 TACCCTCCCACTCGCAGGCCAACTCGGCGTCGGCCAACTTGCTCCTTGGTCTGCTTGGCA
cDNA|Hyd-2 ---------------aggccaactcggcgtcggccaacttgctccttggtctgcttggca
                          *********************************************
                 sgpyrG3
Gene|Hyd-2 TTGTCCTCGGGGACATCACTGGCCTCATTGGCCTGAACTGCTCGCCCCTCACCGTCATCG
cDNA|Hyd-2 ttgtcctcggggacatcactggcctcattggcctgaactgctcgcccctcaccgtcatcg
           ************************************************************

Gene|Hyd-2 GTGTTGGCTCCGGCAACGCCTGCTCCGCCCAGGCTGTCTGCTGCACCAACAACAACGTCG
cDNA|Hyd-2 gtgttggctccggcaacgcctgctccgcccaggctgtctgctgcaccaacaacaacgtc-
           ***********************************************************

Gene|Hyd-2 TAAGTCTCTCAGAAGGCGCGCCATTCGGAAGACCCTGAACGTACTAACTGTGCCTATCAC
cDNA|Hyd-2 ------------------------------------------------------------


Gene|Hyd-2 TCCAGGGTGGTTTGATCTCCATTGGCTGCGTCCCCGTCACGCTCTAA
cDNA|Hyd-2 -----ggtggtttgatctccattggctgcgtccccgtcacgctctaa
                ******************************************
```

Figure S6 Sequencing analysis of the *hyd-2* cDNA in wild-type (WT) and putative mutant strains. (A) Multiple sequence alignment of *hyd-2* amplicons from mutH2|44, mutH2|45, mutH2|15, and mutH2|16 against the wild-type sequence; (B) Multiple sequence alignment of *hyd-2* amplicons from mutH2|19, mutH2|49, mutH2|11, and mutH2|8 with the wild-type sequence revealed diverse mutation types—including base substitutions, deletions, and insertions—highlighted in red.

A. Validation of the putative *hyd-2* mutants was performed through sequencing analysis. Multiple sequence alignment of *hyd-2* amplicons from mutH2|44, mutH2|45, mutH2|15, and mutH2|16 against the wild-type sequence revealed base substitution mutations distributed across the gene, highlighted in red.

sghyd2-1

```
            1 ........10........20........30........40........50........60........70........80
GbPER71H2   1 ATGTTCTCTCGCGCCGTCGCCTTCACTACCCTCGCTCTGCCCCTCCTTGCGGTCGCCACTCCCGTCGAGCTGGTGGCTCG
mutH2|44    1 ATGTTCTCTCGCGCCGTCGCCTTCACTACCCTCGCTCTGCCCCTCCTTGCGGTCGCCACTCCCGTCGAGCTGGTGGCTCG
mutH2|45    1 ATGTTCTCTCGCGCCGTCGCCTTCACTACCCTCGCTCTGCCCCTCCTTGCGGTCGCCACTCCCGTCGAGCTGGTGGCTCG
mutH2|15    1 ATGTTCTCTCGCGCCGTCGCCTTCACTACCCTCGCTCTGCCCCTCCTTGCGGTCGCCACTCCCGTCGAGCTGGTGGCTCG
mutH2|16    1 ATGTTCTCTCGCGCCGTCGCCTTCACTACCCTCGCTATGCCCCTCCTTGCGGTCGACACTCCCGTCGAGATGGTGGCTCG
```

sghyd2-2

```
           81 ........90.......100.......110.......120.......130.......140.......150.......160
GbPER71H2  81 CCAGAGCTGCAGCACCGGAGCGGTGCAGTGCTGCAACAGCACCGTCGAGGTAAGGCATCTCGTCGACAACGTTAACGGAA
mutH2|44   81 CCAGAGCTGCAGCACCGGAGCGGTGCAGTGCTGCAACAGCACCGTCGAGGTAAGGCATCTCGTCGACAACGTTAACGGAA
mutH2|45   81 CCAGAGCTGCAGCACCGGAGCGGTGCAGTGCTGCAACAGCACCGTCGAGGTAAGGCATCTCGTCGACAACGTTAACGGAA
mutH2|15   81 CCAGAGCTGCAGCACCGGAGCGGTGCAGTGCTGCAACAGCACCGTCGAGGTAAGGCATCTCGTCGACAACGTTAACGGAA
mutH2|16   81 CCAGAGATGCAGCACCGGAGCGGTGCAGAGCTGCAACAGCACCGTCGAGGTAAGGCATCTCGTCGACAACGTTAACGGAA
```

```
          161 .......170.......180.......190.......200.......210.......220.......230.......240
GbPER71H2 161 CCATCTGCTCACGCGCCTCTTACCCTCCCACTCGCAGGCCAACTCGGCGTCGGCCAACTTGCTCCTTGGTCTGCTTGGCA
mutH2|44  161 CCATCTGCTCACGCGCCACTTACCCTCCCACTCGCAGGCCAACTCGGCGTCGGCCAACTTGCTCCTTGGTCTGCTTGGCA
mutH2|45  161 CCATCTGCTCACGCGCCTCTTACCCTCCCACTCGCAGGACAACTCGGCGTCGGCCAACTTGCTCCTTGGTCTGCTTGGCA
mutH2|15  161 CCATCTGCTCACGCGCCTCTTACCCTCCCACTCGCAGGCCAACTCGGCGTCGGCCAACTTGCTCCTTGGTCTGCTTGGCA
mutH2|16  161 CCATCTGCTCACGCGCCTCTTACCCTCCCACTCGCAGGCCAACTCGGCGTCGGCCAACTTGCTCCTTGGTCTGCTTGGCA
```

sghyd2-3

```
          241 .......250.......260.......270.......280.......290.......300.......310.......320
GbPER71H2 241 TTGTCCTCGGGGACATCACTGGCCTCATTGGCCTGAACTGCTCGCCCCTCACCGTCATCGGTGTTGGCTCCGGCAACGCC
mutH2|44  241 TTGTCCTCGGGGACATCACTGGCCTCATTGGCCTGAACTGCTCGCCCCTCACCGTCATCGGTGTTGGCTCCGGCAACGCC
mutH2|45  241 TTGTCCTCGGGGACATCACTGGCCTCATTGGCCTGAACTGCTCGCCCCTCACCGTCATCGGTGTTGGCTCCGGCAACGCC
mutH2|15  241 TTGTCCCCGGGGACATCACTGGCCTCATTGGCCTGAACTGCTCGCCCCTCACCGTCATCGGTGTTGGCTCCGGCAACGCC
mutH2|16  241 TTGTCCTCGGGGACATCACTGGCCTCATTGGCCTGAACTGCTCGCCCCTCACCGTCATCGGTGTTGGCTCCGGCAACGCC
```

```
          321 .......330.......340
GbPER71H2 321 TGCTCCGCCCAGGCTGTCTG
mutH2|44  321 TGCTCCGCCCAGGCTGTCTG
mutH2|45  321 TGCTCCGCACAGGCTGTCTG
mutH2|15  321 TGCTCCGCCCAGGCTGTCTG
mutH2|16  321 TGCTCCGCCCAGGCTGTCTG
```

B. **Validation of the putative *hyd-2* mutants was conducted using sequencing analysis. Multiple sequence alignment of *hyd-2* amplicons from mutH2|19, mutH2|49, mutH2|11, and mutH2|8 with the wild-type sequence revealed diverse mutation types—including base substitutions, deletions, and insertions—highlighted in red.**

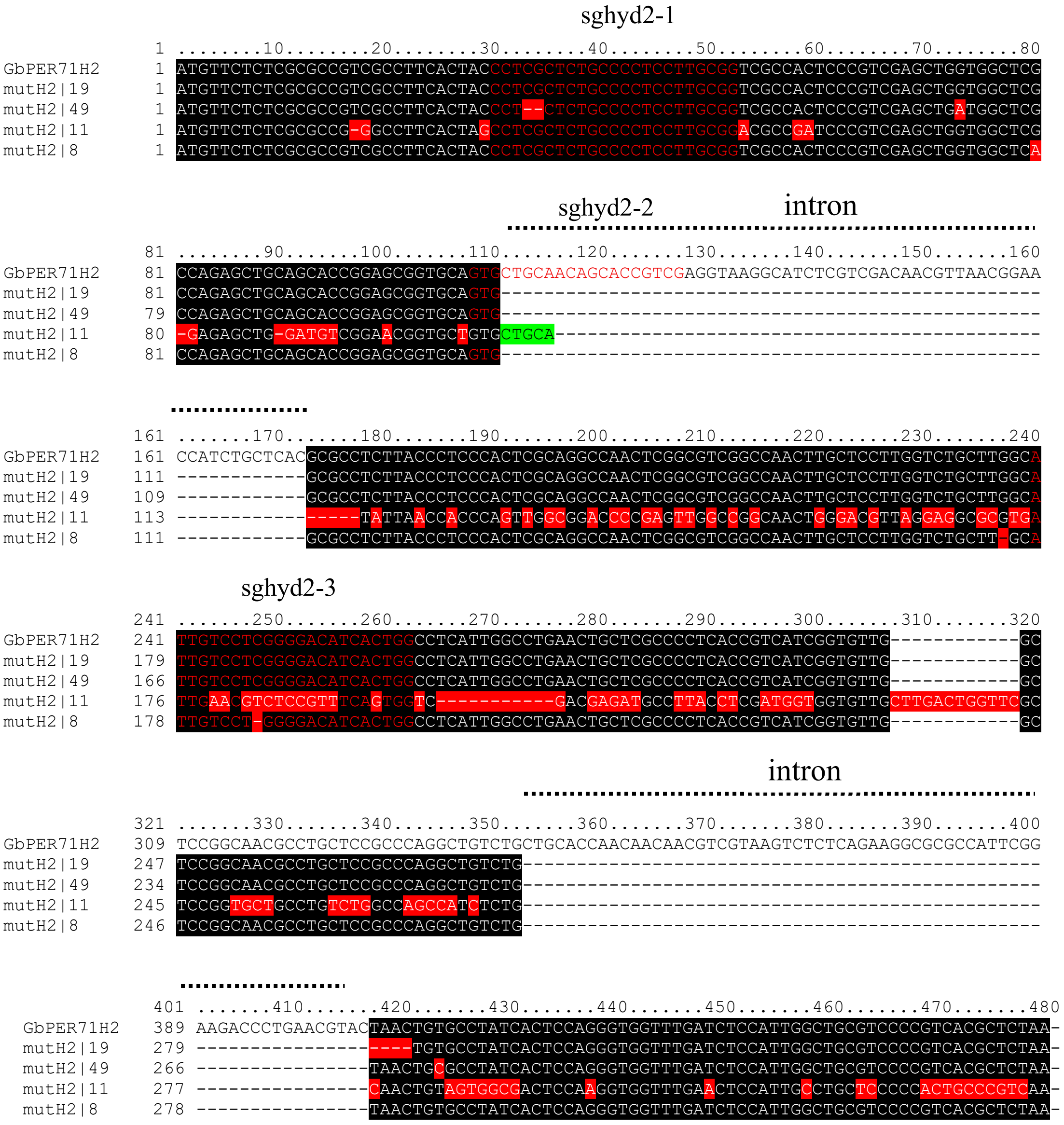

Figure S7 Detailed analysis of the hydrophobin-2 protein. (A) Kyte–Doolittle hydropathy plot showing the hydropathy profile of the predicted Hyd-2 protein. (B) InterPro domain annotation illustrating conserved functional domains and motifs.

A. Kyte–Doolittle hydropathy plot of the hydrophobin-2 protein showing alternating hydrophobic and hydrophilic regions. A major hydrophobic peak at positions 35–40 suggests a potential self-assembly domain typical of hydrophobins.

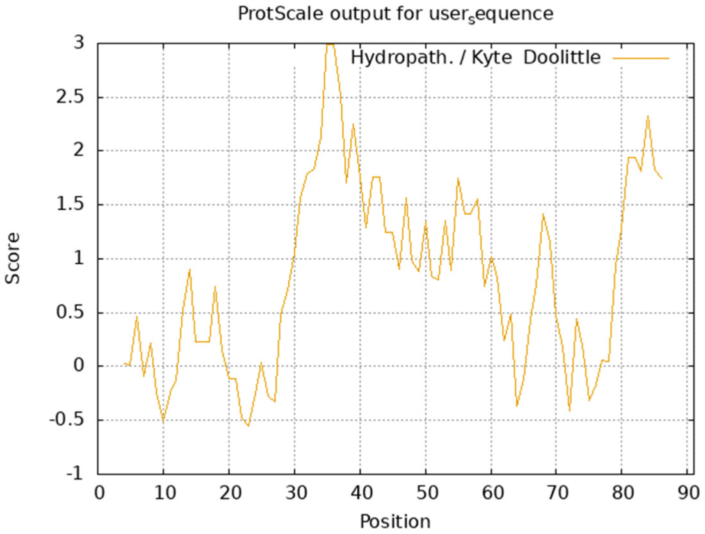

B. InterPro annotation of the hydrophobin protein. Domain architecture and hydropathy profile of the hydrophobin protein

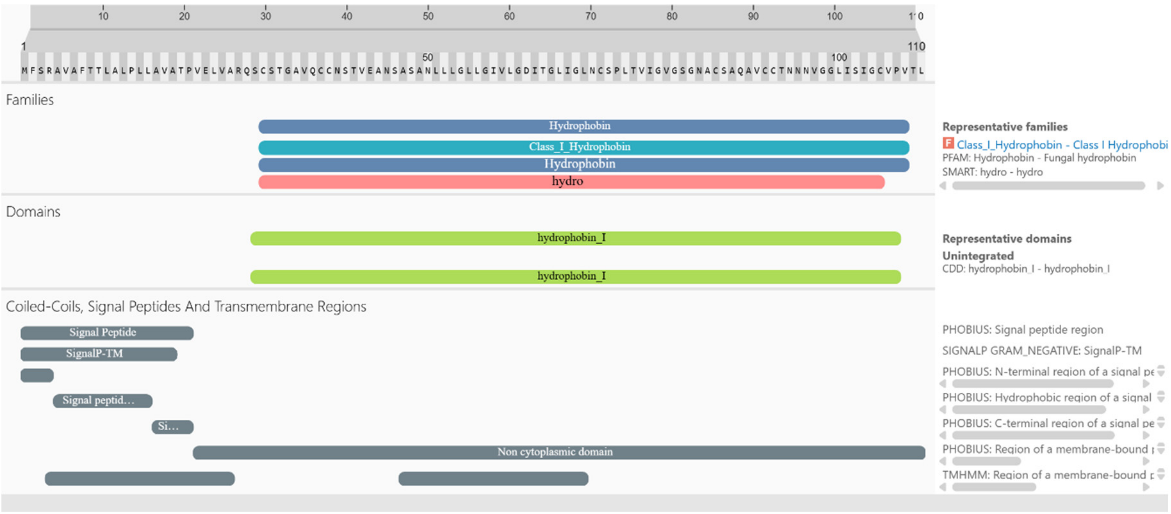

Table S1 The primer sequences used in this project

No	Gene name	Abbr	Primer sequence	Annealing Temperature (Tm), °C
1	*ß-tubulin*	*ß-tub*	Forward: 5′-GAGTGGATTCCCGGACAATGTTTCG-3′<br>Reverse: 5′-CTTGTACATAGCGGAGAACTGAGCGA-3′	57
2	*40S subunit*	*40S*	Forward: 5′-CTCGAAACGCCTCCGGAACAA-3′<br>Reverse: 5′-GGACGTAGTTGTCCTTGCGCT-3′	57
3	*α-tubulin-1*	*α-tub1*	Forward: 5′-AGACCTCTTCCGGCAAGCAC-3′<br>Reverse: 5′-ATCTTCCTTGCCCGTCACAAGAGT-3′	57
4	*hydrophobin-2* (For qPCR)	*hyd-2*	Forward: 5′-CCAACTTGCTCCTTGGTCTGCTT-3′<br>Reverse: 5′-AATGGAGATCAAACCACCGACGTTGTT-3′	57
5	*hydrophobin-2* (For full length gene amplification)	*hyd-2*	Forward: 5′-TCACTGCTCCGAACAGGACAAC-3′<br>Reverse: 5′-GAAACTCGAAGTACGAAGAACGGTGG-3′	57
6	*orotidine-5′-monophosphate (OMP) decarboxylase*	*pyr*G	Forward: 5′-ATGGCATCGGTGGCGAA-3′<br>Reverse: 5′-TTAGTCCATGGATACCAACTCTCTCCT-3′	57

Table S2 Statistical analysis of DSI Scores from Weeks 3 to 7 with Two-Way Repeated Measures ANOVA Summary

Disease Severity Index (DSI) scores for each sample, recorded weekly from week 3 to week 7.

	Control				
Ramet	Week 3	Week 4	Week 5	Week 6	Week 7
1	5	10	10	10	15
2	20	40	80	96	100
3	15	56	90	100	100
4	12	36	68	88	96
5	16	36	48	60	92
6	3	13	16	16	73

	Δhyd2-11				
Ramet	Week 3	Week 4	Week 5	Week 6	Week 7
1	5	5	5	5	5
2	0	0	0	0	0
3	4	8	48	80	100
4	0	0	0	0	0
5	0	10	10	10	10
6	0	16	16	16	16

	Δhyd2-19				
Ramet	Week 3	Week 4	Week 5	Week 6	Week 7
1	0	8	12	12	12
2	0	0	0	0	0
3	0	5	10	10	10
4	0	8	16	20	20
5	0	0	0	0	0
6	0	0	0	0	0

Statistical analysis of DSI scores using a two-way repeated measures ANOVA:

Two-way RM ANOVA	Matching: Both factors				
**Alpha**	0.05				
**Source of Variation**	**% of total variation**	**P value**	**P value summary**	**Significant?**	
**Time**	13.47	<0.0001	****	Yes	
**Genotype**	38.04	0.0019	**	Yes	
**Interaction: Time x Genotype**	8.638	<0.0001	****	Yes	
**Interaction: Time x Subjects**	5.079				
**Interaction: Genotype x Subjects**	15.15				
**Subjects**	12.17				
**ANOVA table**	SS	DF	MS	F (DFn, DFd)	P value
**Time**	11333	4	2833	F (4, 20) = 13.26	P<0.0001
**Genotype**	32004	2	16002	F (2, 10) = 12.55	P=0.0019
**Interaction: Time x Genotype**	7267	8	908.4	F (8, 40) = 5.801	P<0.0001
**Interaction: Time x Subjects**	4273	20	213.6		
**Interaction: Genotype x Subjects**	12747	10	1275		
**Subjects**	10240	5	2048		
**Residual**	6264	40	156.6		

Multiple comparison

Within each row, compare columns (simple effects within rows)

**Number of families**	5
**Number of comparisons per family**	3
**Alpha**	0.05

Tukey's multiple comparisons test	Mean Diff.	95.00% CI of diff.	Significant?	Summary	Adjusted P Value
**Week 3**					
**Control vs. ∆hyd2-11**	10.33	-7.251 to 27.92	No	ns	0.335
**Control vs. ∆hyd2-19**	11.83	-5.751 to 29.42	No	ns	0.242
**∆hyd2-11 vs. ∆hyd2-19**	1.5	-16.08 to 19.08	No	ns	0.977
**Week 4**					
**Control vs. ∆hyd2-11**	25.33	7.749 to 42.92	Yes	**	0.003
**Control vs. ∆hyd2-19**	28.33	10.75 to 45.92	Yes	***	0.001
**∆hyd2-11 vs. ∆hyd2-19**	3	-14.58 to 20.58	No	ns	0.91
**Week 5**					
**Control vs. ∆hyd2-11**	38.83	21.25 to 56.42	Yes	****	<0.0001
**Control vs. ∆hyd2-19**	45.67	28.08 to 63.25	Yes	****	<0.0001
**∆hyd2-11 vs. ∆hyd2-19**	6.833	-10.75 to 24.42	No	ns	0.615
**Week 6**					
**Control vs. ∆hyd2-11**	49.83	32.25 to 67.42	Yes	****	<0.0001
**Control vs. ∆hyd2-19**	61.33	43.75 to 78.92	Yes	****	<0.0001
**∆hyd2-11 vs. ∆hyd2-19**	11.5	-6.085 to 29.08	No	ns	0.261
**Week 7**					
**Control vs. ∆hyd2-11**	54.17	36.58 to 71.75	Yes	****	<0.0001
**Control vs. ∆hyd2-19**	69	51.42 to 86.58	Yes	****	<0.0001
**∆hyd2-11 vs. ∆hyd2-19**	14.83	-2.751 to 32.42	No	ns	0.113

Test details	Mean 1	Mean 2	Mean Diff.	SE of diff.	N1	N2	q	DF
**Week 3**								
**Control vs. ∆hyd2-11**	11.83	1.5	10.33	7.225	6	6	2.023	40
**Control vs. ∆hyd2-19**	11.83	0	11.83	7.225	6	6	2.316	40
**∆hyd2-11 vs. ∆hyd2-19**	1.5	0	1.5	7.225	6	6	0.2936	40
**Week 4**								
**Control vs. ∆hyd2-11**	31.83	6.5	25.33	7.225	6	6	4.959	40

**Control vs. Δhyd2-19**	31.83	3.5	28.33	7.225	6	6	5.546	40
**Δhyd2-11 vs. Δhyd2-19**	6.5	3.5	3	7.225	6	6	0.5872	40
**Week 5**								
**Control vs. Δhyd2-11**	52	13.17	38.83	7.225	6	6	7.601	40
**Control vs. Δhyd2-19**	52	6.333	45.67	7.225	6	6	8.939	40
**Δhyd2-11 vs. Δhyd2-19**	13.17	6.333	6.833	7.225	6	6	1.338	40
**Week 6**								
**Control vs. Δhyd2-11**	68.33	18.5	49.83	7.225	6	6	9.755	40
**Control vs. Δhyd2-19**	68.33	7	61.33	7.225	6	6	12.01	40
**Δhyd2-11 vs. Δhyd2-19**	18.5	7	11.5	7.225	6	6	2.251	40
**Week 7**								
**Control vs. Δhyd2-11**	76	21.83	54.17	7.225	6	6	10.6	40
**Control vs. Δhyd2-19**	76	7	69	7.225	6	6	13.51	40
**Δhyd2-11 vs. Δhyd2-19**	21.83	7	14.83	7.225	6	6	2.904	40

Table S3 RT-qPCR expression profiles of individual hydrophobin genes

ΔC_q values for each hydrophobin gene after normalization against three housekeeping genes: ß-tubulin, α-tubulin-1, and the 40S ribosomal subunit:

	3dpi	5dpi	7dpi	9dpi	21dpi	28dpi	35dpi	42dpi	49dpi	56dpi
**Hyd1**	-6.06	-5.36	-6.27	-6.55	-7.29	-3.12	-2.96	-4.84	-7.70	-0.02
**Hyd2**	-3.16	-3.48	-4.56	-5.48	-6.21	-2.30	-2.13	-3.94	-6.60	1.01
**Hyd3**	-4.06	-2.17	-3.43	-2.38	-3.79	-0.88	-1.51	-2.29	-4.44	-0.09
**Hyd4**	0.13	-0.44	-1.27	-2.29	-3.50	1.86	3.13	-0.66	-2.99	5.06
**Hyd5**	2.04	2.58	0.81	1.07	0.77	1.78	1.15	0.70	0.11	1.10
**Hyd6**	0.47	0.27	-1.99	1.01	-0.08	0.82	1.31	0.27	1.34	0.58
**Hyd7**	-2.96	0.24	-3.01	-0.90	2.43	-0.19	1.35	0.81	2.62	0.72
**Hyd8**	0.85	-2.51	-3.79	-3.60	-5.02	-1.54	-2.43	-2.43	-5.02	-0.54
**Hyd9**	-0.54	0.50	1.17	1.36	3.54	0.75	0.34	1.06	2.74	-0.01

The value of standard error (SE) from four biological replicates.:

	3dpi	5dpi	7dpi	9dpi	21dpi	28dpi	35dpi	42dpi	49dpi	56dpi
**Hyd1**	1.80	1.62	1.37	0.95	1.75	0.85	0.85	1.88	2.15	0.86
**Hyd2**	0.50	1.43	1.10	1.14	0.92	1.02	1.33	1.14	0.82	0.61
**Hyd3**	0.63	0.60	0.66	0.58	0.92	0.44	0.54	0.65	0.88	0.40
**Hyd4**	0.63	1.42	1.20	1.62	0.92	1.43	1.85	1.48	0.81	1.76
**Hyd5**	0.63	0.60	0.66	0.58	0.92	0.44	0.54	0.65	0.88	0.40
**Hyd6**	0.74	0.71	0.57	0.47	0.92	0.18	0.62	0.49	1.14	0.22
**Hyd7**	1.41	0.69	0.74	0.81	0.92	0.14	0.62	0.40	0.82	0.38
**Hyd8**	0.47	1.10	0.72	0.95	0.92	0.39	1.00	0.92	0.81	0.20
**Hyd9**	0.97	0.70	0.81	0.62	0.92	0.23	0.49	0.46	0.81	0.21

Table S4 The hydrophobin-2 protein sequences used in the construction of phylogenetic tree analysis

<table>
<tr><th>No</th><th>Protein Accession Number and sequence</th><th>Origin</th><th>Class of Hydrophobin</th></tr>
<tr><td>1</td><td>>A0A0J9UJP6|Fo.hydrophobin-1
MQFYTIVSLFLAGTAYALPATSANGYEACPSGGLFGNPQCCSLNLVGVLSGD
CRAPTKTPNSAKEFQAICAESGQKARCCGLSEILELGAFCQKPVGVSA</td><td>Fusarium oxysporum</td><td>Class II</td></tr>
<tr><td>2</td><td>>A0A6V8R0V1|Ta.hydrophobin-1
MYASVIIYTLVALCGVMSSPVQEAEVAPRAIEPRMPTFADIPFPRFPSHNNH
HHNEKDKGKDKGNGKGVDKNNGGGCDTGTNTQLNACSAGSPYCCSSDGNGGH
ICSNTTACDQKVICCNNNNGFQICIGEIDFNVPVTINIIYD</td><td rowspan="4">Trichoderma asperellum</td><td>Class I</td></tr>
<tr><td>3</td><td>>I7AQG9|Ta.hydrophobin-2
MQFFTVATLFLATAFAAPSVDTNGNGIAPRLAANFCPPGLLYSNPQCCQIDV
LGVADLDCVSPPSGPSKCKTFAGICDKIGRQPKCCVVPVAGQALLCTDPVGA
NN</td><td>Class II</td></tr>
<tr><td>4</td><td>>A0A2T3ZJ73|Ta.hydrophobin-4
MPASNSCSPPGGDHKPPPPPPSHPSPSPTPSYPPPPNNGGGYYPPPPDNTSH
TPPPPSSTPPNNGGGGNDGGSGNTGGGNSDDGSDGGHSICPSGLYSSAQCCS
TDVLGIADLNCKPPSRTPKDGSDFAKICSNTGSEARCCVIPVAGQALLCEDV
VGAN</td><td>Class II</td></tr>
<tr><td>5</td><td>>A0A2T3Z3L3|Ta.hydrophobin-5
MKFFAAAALFIAGVLAAPSPNAANSVTPLCNPGLYSNAQCCAVDVLGVADLN
CATPPGVVNNAAEFQAACAKIGQEARCCVLPVLGQDVLCETPLGL</td><td>Class II</td></tr>
<tr><td>6</td><td>>2610494|Sc.hydrophobin-1
MRFSLALLALPALAAAAAVPGGGKGAGQACNSGPVQCCNETTTVANAQKQGL
LGGLLGVVVGPITGLVGLNCSPISVVGVLTGNSCTAQTVCCDHVTQNGLVNV
GCTPISL*</td><td rowspan="7">Schizophyllum commune</td><td>Class I</td></tr>
<tr><td>7</td><td>>2628225|Sc.hydrophobin-2
MKLVTFFAFLFALLIAVSAVPTAETNADRMRRGLTPMRPRNLGRATPVDSAS
KHKPSGGGGNGQSCSSGPVQCCQSVQKSNSGSLLGIILKLLGIVLGEVTDIG
LHCTPIDVLGGGGNQCSAQTVCCKDNSYSGHGINIGCSPINIGL*</td><td>Class I</td></tr>
<tr><td>8</td><td>>2629420|Sc.hydrophobin-3
MRFFATLVLALPALAMATAVPRDVNGGTPPKSCSSGPVYCCNKTEDSKHLDK
GTTALLGLLNIKVGDLKNLVGLNCSPISVIGVGGNSCSAQTVCCTNTYQHGL
VNVGCTPINIGL*</td><td>Class I</td></tr>
<tr><td>9</td><td>>2369054|Sc.hydrophobin-4
MRVVALSALFASSLLGVGALPSASGCSAEGIWCCNENAIRTDISGLIGKGCT
QTSIIGIGGTRCQGQTVCCDKTDQNGILNAGCKPTILGV*</td><td>Class I</td></tr>
<tr><td>10</td><td>>2547619|Sc.hydrophobin-5
MRFSILVFALPALVAATGTPASCSGGGVYCCNKTVDHNDKSIQKQLSLVGVT
AGSIHDLVGLTCTPITAIGVGSGAQCSAQSVCCSKSYQNGLVNVNCTPIKIL
*</td><td>Class I</td></tr>
<tr><td>11</td><td>>2485769|Sc.hydrophobin-6
MRFTIALFAALPLLVAASAVPRAPGGQKCRAESVQCCNTTYSHKDYSHVEYL
QGLGISPEVLANPDASSYADCTPLSVVGGHGCQASSTAVCCENNYQRGAVNM
GCVPISMAA*</td><td>Class I</td></tr>
<tr><td>12</td><td>>2526471|Sc.hydrophobin-7
MFARIFTIVSVAMLVAAQPHGRGLPVLGGILGGGGGGGGATVTVTVPGECP
TVTQTVTVTGSGTWGVPTSSSTWGVPTSSSTWGVPSSSSSSVHSSSTSKSDE
HTSSSSVPTWGPTGTPTWTSKTEDPTSKTSTTSHSSVNTSTWGTSKTDTTSA
TWGTGSSSVPSSSSTSKDTGSTTWTSEPTKPTDPGNGGGQCNSGTVKCCNSV
KKVKDVGSAGLIAGLLAIGGLNLDSLVGVQCSPLSVIGVGGNSCSSQVACCS
NNTYNGLINIGCVPISISL*</td><td>Class I</td></tr>
</table>

13	>BAB84547.1|Pn.hydrophobin-1<br>MQFKVLAALVIGATLAAATGSPSNQCNTGSLQCCNSTGSATDPAIAKLFALL<br>GINVEDVTALVGVTCSPITVVGASGSSCSEQPVCCTNDSFNGIVALGCAPIN<br>LNL	*Pholiota nameko*	Class I
14	>XP_001878874.1|Lb.hydrophobin-1<br>MKFTIIFTILSALFFAAVSAAPSLTNAQRLARGWSPNPPARRTTPVAAAKRG<br>QPSGIHNSCNTGTMHCCNSVTSSNSRVASTLAGLLGIVLPHNTAVGITCSPL<br>SAVGLGHGGTCTQQPVCCTGNNFSGLVVVGCSPININVT	*Laccaria bicolor* S238N-H82	Class I
15	>XP_001882931.1|Lb.hydrophobin-2<br>MKFSRLSTALVLLASGAAVSATQTVTYAPVQTPPPASQCNTGALWCCNNTQA<br>ANGSIIAPLLALLGIVVDPITALLGSGCTSINFLGVGTGGMCTAQPVCCQNN<br>NFSGLVVIGCTPVTLGV		Class I
16	>ABG33849.1|Pi.hydrophobin-1<br>MFTRVFTVVSVAALAIAGAAPTSQCNTGTTQCCNNVQSASSMNQLLSAVGFV<br>NVLAGLTGNVGTDCTPITAVGTGSGADCTAQPVCCTDNTFNGLINIGCTPIN<br>INA	*Paxillus involutus*	Class I
17	>ABG66317.1|Pi.hydrophobin-2<br>MKFTYVFALAAAATVVSAETNAQRMARGLPPKGPARRGTPSFGAKRGSPSGT<br>PSGGSQCNTGPVQCCNSVQQCGSGNEVDTMLSLLGLSVPAGTNVGTNCSPIS<br>AVGTGSGASCTXQPVCCENNNFNGLVNIGCTPININL		Class I
18	>AAX51848.1|Pi.hydrophobin-3<br>MKFTYVFALAAAATVVSAETNAQRMARGLPPKGPARRGTPSFGAKRGSPSGT<br>PSGGSQCNTGPVQCCNSVQQCGSGNEVDTMLSLLGLSVPAGTNVGTNCSPIS<br>AVGTGSGASCTQQPVCCENNNFNGLVNIGCTPININ		Class I
19	>P41746|Af.hydrophobin-1<br>MKFSLSAAVLAFAVSVAALPQHDVNAAGNGVGNKGNANVRFPVPDDITVKQA<br>TEKCGDQAQLSCCNKATYAGDVTDIDEGILAGTLKNLIGGGSGTEGLGLFNQ<br>CSKLDLQIPVIGIPIQALVNQKCKQNIACCQNSPSDASGSLIGLGLPCIALG<br>SIL	*Aspergillus fumigatus*	Class I
20	>Q4WBR8|Af.hydrophobin-2<br>MLVTMRLSRSIAVFTLVTYATGLPSLQVIPRGEPQLPLLDPSMTIKEAARKC<br>GDKAQLSCCNRVVKAGDYTSVDEGIGAGLLSNLAGGGSGISSILAFDQCSRL<br>DAQVPVVLLPIQDLLNQHCKQNVACCQKNPGDASSSGVGVSLPCIALGSVA		Class I
21	>Q4WC31|Af.hydrophobin-3<br>MHPRPEHLSPTLNHNHRLKLKQPATTSRMLARTILATLVSAACFATVNAQTC<br>SATNAVCCQQLQDPDNLNADALNLLRLLNINPNTLTGAVGLTCKRLTEACFA<br>CLLLTRPGTSLVSGSCNANAACCTGNNYNGLIVLGCTQIQV		Class I
22	>E9QT94|Af.hydrophobin-4<br>MKFLAVVSLLAATALALPNAGVVHPTFASADKYTLQQAQNKCGEHTTLSCCN<br>HVSKVGDTTAFNYGLLNGLLGNAISGPEGVGILSGCQKISVTALIGVDDLLN<br>KQCQQNVACCQDNKSVATGGLINIATPACVALDSII		Class I
23	>Q4WEK0|Af.hydrophobin-5<br>MRPITILCTLATLSTTLAVPFSQASKSTSASRSTSSSTVPASLPSPTLSGPN<br>ACPPNKFKQCCTTLSQVGDDLLKPLGAVVPLVGAIQVNSLVGVSCRPMADAA<br>PESTCGNAVMCCDSSTVGGDDLMQTSCQDFALAKKREREAIERQQRRFSEYQ<br>RMMLSQSATPAPTSTGVDVMGSSFSKAKATPTRV		Class III
24	>ABA46362.1|Ha.hydrophobin-1<br>MFARVSTLFAMFFLGLALMVSATPAALKPVARDTIPASQCNTGDLQCCNTVE<br>NADSPSAAALLGLLGVVVQGLDVLVGLTCTPITVIGVGSGANCVQQPVCCEN<br>NNFNGLINIGCTPVNLGL	*Heterobasidion annosum*	Class I

<table>
<tr><td>25</td><td>>ABA46363.1|Ha.hydrophobin-2
MFFRISTVFVVALAAFAAASPAPWGAPPPTTTTHPPVTTTVTVTAPATTTTI
PASQCNTGDAQCCNSVQSATAPAVTSLLGLLGIVLEDINVLVGLSCDPISVI
GVGGGANCVQQPVCCENNNFNGLINIGCTPINIFL</td><td></td><td>Class I</td></tr>
<tr><td>26</td><td>>EIW80221.1|Cp.hydrophobin-1
MFTRVASIIALATFALAAPAATTNQCNTGSAQCCNTVDSASSPQVATVLGLL
GIVVGDITGQVGLQCSPLSVLSSGGGTCNQEPVCCTNNSFNGLVNLGCSPIN
VGL</td><td rowspan="5">Coniophora puteana
RWD-64-598 SS2</td><td>Class I</td></tr>
<tr><td>27</td><td>>EIW79624.1|Cp.hydrophobin-2
MKFTTAVAIAAAATVVAAETNAHRMARGLPPKAPIRRSKTAAPKAQASGGSG
GDCNTGPVQCCNSVHKADESIVQKLLDLLGLSGIPGDTQVGMNCSPVSVIGS
GGGQCNQEPVCCENNNFSGLINLGCSPININL</td><td>Class I</td></tr>
<tr><td>28</td><td>>EIW85899.1|Cp.hydrophobin-3
MRAQLSSVVVLSAGVLAAAMPWETTSPATSTTTVTVTAPASTSTEPASSCDT
GSLQCCDTVTSAKSESASAILGLLGVVLSNLDVLVGLTCSPISVLSSGSSGC
TADPVCCENNNFSGLISLGCVPVNLSLK</td><td>Class I</td></tr>
<tr><td>29</td><td>>EIW86203.1|Cp.hydrophobin-4
MRASTFAALTLAAVVAADQCNTGSAQCCNSVKQADDESLTGIFGLLGIVVGE
VTGQVGVDCSPIGALSSGGGQCNQHPVCCTNNTFKGLVNLGCSPINVGL</td><td>Class I</td></tr>
<tr><td>30</td><td>>EIW85975.1|Cp.hydrophobin-5
MLSRLSAAVVLSLSLLAVAKPIADPYRGLSNAQRLSRGLPIAMPRNLRKLSN
RGPSKAYEKRHKPSSTPTSTGWCTTGSAQCCESVESASNPVAKTLLGLLGIV
LSDLDVLVGITCSPINILGLGLGECWEKPVCCENNDFGGLISIGCSPISIEL</td><td>Class I</td></tr>
<tr><td>31</td><td>>Q75T84|Fv.hydrophobin-1
MRLSISKLSVVMLALPILASASVLPRTGSTGASCGSTTSGQAQCCNSTQSNT
TPLPFPLSVITGLLGIDLGAITGLVGLTCNPITVLGLGGTNCNSQAVCCTGN
TFNGLINLGCSPLNVAL</td><td>Flammulina velutipes</td><td>Class I</td></tr>
<tr><td>32</td><td>>CAA74987.1|Po.hydrophobin-1
MFFRTSSLFTTIVAFTVMAAAMPGNPKPTTTTVTVTAPAHPTATAPASECKT
GPVQCCNSVQSSKSPAASLLLGLLGIVLQGVAVPVGLTCNPITVIGVGGNSC
SAQTVCCENNNFSGLIAIGCTPINLSL</td><td rowspan="6">Pleurotus ostreatus</td><td>Class I</td></tr>
<tr><td>33</td><td>>CAA76494.1|Po.hydrophobin-2
MFSRVIFCTFLILPLLAAATAIPRTNPPAPTCTTGSLQCCNSVQAASNPVVG
LLAGLLGIVLGPITGQVGLTCSPITVIGVGGTSCSAQTVCCNGNSFNGLIVV
GCSPVNISL</td><td>Class I</td></tr>
<tr><td>34</td><td>>Q9Y7H0|Po.hydrophobin-3
MFFQTTIVAALAFLAVATPLALRTDSRCNTESVKCCNKSEDAETFKKSASAA
LIPIKIGDITGKVYSECSPIVGLIGGSSCSAQTVCCDNAKFNGLVNIGCTPI
NVAL</td><td>Class I</td></tr>
<tr><td>35</td><td>>A0A067N4H9|Po.hydrophobin-4
MFSRVMFCTFLILPLLAAATAIPRTDTPSCSTGSLQCCSSVQKASDPLVGII
VALLGIVLGPLDLNVGLTCSPITVIGVGGTSCTQQTVCCTGNNFDGLIVAGC
SPINIGL</td><td>Class I</td></tr>
<tr><td>36</td><td>>A0A067P1K6|Po.hydrophobin-5
MLFKQAILVATTLTTLAVATPVVDVRRRTDPASSCSTGTLNCCNSSGTVEDK
TIAGLLGILNIVVSDITALVGITCTPITVVGAGGTSCTSQTLCCDNNNFSGL
ITLGCIPININL</td><td>Class I</td></tr>
<tr><td>37</td><td>>A0A067NHE1|Po.hydrophobin-6
MYSQSMVLLAAAFASFVAASPMALHGANSCSTGPVHCCNSVEHHTQPHANSL
ARGSGLVGLDIETLVGNIGLDCSPIGLSSTDCTAQTVCCDDVTFDGKVAVGC
TPVNLSVL</td><td>Class I</td></tr>
</table>

38	>A0A067P943|Po.hydrophobin-7 MKFTSVIALVATAATLVGAVPFETNAERLARGLPPLPPARRASGVEAAKPKPSPSHGCSTGPVQCCNDLVDRSNHTVGILIGLLGIVLGPVTGLFGLGCSPLLGGGAKCQSQTVCCSDNKFSGIINIGCSPINIGL		Class I
39	>O60047|Po.hydrophobin-8 MFSIRIATVVLAASAALRPPARITNTETPVNQCGSGSIQCCESVQSASAAQAAGILGPLDILTNLQGLVGSHCSPLAAVGVSGTSCSSQTVCCKDVSKSGLVNLGCSPINLNL		Class I
40	>CAD12833.1|Po.hydrophobin-9 MFSRVIFCTFLILPLLAAATAIPRTDTPSCSTGSLQCCSSVQKATDPLASLLIGLLGIVLGPLDLLVGVTCSPITVIGVGGTSCTQQTVCCTGNSFNGLIAIGCSPINISL		Class I
41	>CAD12834.1|P0.hydrophobin-10 MFSRVIFCTFLILPLLAAATAIPRTDTPSCSTGSLQCCSSVQKASDPLVGIIVALLGIVLGPLDLNVGLTCSPITVIGVGGTSCTQQTVCCTGNNFNGLIVAGCSPINIGL		Class I
42	>ACM89293.1|Pg.hydrophobin-1 MKLTTASILALPLLAVATPLEVRQNGSCDTGSLQCCTETISGTSAQGNVLLGLLGIVLSDVTGLLGLGCSPISVVGVGSGNACTASPVCCSNNSVGGLINIGCIPITL	*Phlebiopsis gigantea*	Class I
43	>EIN08599.1|Ps.hydrophobin-1 MFFSRSTVFVALSALVATAAAAPSTLPASQCNTGSIQCCNSTQKASSAGMSDLLGLLGVVVSDVTALVGSNCSPISAVGLGGNSCSAAPVCCENNQFNGLIALGCVPVNLSL	*Punctularia strigosozonata* HHB-11173 SS5	Class I
44	>EIN05963.1|Ps.hydrophobin-2 MFARSTLFVAVAALFAVASADQCNTGSIQCCNSVHDSKSSALSGLFNLLGINAGDVTGQVGVTCSPISAVGVGGDSCNASPVCCENNNFNGLVALGCVPISLSL		Class I
45	>AF176648_1|Le.hydrophobin 1 MQFKLAFVSIALATLAVATPAPRGEPASSCSTGDLQCCNTVEPASSPSASTILGLLGIVIQGVDVLVGLTCSPITVIGLESGGCSAQAVCCTDNSNGGLISIGCLPVTL	*Lentinula edodes*	Class I
46	>92693|Gl.hydrophobin-1 MFARVAAFTLLALPLFAVATPTVLRRDSCNTGDIQCCNSVQNSNETAISTLLSLLGVNAQDVTGQVGLQCSPLTVIGAGVGSSCSQAPVCCQNNNVGGLISVGCVPIQL	*Ganoderma lucidum*	Class I
47	>92783|Gl.hydrophopbin-2 MLSRAVVALSAILSLPILATAASIADPLGSSSGSGGVCTTGTLNCCQTTSTSSDPSTNLILSLIGVVVQGVDVAVGLNCDPITAIGVGISNSCQAQPACCPDNATGNAVSIGCVPVSL*		Class I
48	>188736|Gl.hydrophobin-3 MSSARFAFLAFLAFAAIVSASPVPCATTTVTVWAPFPAPTAPSGEPTTIIPPLIPEPTIPGGPSLPITIVPLPSSISTGPIIIPPTTTVDATSVIPTVEPSLPPFPTFPLPPISSVLPTSIPSLPTIPVPTVEPLPTEPLPTGEPLPTEPLPTGEPLPTISLPTGPLPVPTGPIPTISLPTEPLPTIPLPTIPLPTGEPLPTISLPTEPLPVPTGPIPTISLPTEPHPTIPVPTVSIPTIPTISVPTISVPTISVPTISVPTVTLPTVSVPTGGSGTTTSCTGSAPSSTTTSPSGGNGGKCNTGSLQCCNSLARSGTPEGQKDILGALSSLGSSASGGLLSGLLGVLAEGADVGVGLNCSPITLLGLGGQNSCNAHPVCCENNASGGLISIGCIPITL		Class I
49	>226961|Gl.hydrophobin-4 MIATRMIALSAFAILPALGRVTSLAARDTCDGGSGTVLCCSFLASANSPLVAPILASLGIVPGSLNGLVGVTCEPLEGNDSCGGQRACCVNNDFNGVVALDCTTA*		Class I
50	>359156|Gl.hydrophobin-5 MIFTRAFALILTVLPVLAAANPTALDARADCATGDLQCCDTTETAGSPAGAALLGLLGVVVQDANVLIGVTCSPITVIGVGTGSSCSTNTVCCSDNSHGGLVSIGCVPVTL		Class I
51	>359455|Gl.hydrophobin-6 MFSRFVAASALAFAVLAAAADNCTTGSIQCCNHVEDADSVSGSTLLGALETLGINLQDVTGQIGLQCSPLSAVGAGGSSACSASPVCCQNNNVGGLVSIGCVPISL*		Class I

52	>366628|Gl.hydrophobin-7<br>MMFSHVATFVLASLPLLAAATPLEGRGGEPPSSCTTGPIQCCNTVTTASNPA<br>AAAVLGLLGIVLQDLSIPVGLTCSPISVIGVGGSGACSANAVCCQDNSNGGL<br>ISIGCVPVQL		Class I
53	>395058|Gl.hydrophobin-8<br>MFSKLALTAVVSALLAAATAIPTPDGGAGSCNTGALQCCNTVGQSSDSSVTA<br>GLPALLQVVLKGLNIPIGLNCDPITVIGAGISSSCKAQPVCCSDNSSGGLIS<br>LGCVPISL		Class I
54	>411128|Gl.hydrophobin-9<br>MFSRLAVVSTVAFAVLAAAADNCNTGSMQCCNHVEDSDSVSGSALLGVLATL<br>GINLQDVTGQIGLQCSPLSAVGVGGSSACSASPVCCQNNNVGGLVSIGCVPI<br>SL		Class I
55	>415677|Gl.hydrophobin-10<br>MKLSLSLLASLLLSLFASTSASPHWLPPYSAAVHRRHDDPDCDSAHTYTATY<br>TSDIVRTYTIFSVSTATETYTSLRTVSTRRGGPHSTRTTTRTQTWTEVKTQT<br>VTDWAPGATITITKVQTDTDTDTDVSTHTVSAAAPSASVSPPPGGECNTGSL<br>QCCDTVAPANSPSMSGILSAIGVGMDVLNGLGNGLVGTSCSPISALSLGGGV<br>ECNQSPVCCNNNTYQGLVNIGCVPIQL		Class I
56	>448249|Gl.hydrophobin-11<br>MKFSYAIAAVVAAAASVQAVETNGQRMARGLKPLPPRNIHRRASRTNEARGA<br>TPSGTPSGGDYNCNTGSVQCCNQVSKANDTVISAILGLLGIGGVADDVLVGL<br>KCSPLSVVGLGSGNSCSQRPVCCENNSKGGLVSIGCIPITL		Class I
57	>453709|Gl.hydrophobin-12<br>MIARVAAVAAFFALPLLALAGGVGDCNTGDIQCCNNLEDAHSAAGSGLLASL<br>GINVQDVVGQIGLQCNPVTVIGGQITSTCSQRPVCCQNNNVGGLVSVGCIPI<br>QL		Class I
58	>461584|Gl.hydrophobin-13<br>MFARSIAVSALFALSALAAPTASSGSCNTGSIQCCNSVEDSSSAAGAGILGL<br>LGLNLQDVTGLIGLQCSPLSVVGLGGSSACSQQPVCCQNNNVGGLISIGCIP<br>IQL		Class I
59	>462824|Gl.hydrophobin-14<br>MFSRAVAFATLALPLLAVATPVELAARQSCSTGSEQCCNSVVDSNSASGNLL<br>LGLLGIVLGDITGLIGLNCSPLSVVGVGSGNACSSNAVCCTNNNVGGLISIG<br>CLPITL*		Class I
60	>484177|Gl.hydrophobin-15<br>MFSRAIAYFYFLFALTILAAAMPGGQPPSTTTRTITVTQTATGPEPTSSCTT<br>GPIQCCQSIQSPTDHDASVILGLLDIVIEGLDVLVGLTCSPINVIGVGSGSS<br>CTANVVCCQNNNVGGLISIGCIPIIL*		Class I
61	>498941|Gl.hydrophobin-16<br>MIARVAAVAAFFAFPLLALAGGADSCNTGDIQCCNSFEDSNSAAGSALLATL<br>GINVQDVTGQIGFQCNPVTVIGGQITSTCSQRPVCCQNNNVGGAVSVGCVPV<br>QL*		Class I
62	>500590|Gl.hydrophobin-17<br>MFSRALTALILALLAAAAPQTSQPSCSTGTLQCCQDFQSADSPLLAYTLELA<br>GVALPDTTSLFGLGCTSSDPSAGYSGGACGAGAVCCTNSIADTAIFFGCEPV<br>VPQ*		Class I
63	>512143|Gl.hydrophobin-18<br>MFSRVAILALTALPVLAAATPLEVRGGEPASSCSTGPVQCCNTVTTANSASA<br>SAILGLLGIVLSDLNVPVGLDCSPINVIGVGGSDACSSNAVCCQDNSNGGLI<br>SIGCLPVSL*		Class I
64	>512703|Gl.hydrophobin-19<br>MFARLAAVSTLAFAVAVAAASDVCNTGALKCCNSLEDPKSAVGSVILGALGL<br>DVQDVVAQIGVGCTPITVVGAGGAESCSAKPACCTDNSQGGAVSLGCVPVSL<br>*		Class I
65	>527106|Gl.hydrophobin-20<br>MFARVVTSIYFILSLTILVAAMPGYTPPPTTKTVTVTASPSPPKTTTITVTA<br>PASTPSGGSSCSTGPVQCCNSVGTANDAGFGAILGLLGIVLEGVEGLLGLGC<br>SPISVVGVGSGNACSSNVVCCQNNNIGGLISIGCIAIIL*		Class I

<table>
<tr><td>66</td><td>>555087|Gl.hydrophobin-21
MIARVAAVAAFFAFPLLALAGGADSCNTGDIQCCNSFEDSNSAAGSALLATL
GINVQDVTGQIGFQCNPVTVIGGQITSTCSQQPVCCQNNNVGGAVSVGCVPV
QL*</td><td></td><td>Class I</td></tr>
<tr><td>67</td><td>>EJD00400.1|Fm.hydrophobin-1
MFAKLVTIASLAILAVATPTPVPQGGSCNTGAIQCCNSVQSADSPAVAGLLG
LLGIVVQDVTAQVGVTCTPISVIGVGGNSCNAQPVCCENNSFNGVVALGCTP
INLNL</td><td rowspan="5">Fomitiporia mediterranea MF3/22</td><td>Class I</td></tr>
<tr><td>68</td><td>>EJD01667.1|Fm.hydrophobin-2
MGIFLGVPESYKSSGFSTYFSSVTQLTSSLTTNSSLELLKYLYSVTMLFTKF
ASFVSLALLAAATPMPNGGSPTKIPPTTTVTVTATATATEPASQCNTGPIQC
CNTVTTAGDSAAAGILGLLGIVVQDLDVLVGLTCTPITVIGTGGTSCNAHPV
CCEDNSHGDLISIGCVPVDISL</td><td>Class I</td></tr>
<tr><td>69</td><td>>EJD01687.1|Fm.hydrophobin-3
MLFTKFATFISLSLLAVATPMPNGGSPTKIAPTTTVTVTATATTTEPASQCN
TGPIQCCNTVTTAGDSAAAGILGLLGIVVQDLDILVGLTCTPITIIGTGGAS
CNAHPVCCEDNSHGDLISIGCVPVDISL</td><td>Class I</td></tr>
<tr><td>70</td><td>>EJC98269.1|Fm.hydrophobin-4
MRFAKYTSLFALPLLAVATPWANPTKTVTVTAVPTTTQPASQCYTGHIQCCN
SVQSASDPAVTTLLGLLGIVLSDLDVLVGLTCTPISVIGAGGNSCSAQPVCC
QNNNFNGLIAIGCTPINLGL</td><td>Class I</td></tr>
<tr><td>71</td><td>>EJD00379.1|Fm.hydrophobin-5
MFSKFAALASLALLAVATPTPGGEPASQCSTGPVQCCNTVTTAGDPAAAGVL
ALIGVVVQDLTVPVGLTCDPITVIGAGGSGCSANPVCCEDNSHGGLISIGCV
PVDLSL</td><td>Class I</td></tr>
<tr><td>72</td><td>>R7SXZ2|Ds.hydrophobin-1
MFSRLAAFVVVALPILAAATPLEGRNEPASSCATAPVQCCDTVGKASDPSIS
KELGLLGVVVQDVDALVGLTCSPITVIGVGSGSSCSANAVCCDDNSFGGLVS
IGCVPIDL</td><td rowspan="7">Dichomitus squalens LYAD-421 SS1</td><td>Class I</td></tr>
<tr><td>73</td><td>>EJF63702.1|Ds.hydrophobin-2
MFARVAVVATLFALPLLAAAGNCNTGGVQCCESYEDSKSAAGATLLKAIGVN
LQDVTGQIGFKCSPISVIGAGGNSACSQQPVCCQNNNVGGAISIGCLPIEL</td><td>Class I</td></tr>
<tr><td>74</td><td>>EJF63706.1|Ds.hydrophobin-3
MFARAAAFSLLALPIFAAASPVRRGNCNTGSVQCCNSLESSDNNAVSLLLGL
LGVTLQGITGSVGLQCSPLQVVGLGSGSACSQQPVCCQNNNVGGLISIGCVP
IEL</td><td>Class I</td></tr>
<tr><td>75</td><td>>EJF58023.1|Ds.hydrophobin-4
MLTHALALAVASLPILAAATPVELEARQSCSTGEIQCCESTETVSISRLCTA
LLGLLGVVVQDLNVLLGVNCSPITVIGVGSSGSCSASTVCCDDNSHGELVSI
GCVPVSL</td><td>Class I</td></tr>
<tr><td>76</td><td>>EJF63704.1|Ds.hydrophobin-5
MFSRAVVVLYFLLSLSILAAAKPWNTTPPPVTTTATATVTVTVSAPAPTGGD
TCSTGPIQCCNSVGSASDPAFSGILGLLGIVLEGVEALVGLGCSPISVVGVG
SGNACSATTVCCQNNSVGGLISIGCIPIIL</td><td>Class I</td></tr>
<tr><td>77</td><td>>EJF63708.1|Ds.hydrophobin-6
MFPRVAAFALVALPLLAAATPLEGRGEPASSCSTGPVQCCNTVTTASDPAAA
GILGLLGIVLQDLTVAVGLSCSPISVIGVGTGNACSANAVCCQDNSLGGLIS
IGCVPVTL</td><td>Class I</td></tr>
<tr><td>78</td><td>>EJF64736.1|Ds.hydrophobin-7
MFSKLAITAALSALLAVATALPAPKDGDSGGKCNTGEIHCCNTVGRSDDSSF
TKYLTPLVQEAIKDLTTVIGVDCDPITVIGATLAGTCSAHPVCCENNSTGGL
VNIGCVPVTA</td><td>Class I</td></tr>
</table>

79	>EJF55662.1|Ds.hydrophobin-8<br>MRSFTLVMAVIAFLVATVAAAAAPGTNAERMARGLPPKAPRKLYRKDPTPAS<br>PARRSTPSGSPSSCSTGPVQCCNTLTTASDPLASVILGLLGIILGPDVAVGL<br>TCSPISVIGVGGGSCSANTVCCENNNVGGLISIGCIPIIL		Class I
80	>EJF64478.1|Ds.hydrophobin-9<br>MKFTYAVAALVAAAASVQATETNAQRLARGLSPLPPRNYHRRHAPSRTEVAK<br>RATPSGTPSEGDGNCNTGPVQCCNQVSKANNNVVAAILGLLGLGGIADDILV<br>GVQCSPLSVVGVGAGSTCSQRPVCCENNSHGGLISIGCIPST		Class I
81	>EJF63705.1|Ds.hydrophobin-10<br>MFARAFAYAALALPLLAVATPVEVRNDASSCSTGSIQCCSSVQSSDSASSNI<br>ILGLLGIVLGDITGLIGLGCSPITVVGVGSGNACSANAVCCTNNNVGGLISI<br>GCLPIEL		Class I
82	>EJF60419.1|Ds.hydrophobin-11<br>MFSRAVVAFCFLLPLSVPAAAKPWNNTPPVSTTATATVTVTVSAPAPTPTSG<br>DTCSTGPVQCCNTVGSANEPPFSGILGLLGIVVEGAESMLGLGCSPMSVDDD<br>GSGTGCSFNVVCCQNNNVGGLFSIGCMLIIL		Class I
83	>EJF60591.1|Ds.hydrophobin-12<br>MPGGNPPPPTQTITVTQTASGPEPTSSCSTGAIQCCESMTKARYPTATKILG<br>LLGIVVEGLDVLVGLTCSAIEVIGVGGGACSSNVVCCQDNSHNSLISIGCLP<br>IIL		Class I
84	>A0A4Q9PA35|Ds.hydrophobin-13<br>MPGGSPPASPTKTVTFTAPAASSTSAGSCSTGAIQCCHSVQSASDPAASLLL<br>GLLGIVVQGVDVLVGLTCSPITVIGVGGGECSATAVCCQDNSHGGLISVGCL<br>PISL		Class I
85	>GbPER71|Hydrophobin-2<br>MFSRVSTLALVALPVLAAATPLEVRGGEPPSSCSTGPIQCCNTVEKASSSGA<br>AAILGLLGIVLQDLNVLVGLTCSPISVIGVGGSNSCSATAVCCQNNNVGGLI<br>SIGCVPVQL		Class I
86	>GbTK4067-66|Hydrophobin-4<br>MFSRAVAFTTLALPLLAVATPVELVARQSCSTGAVQCCNSTVEANSASANLL<br>LGLLGIVLGDITGLIGLNCSPLTVIGVGSGNACSAQAVCCTNNNVGWFDLHW<br>LRPRHAL		Class I
87	>GbTambisan-B|Hydrophobin-4<br>MFSRAVAFTTLALPLLAVATPVELVARQSCSTGAVQCCNSTVEANSASANLL<br>LGLLGIVLGDITGLIGLNCSPLTVIGVGSGNACSAQAVCCTNNNVGWFDLHW<br>LRPRHAL		Class I
88	>Gb4626IIHP|Hydrophobin-4<br>MFSRAVAFTTLALPLLAVATPVELVARQSCSTGAVQCCNSTVEANSASANLL<br>LGLLGIVLGDITGLIGLNCSPLTVIGVGSGNACSAQAVCCTNNNVGWFDLHW<br>LRPRHAL		Class I
89	>GbFTM9SI|Hydrophobin-4<br>MFSRAVAFTTLALPLLAVATPVELVARQSCSTGAVQCCNSTVVGQLGVRQLA<br>PWSAWHCPRGHHWPHWPELLAPHRHRCWQRQRLLRPGCLLHQQQCRWFDLHW<br>LRPHHAL	*Ganoderma boninense*	Class I
90	>GbS6PIIM|Hydrophobin-4<br>MFSRAVAFTTLALPLLAVATPVELVARQSCSTGAVQCCNSTVEANSASANLL<br>LGLLGIVLGDITGLIGLNCSPLTVIGVGSGNACSAQAVCCTNNNVGWFDLHW<br>LRPRHAL		Class I
91	>GbTK3436-342|Hydrophobin-4<br>MFSRAVAFTTLALPLLAVATPVELVARQSCSTGAVQCCNSTVEANSASGNLL<br>LGLLGIVLGDITGLIGLNCSPLTVIGVGTGNACSAQAVCCTNNNVGGLISIG<br>CLPITLQVLQHTKRCLRYGNWMG		Class I
92	>GbUMAS-6B|Hydrophobin-4<br>MFSRAVAFTTLALPLLAVATPVELVARQSCSTGAVQCCNSTVEANSASGNLL<br>LGLLGIVLGDITGLIGLNCSPLTVIGVGSGNACSAQAVCCTNNNVGGLISIG<br>CVPITL		Class I
93	>GbTK3645-36|Hydrophobin-4<br>MFSRAVAFTTLALPLLAVATPVELVARQSCSTGAVQCCNSTVEANSASGNLL<br>LGLLGIVLGDITGLIGLNCSPLTVIGVGSGNACSAQAVCCTNNNVGWFDLHW<br>LRPHHAL		Class I

94	>GbTK3646-39|Hydrophobin-4<br>MFSRAVAFTTLALPLLAVATPVELVARQSCSTGAVQCCNSTVEANSASGNLLLGLLGIVLGDITGLIGLNCSPLTVIGVGSGNACSAQAVCCTNNNVGGLISIGCLPIPLQVPQHPQRCLRNRNWRG		Class I
95	>GbFTM9SI|Hydrophobin-2<br>MFSRVSTLALVALPVLAAATPLEVRGGEPPSSCSTGPIQCCNTVEKASSSGAAAILGLLGIVLQDLNVLVGLTCSPISVIGVGGSNSCSATAVCCQNNNVGGLISIGCVPVQL		Class I
96	>Gb4626IIHP|Hydrophobin-2<br>MFSRVSTLALVALPVLAAATPLEVRGGEPPSSCSTGPIQCCNTVEKASSSGAAAILGLLGIVLQDLNVLVGLTCSPISVIGVGGSNSCSATAVCCQNNNVGGLISIGCVPVQL		Class I
97	>GbUMAS-6B|Hydrophobin-2<br>MFSRVSTLALVALPVLAAATPLEVRGGEPPSSCSTGPIQCCNTVEKASSSGAAAILGLLGIVLQDLNVLVGLTCSPISVIGVGGSNSCSATAVCCQNNNVGGLISIGCVPVQL		Class I
98	>GbTambisan-B|Hydrophobin-2<br>MFSRVSTLALVALPVLAAATPLEVRGGEPPSSCSTGPIQCCNTVEKASSSGAAAILGLLGIVLQDLNVLVGLTCSPISVIGVGGSNSCSATAVCCQNNNVGGLISIGCVPVQL		Class I
99	>GbTK4067-66|Hydrophobin-2<br>MFSRVSTLALVALPVLAAATPLEVRGGEPPSSCSTGPIQCCNTVEKASSSGAAAILGLLGIVLQDLNVLVGLTCSPISVIGVGGSNSCSATAVCCQNNNVGGLISIGCVPVQL		Class I
100	>GbTK3646-39|Hydrophobin-2<br>MFSRVSTLALVALPVLAAATPLEVRGGEPPSSCSTGPIQCCNTVEKASSSGAAAILGLLGIVLQDLNVLVGLTCSPISVIGVGGSNSCSATAVCCQNNNVGGLISIGCVPVQL		Class I
101	>GbTK3645-36|Hydrophobin-2<br>MFSRVSTLALVALPVLAAATPLEVRGGEPPSSCSTGPIQCCNTVEKASSSGAAAILGLLGIVLQDLNVLVGLTCSPISVIGVGGSNSCSATAVCCQNNNVGGLLSIVCAPVHD		Class I
102	>GbS6PIIM|Hydrophobin-2<br>MFSRVSTLALVALPVLAAATPLEVRGGEPPSSCSTGPILCCNTVEKVSHRMIIHLNFFKRSPTQLVLYAISSGAAAILGLLGIVLQDLNVLVGLTCSPISVIGVGGSNSCSATAVCCHNNNVLSTRAVELLPGGLISIGCVPVQL		Class I
103	>EIW52202.1|Tv.hydrophobin-1<br>MFAKLFTLSALAILAVATPAPAADASCSTGAIQCCDSVQKADSAAVAPILAGLGVVVQDVNALVGLTCSPISVVGVGGSDACSANAVCCQDNSFGGLVSIGCAPVSL		Class I
104	>EIW54768.1|Tv.hydrophobin-2<br>MFAKLALFTAAVFTALAAATPIPQGSGSCNTGPITCCEDVRSADDASVATLLGLLGVVVQDVTAQVGIQCSPINVIGAASGNACSATPVCCENNNVGGLISVGCVPVIL		Class I
105	>EIW55689.1|Tv.hydrophobin-3<br>MFARVAVASVLALPLLAAAQSCNTGPIQCCNSVEKSDSAAGSAILSLLGVVLGDITGSIGLGCSPLSVVGLGQSSCSASPVCCQNNNVGGLISIGCLPIEL	*Trametes versicolor* FP-101664 SS1	Class I
106	>EIW55693.1|Tv.hydrophobin-4<br>MFARVAAFTVLALPVFAAATPAVLRRGDCNTGSIQCCNSVHETNENAVSVLLSLLGLDAGSLTGSVGLQCSGISGVGAGQDSCQASPVCCTNNNVGGLISVGCIPIIL		Class I
107	>EIW55696.1|Tv.hydrophobin-5<br>MISARLTAFIFGALPLLAVATPLDTRAAGKCSTGPLQCCESTQKATDPATSVLLALIGVNAQSVDALVGLDCSPISVVGVATGNSCNAQTVCCKNNNLGGLVSLGCVPVSLSN		Class I

108	>EIW61046.1|Tv.hydrophobin-6<br>MFSRAIALTFAALPLLAVATPLEARNDAPASSCSTGALQCCESTTTAGSKSA<br>AAILGLLGVVVQDVTAIVGLSCSPITVVGVGAGSSCSADTVCCEDNSFGGLI<br>SIGCLPVSL		Class I
109	>EIW52199.1|Tv.hydrophobin-7<br>MFAKLFTVASIAILAAATPTPGGEPASSCSTGDLQCCDSVEKASDPAAAGIL<br>ALLGIVVQDVDAMVGLTCDPITVIGVGSSGSCSAQAVCCSDNSHGSAVSIGC<br>VPVTL		Class I
110	>EIW55694.1|Tv.hydrophobin-8<br>MSSRLTAFVLGALPLLAAATPLDTRAAGKCSTGSLQCCNSVQKANDPATSLL<br>LALLGIDVQSIDALVGITCSPITVVGVATGNSCNAQAVCCKNNNVGGAVSLG<br>CVPVTL		Class I
111	>EIW61062.1|Tv.hydrophobin-9<br>MFSRAVALTISALPLLAAATPLEARNSGSCSTGPVQCCQSTETAGSTAGSAL<br>LGSLGIVVQDVNVLVGVTCSPITVVGVGSGGSCSAQTVCCSDNSHGGLVSIG<br>CAPVTV		Class I
112	>EIW52193.1|Tv.hydrophobin-10<br>MFAKLFTLFALAILAVATPAPAADASCSTGALQCCQSVEPAGSEAVAPILAG<br>LGVVVQDLNIPVGVTCTAITVVGVGSGSSCSENTVCCDDNSFGGLVSIGCLP<br>ASV		Class I
113	>EIW61059.1|Tv.hydrophobin-11<br>MSFSRAIAFAIVATPLLAVATPLEARQTCSTGAIQCCQQTTTANSASAAAIL<br>GLLGIVLQDLNVPVGLTCSPISVVGVGSGNGCSANAVCCEDNSHGGLISIGC<br>LPVSL		Class I
114	>EIW61083.1|Tv.hydrophobin-12<br>MFSRTLALTISALPLLAVATPLEARQDCSTGALQCCDSTETAGSAAGAALLG<br>LLGIVVQDVNVLLGVNCSPITVIGVGAGSSCSAQAVCCEDDSHGGLVSIGCV<br>PVTL		Class I
115	>EIW61030.1|Tv.hydrophobin-13<br>MFARVAALALALPILAAAQSCNTGPVQCCNSLEKSDSAAGSAILSTLGLGLG<br>DVTGQIGLQCSPLSAAGVGSSSCKSSPVCCQNNNVGGAVSIGCVPIIL		Class I
116	>EIW57768.1|Tv.hydrophobin-14<br>MKFAYAVAALVATAASVQAGETNGQRLARGLSPMPPKRMYHPSRASEAKRAT<br>PSGTPPSGGSGNCNTGPVQCCNSVQKAGTGGVIGAILGLLDVVLDPEALVGL<br>QCSPLSAVGVGGSSCSSTPVCCENNSQGGLISIGCIPIIL		Class I
117	>EIW55692.1|Tv.hydrophobin-15<br>MFSRVAVFATLALPLLAVATPVELEARQTCSTGAIQCCNSVQSSSSVSGNFL<br>LGLLGIVLGDVTGLIGLDCSPLTVVGVGSGNACSANAVCCTNNNVGGLISIG<br>CLPVIL		Class I
118	>EIW55701.1|Tv.hydrophobin-16<br>MVARLSTALTVASIALLAVATPNGKRWGTTTASKPQSTPAKTVTVTKTAPAS<br>GPTEPASSCSTGPIQCCNSVQSASSPAGSLLLGLLGVVVQGVDVLLGLDCSP<br>ISVIGVGGGECNANAVCCQNNAVGGLISIGCVPVIL		Class I
119	>EIW52215.1|Tv.hydrophobin-17<br>MFAKTTAVFTAFTALAILAVASPTPQAGSCSTGALQCCNSVQSADSLGIAPI<br>LAAIGVILQDINIPIGVTCSPISVVGVGSGNACSSNAVCCENNSFGGLLSIG<br>CLPAIL		Class I
120	>EIW55695.1|Tv.hydrophobin-18<br>MPGGAPPPKTTAAPPPAPTAPAGGSSCSTGAIQCCNSVQSSSSASASAILGL<br>LGIVLGDITGLIGLGCSP		Class I
121	>EIW52195.1|Tv.hydrophobin-19<br>MFAKFFTIASLAILAAATPTPGGEPASSCSTGPVQCCDSTAKANSAAATSIL<br>DSIGVVVQDVNALVGLDCINVVGVGSGNACSANAVCCTNNNVGGLISIGCLP<br>VIL		Class I

122	>EIW55691.1|Tv.hydrophobin-20<br>MFSRAVATLYFVLSLTLLAVALPGGKPPPPTTTITATGPPPTSTPGDLCSTG<br>NVQCCQSVGGADDHVIGALLGLLGVVVDAVDVVLGLQCSPIKIVGLGTDKAC<br>SSNVVCCENNSIGGLISIGCIPIIL		Class I
123	>EIW55742.1|Tv.hydrophobin-21<br>MFSRLPTVVAFASLAVLAVATPMEVKRGGAPPPVTKTVTVTAPAATPTGGSC<br>STGPIQCCNSVQSSKSDAGSLLLGLLGIVVQGVDVLLGVGCSPISVIGVGGG<br>SCNANTVCCQNNNVGGLISIGCVPIIL		Class I
124	>Q2U3W7|Ao.hydrophobin-1<br>MQFSVAAVLALATAVAALPPASGTGAGQQVGHSKNDFPLPKELTTKQAADKC<br>GDQAQLTCCNKTVKTGDFTQVEEGLLAGLLSNLLGAGQGSQGLGLLDECTNI<br>PVIPIISIASPQEKCKQPISCCQNTKSSADGDLVGIGLPCIALGSLL	*Aspergillus oryzae*	Class I
125	>A0A219TTW8|Th.hydrophobin-1<br>MKFFAVAALFVASAMAGPMGSEGCPGGLTGTVPLCCATNVLNIADLDCSTPT<br>IPVPNVGIFQAHCASKGKQPVCCTLPVAGQGLLCNKPTGAQ	*Trichoderma harzianum*	Class II
126	>P52755|Th.hydrophobin-2<br>MKFLTVAAVFFTAVLAAPGNYPPPPPPTYAPPPPTYTLPPNGNGGGNGNGNG<br>NGNGGGNGNGNGNTNTGGSALCPAGLYSNPQSCATDVLGLADLDCAVPSTTP<br>HDGPNFQSICVANGGKRARCCVLPVLGLGVLCQNPVGTN		Class II
127	>P79073|Tr.hydrophobin-1<br>MQFFAVALFATSALAAVCPTGLFSNPLCCATNVLDLIGVDCKTPTIAVDTGA<br>IFQAHCASKGSKPLCCVAPVADQALLCQKAIGTF	*Trichoderma reesei*	Class II
128	>G0RVE5|Tr.hydrophobin-1<br>MQFLAVAALLFTTALAAPSSDVNGIIRRANAFCPEGLLYTNPLCCDLDVLGV<br>ADVDCVVPPAKPSSCKSFGSVCASIGRKPRCCAVPVAGVALLCTDPIPAI		Class II
129	>J4KPV6|Bb.hydrophobin-1<br>MRFALAITTLIAAVTAAPSGHHGGKDLHKMTLGQAGDRCGADQSIYCCNEKT<br>TKIEHSSPETGLGGLLGATVGADGLLSHLLGTCTKIPVNVLAVGNLLQSECT<br>NRAACCHNTPSVAYGGLVNLALPCVAIGSLIQ	*Beauveria bassiana*	Class I
